# Mass spectrometry-based metabolomics uncovers distinct metabolic signatures and potential therapeutic targets in *Plasmodium knowlesi*

**DOI:** 10.1371/journal.pone.0337058

**Published:** 2025-11-13

**Authors:** Naphatsamon Uthailak, Sadudee Chotirat, Ammarind Anatjitsupha, Waraporn Thongyod, Phornpimon Tipthara, Jetsumon Sattabongkot, Joel Tarning, Wang Nguitragool, Onrapak Reamtong

**Affiliations:** 1 Department of Social and Environmental Medicine, Faculty of Tropical Medicine, Mahidol University, Bangkok, Thailand; 2 Mahidol Vivax Research Unit, Faculty of Tropical Medicine, Mahidol University, Bangkok, Thailand; 3 Mahidol Oxford Tropical Medicine Research Unit, Faculty of Tropical Medicine, Mahidol University, Bangkok, Thailand; 4 Centre for Tropical Medicine and Global Health, Nuffield Department of Clinical Medicine, University of Oxford, Oxford, United Kingdom; 5 Department of Molecular Tropical Medicine and Genetics, Faculty of Tropical Medicine, Mahidol University, Bangkok, Thailand; Universidade Federal de Minas Gerais, BRAZIL

## Abstract

Malaria remains a major global health challenge, caused by several *Plasmodium* species and transmitted via mosquito vectors. Among these, *Plasmodium knowlesi* is notable for its zoonotic nature, capable of infecting both macaques and humans. The incidence of *P. knowlesi* infections has been rising, particularly in Southeast Asia, raising public health concerns. However, compared to other *Plasmodium* species, the biology, pathophysiology, and transmission dynamics of *P. knowlesi* remain poorly understood. Given the absence of a licensed vaccine and the increasing threat of drug resistance, a deeper understanding of *P. knowlesi* biology is essential for effective control and management strategies. This study investigates the metabolomic landscape of *P. knowlesi* across three intraerythrocytic stagesring, trophozoite, and schizont using mass spectrometry-based metabolomics to gain insights into parasite biology. The analysis revealed distinct metabolic profiles, particularly in the ring stage compared to the other two stages. While glycerophospholipid metabolism and sphingolipid de novo biosynthesis emerged as key pathways associated with common metabolites across all stages, phosphatidylserine synthesis was specifically linked to ring-stage-biased metabolites. Notably, CDP-diacylglycerol-inositol 3-phosphatidyltransferase was highlighted as a promising target based on shared and stage-biased metabolites. Collectively, our findings offer a comprehensive metabolomic profile of *P. knowlesi* blood-stage development, enhancing our understanding of its biology and identifying potential drug targets that could support the development of novel therapeutic strategies against *P. knowlesi* malaria.

## Introduction

Malaria is a vector-borne disease caused by various species of protozoan parasites belonging to the genus *Plasmodium* [1]. It is globally found in tropical and subtropical areas, with an estimated 263 million cases and 597,000 deaths worldwide reported by the World Health Organization (WHO) [2]. Over 200 *Plasmodium* species have been identified with their specific hosts (mammals, birds, and reptiles) [1,3]. Among them, five species are the common cause of malaria in humans, including *P. falciparum*, *P. vivax*, *P. malariae*, *P. ovale*, and *P. knowlesi* [4]. Of these five species, *P. falciparum* and *P. vivax* are predominant species causing global public health issues, and *P. knowlesi* is the only species with zoonotic transmission [5–7]. The predominant natural hosts of *P. knowlesi* are long-tailed (*Macaca fascicularis*) and pig-tailed (*Macaca nemestrina*) macaques, and parasite transmission to humans naturally occur via *Anopheles* mosquitoes, notably the Leucosphyrus group [7]. According to experimental infections, *P. knowlesi* can be transmitted from humans to humans via mosquitoes [8], but the evidence of sustained non-zoonotic transmission in endemic areas is still lacking [9,10]. Thus, most cases of *P. knowlesi* malaria are likely a spillover from the zoonotic sources. Recently, many cases of human knowlesi malaria have been reported in Southeast Asia, including Malaysia, Thailand, Myanmar, Philippines, Singapore, Vietnam, Indonesia, and Cambodia. Thus, it has become a significant public health concern [11]. The region is the epicenter for the travel-related infections of *P. knowlesi* worldwide [12,13].

Clinical symptoms of malaria are caused by red blood cell infection. In the bloodstream, the parasite invades red blood cells and progresses through the ring stage, trophozoite stage, and schizont stage. This intra-erythrocytic development culminates in the formation of merozoites, which are released from the infected cell to invade additional red blood cells. The blood cycle takes approximately 24 h for *P. knowlesi*, which is much shorter than 48 h for *P. falciparum*, *P. vivax*, and *P. ovale* and 72 h for *P. malariae* [14]. As a result, *P. knowlesi* parasitemia can rise rapidly, leading to severe illness if the patient is not treated promptly.

RTS,S/AS01 and R21/Matrix-M™ are the first and second malaria vaccines available to date. They have been introduced to the African continent to reduce illness and death caused by *P. falciparum*. No vaccine is currently approved to prevent non-falciparum malaria. Early diagnosis and correct identification of *Plasmodium* species are crucial for effective treatment and case management [15]. A wide range of antimalarial drugs has been utilized over time, including quinine and its derivatives, chloroquine, sulfadoxine-pyrimethamine, mefloquine, artemisinin, and artemisinin-based combination therapies (ACTs) [16]. Chloroquine and artemisinin-based combination therapy (ACT) are both effective treatments for uncomplicated *P. knowlesi,* in which ACT has been shown to clear parasites more rapidly and is linked to a reduced incidence of anaemia compared to chloroquine [17]. However, the rise of drug resistance for malaria has significant concerns about the sustainability of current antimalarial treatments, in particular, the increasing artemisinin resistance across Southeast Asia [18]. The increasing resistance has made malaria treatment more challenging and emphasized the need for the discovery of more potent antimalarial drugs [19]. Even though drug resistance has not been observed in field studies, several investigations have focused on identifying potential drug targets and discovering novel antimalarial compounds. However, the majority of research efforts have been directed toward *P. falciparum* [20–22]. Relatively few studies have investigated the identification of novel drug targets for *P. knowlesi* infection [23,24]. Given the rise of *P. knowlesi* burdens in the human population [25,26], the identification of novel potential drug targets for *P. knowlesi* is increasingly recognized as essential for reducing disease complications and supporting antimalarial drug discovery efforts, thereby contributing to global malaria control initiatives [23,27].

Mass spectrometry (MS)-based metabolomics is a useful technology that is widely applied in medical science and clinical research. It is a powerful tool in drug discovery and development and has an expanded role in biomarker discovery for disease diagnosis and prognosis, and treatment monitoring [28]. Additionally, it can provide insights into biological processes, metabolic pathways, and potential disease mechanisms [29]. Previously, potential drug targets were identified through kinome profiling across eight *Plasmodium* species, including *P. knowlesi.* However, these targets were primarily highlighted as promising candidates for *P. vivax* [24]. To date, no comprehensive metabolite profiling of *P. knowlesi* has been reported, leaving a critical gap in our understanding of its metabolic pathways and potential therapeutic vulnerabilities. In this study, we present the metabolomic profiles of *P. knowlesi* across three distinct erythrocytic stages: ring, trophozoite, and schizont. This stage-specific profiling provides valuable insights into the parasite’s biology and potential avenues for therapeutic intervention. The analysis reveals metabolic adaptations unique to each stage and highlights essential pathways involved in parasite survival and replication. By identifying both stage-specific and shared metabolites, we gain a clearer understanding of developmental transitions and core biological functions. Notably, this approach facilitates the identification of novel drug targets by uncovering key enzymes and metabolites critical to the parasite’s intraerythrocytic development.

## Materials and methods

### Cultivation of parasites

The *P. knowlesi* strain A1-H.1 used in this study [30] was provided by Dr. Robert W. Moon from the Division of Parasitology at the Medical Research Council National Institute for Medical Research in London. This study was granted exemption by the Ethics Committee of the Faculty of Tropical Medicine, Mahidol University. The parasites were cultured in a complete medium (pH 7.4) composed of RPMI-1640 (Invitrogen), 5.96 g/L HEPES, 2.3 g/L sodium bicarbonate, 2 g/L D-glucose, 0.292 g/L L-glutamine, 0.05 g/L hypoxanthine, 5 g/L Albumax II (Invitrogen), 0.025 g/L gentamycin sulfate, and 10% (vol/vol) horse serum (Invitrogen). Human red blood cells were sourced from the Thai Red Cross at 50% hematocrit, then washed with RPMI-1640 containing 2 g/L sodium bicarbonate, 5.94 g/L HEPES, 2 g/L D-glucose, and 0.025 g/L gentamycin sulfate. RBCs were added to achieve a 2–5% hematocrit, and the culture was maintained in 75 cm² flasks at 37°C in a gas mixture of 90% N2, 5% CO2, and 5% O2. Rings, trophozoites, and schizonts were fractionated from asynchronous culture by centrifuging the infected erythrocytes through a 70%/40% Percoll step gradient. Schizonts were collected from the top phase (density <40%), trophozoites from the middle (40%/70%) interface, and rings from the bottom phase (density >70%). Light microscopy confirmed that the majority of parasites in each fraction were at the expected developmental stage. The collected pools of parasites at each stage were treated with 1 ml of 0.15% saponin in phosphate buffer saline (PBS) to lyse the host cells and deplete the host cytosol. The parasites were washed twice with cold PBS before freezing down and stored at −80°C until use.

### Metabolite extraction

Metabolome was extracted according to the previous study [31]. For parasite samples, the 10 milligrams of each stage of *P. knowlesi* (ring, schizont, and trophozoite) were homogenized in 500 µl of methanol. After freeze-thawing and grinding in liquid nitrogen, supernatants were collected by centrifugation at 800 g, 4°C for 1 min. This process was repeated to extract the second supernatant. The pellet was then dissolved in 250 µL of deionized water, followed by freeze-thawing in liquid nitrogen. After centrifugation at 15,000 g, 4°C for 1 min, the third supernatant was collected and combined with earlier supernatants. The remaining debris was removed by centrifugation at 15,000 g, 4°C for 1 min. The supernatant was then collected and dried using a speed vacuum (Tomy Digital Biology, Tokyo, Japan). For patient blood samples, 80 µl of cold methanol was mixed with 20 µl of blood for 1 min by vortexing. After incubation at 4°C for 20 min, the supernatant was collected by centrifugation at 12,000 rpm for 10 min and dried in a speed vacuum. All dried metabolites were stored at −80°C for further experiments.

### Metabolomics analysis using LC-MS/MS

Extracted metabolome was analyzed using an HPLC-ESI-Q-TOF mass spectrometer, following the previous study [31]. Briefly, metabolome samples were applied to a high-performance liquid chromatography (HPLC; Agilent 1260 Quaternary Pump, Agilent 1260 High Performance Autosampler and Agilent 1290 Thermostatted Column Compartment SL, Agilent Technologies, CA, USA) combined with a quadrupole time-of-flight mass spectrometer (Q-TOF MS; TripleTOF 5600 + , SCIEX, US) with both positive and negative modes of the electrospray ionization (+ESI, -ESI). For the HPLC, the samples were resuspended in a mixture of mobile phase A (0.1% formic acid in water) and B (0.1% formic acid in acetonitrile) at a ratio of 1:1 (v/v). Samples were then auto-injected into the C18 reversed-phase UHPLC column (ACQUITY UPLC BEH, 2.1 x 100 mm, 1.7 μM, Waters) with the flow rate of 0.3 mL/min at 40°C. For the Q-TOF MS, mass ion chromatograms and mass spectra were obtained using Analyst Software version 1.7 (SCIEX, US). The information-dependent acquisition mode was performed using a TOF-MS scan. The *m/z* range of MS and MS/MS was 100–1000 and 50–1000, respectively. The QC samples were prepared as a mixture of each metabolite with equal aliquoting. The system performance was determined by the injection of QC samples before, during, and after sample analysis.

### Metabolomics data processing and analysis

For metabolomics data processing, the mass spectra from the HPLC-MS/MS (.wiff and.wiff.scan files) in +ESI and -ESI were separately processed using the XCMS online software version 3.7.1 (The Scripps Research Institute, CA, USA). The multigroup analysis and pairwise analysis were applied for parasite samples and blood samples, respectively, with the “UPLC/Triple TOF pos” protocol. For metabolomics data analysis, the acquired data from the XCMS software were analyzed using the MetaboAnalyst online software version 6.0 (https://www.metaboanalyst.ca/) in the statistical analysis (one factor) module [32]. The data filtering was performed using a variance filter (interquartile range; 40%) and abundance filter (median intensity value), followed by data normalization using quantile normalization, cube root data transformation, and data range scaling. For clustering, the principal component analysis (PCA) and partial least squares-discriminant analysis (PLS-DA) were performed with 95% confidence regions. Five biological replications and ten quality control (QC) samples were used in this study. ANOVA was applied for the identification of differential metabolites among parasitic samples (ring, trophozoite, and schizont) with the raw p-value (FDR) cutoff at 0.01. All data were shown with the normalization of concentration using the same amount of parasite (10 mg) for the metabolome extraction, with the final concentration of 20 mg/mL. According to the software, only the top 1000 features will be analyzed in this step. The hierarchical clustering heatmaps were performed with the Euclidean distance measurement and the Ward clustering method. All potential metabolites were annotated using the XCMS online software.

### Pathway and drug target analysis

The metabolic pathway was analyzed using the MetaboAnalyst online software version 6.0 (https://www.metaboanalyst.ca/) in the pathway analysis and enrichment analysis modules [32]. For the specific parameters in pathway analysis, the relative between centrality and hypergeometric test was selected as the topology analysis and enrichment method, respectively. The pathway analysis was identified with statistical significance (p-value < 0.01). For enrichment analysis, RaMP (relational database of metabolomic pathways) was applied as a set library with a statistical significance of p-value < 0.01. The comparison of pathways between humans (*Homo sapiens*) and *P. knowlesi* was conducted using the Kyoto Encyclopedia of Genes and Genomes (KEGG) pathway database (https://www.genome.jp/kegg/pathway.html). The amino acid sequence of *P. knowlesi* proteins from each pathway was analyzed by the Protein BLAST from NCBI (https://blast.ncbi.nlm.nih.gov/Blast.cgi) to identify protein sequences of its homologs in other human-infecting *Plasmodium* species (*P. knowlesi*, *P. falciparum, P. vivax, P. malariae, P. ovale*) and the human host (*H. sapiens*). All protein sequences obtained from the NCBI database are shown in the S1 Appendix. The similarity of amino acid sequence across 6 species was analyzed using the multiple sequence alignment (MSA) by Clustal Omega (https://www.ebi.ac.uk/jdispatcher/msa/clustalo) with the ClustalW format.

## Results

### Identification of metabolites shared across the erythrocytic stages of *P. knowlesi*

The metabolome, representing the ultimate downstream products of the genome and proteome, serves as a complementary dataset to genomes, transcriptomes, and proteomes for gaining a more comprehensive understanding of biology. In this study, the metabolites of three different erythrocytic stages of *P. knowlesi*, including ring, trophozoite, and schizont, were analyzed using MS-based metabolomics. According to the XCMS software, a total of 15,937 features were identified in both positive (9,061 features) and negative (6,876 features) electrospray ionization (ESI) modes. Based on their consistent presence across the three erythrocytic stages, the 20 most frequently detected metabolites were selected and are presented in Table 1. Various lipid species were identified as common metabolites across the samples, including phosphatidylcholine (PC), phosphatidylethanolamine (PE), phosphatidic acid (PA), phosphatidylglycerol (PG) and monoacylglycerol (MG). Additionally, organic acids were also commonly found across all three erythrocytic stages of *P. knowlesi*.

**Table 1:** Top 20 most common detected metabolites across three erythrocytic stages of *P. knowlesi.*

No.	Potential metabolites	*m/z*	RT	Mass error	Adduct	% Average abundance		
						Ring	Trophozoite	Schizont
1	PE(18:4(6Z,9Z,12Z,15Z)/P-18:1(11Z))	721.5045	16.09	0	M+	100.00	100.00	82.34
2	PE(P-16:0/20:5(5Z,8Z,11Z,14Z,17Z))	721.5047	15.81	0	M+	49.29	68.79	100.00
3	PC(O-16:0/13:0)	722.5078	16.09	1	M + 2Na-H	47.80	48.57	37.51
4	PC(O-14:0/15:0)	722.5074	15.81	0	M + 2Na-H	29.01	33.05	49.98
5	PA(19:1(9Z)/0:0)	473.2643	7.67	1	M + Na	26.15	25.87	21.55
6	Butyl 4’-O-butanoyl-6-O-hexadecanoyl-neohesperidoside	708.4873	9.23	3	M + NH_4_	70.58	1.50	0.39
7	PC(22:1(11Z)/0:0)	577.4122	9.34	2	M+	44.10	0.54	0.26
8	Histidylleucine	267.1453	8.69	4	M-H	36.68	1.70	3.19
9	PE(13:0/14:0)[U]	621.4382	9.3	2	M+	38.08	0.65	0.24
10	Oxaloacetate	112.9871	0.3	3	M-H_2_O-H	10.97	10.52	10.38
11	PG(14:0/14:0)	709.4908	9.23	3	M + ACN + H	28.05	0.61	0.20
12	PE(14:0/22:5(4Z,7Z,10Z,13Z,16Z))	737.4993	19.01	0	M+	8.15	8.68	8.64
13	MG(0:0/16:0/0:0)	331.2844	12.08	0	M + H	9.01	8.54	5.90
14	PE(P-18:1(9Z)/18:4(6Z,9Z,12Z,15Z))	721.5045	19.3	0	M+	6.87	8.03	8.22
15	PE(18:3(9Z,12Z,15Z)/18:3(9Z,12Z,15Z))	753.5168	9.2	1	M + NH_4_	21.90	0.50	0.14
16	3-hexanoyl-NBD Cholesterol	663.4511	15.83	5	M + H	4.06	5.80	11.91
17	18-oxo-nonadecanoic acid	313.274	12.08	1	M + H	7.64	7.45	5.45
18	2-Hexyldecanoic acid	255.2328	13.17	1	M-H	6.10	5.27	7.57
19	PG(18:4(6Z,9Z,12Z,15Z)/12:0)	669.4164	9.26	5	M + H-H_2_O	16.94	0.82	0.38
20	PA(16:0/20:4(5Z,8Z,11Z,14Z))	661.461	9.31	1	M + H-2H_2_O	16.27	1.23	0.45

*RT=Retention time (min).

### Pathway analysis of common metabolites across blood stages of *P. knowlesi*

The 100 most abundant metabolites, consistently detected across the ring, trophozoite, and schizont stages of *P. knowlesi*, were selected to explore the shared metabolic pathways involved in its erythrocytic development (Table 2; S1 Table). These metabolites were subjected to comprehensive pathway and enrichment analyses to uncover the key biological processes associated with parasite survival and progression through its intraerythrocytic cycle. Using a significance threshold with a cut-off p-value of <0.01, the analyses revealed that sphingolipid *de novo* biosynthesis and glycerophospholipid metabolism are the most significantly associated pathways in enrichment analysis and pathway analysis, respectively. These findings suggest that lipid biosynthesis might play critical roles throughout the erythrocytic stages of *P. knowlesi*.

**Table 2:** Enrichment analysis and pathway analysis of the top 100 common metabolites across all three erythrocytic stages of *P. knowlesi*. The analysis was performed using the MetaboAnalyst online software with a significance threshold of a cut-off p-value of < 0.01.

	Potential pathways	p-value
Enrichment analysis	Sphingolipid de novo biosynthesis	6.77E-04
	G alpha (q) signaling events	0.00147
	Sphingolipid metabolism	0.00214
	Regulation of actin cytoskeleton	0.00583
	Malate-Aspartate Shuttle	0.00815
Pathway analysis	Glycerophospholipid metabolism	0.007215

### Identification of a potential drug target for *P. knowlesi* across the three erythrocytic stages

**Fig 1:**
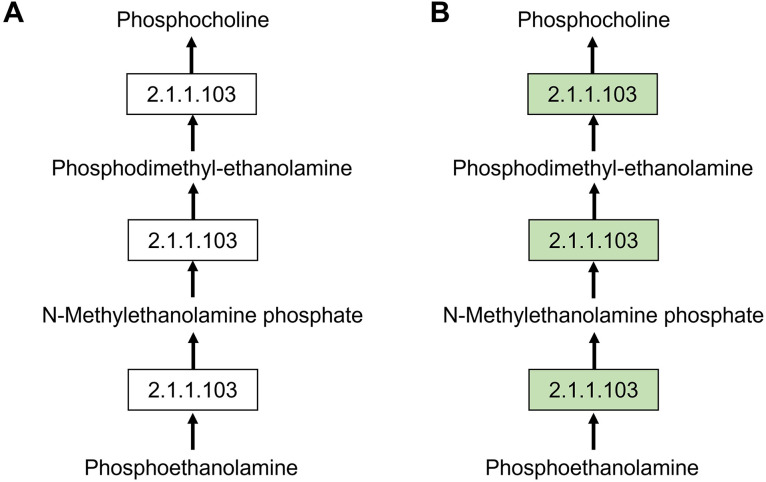
PMT-related steps in glycerophospholipid metabolic pathways of *P. knowlesi* (A) and *H. sapiens* (B). Green boxes indicate the enzymes existing in each species. The figure has been adapted from the KEGG database.

To identify potential drug targets for *P. knowlesi*, a comparative analysis of the glycerophospholipid metabolic pathway the most significantly enriched pathway was conducted between *P. knowlesi* and human using the KEGG database (Fig 1). This pathway-based comparison aimed to pinpoint parasite-specific enzymes that are absent in the human host, thereby minimizing potential toxicity to the human host if used as a therapeutic target. Among the various enzymes involved in glycerophospholipid metabolism, phosphoethanolamine N-methyltransferase (PMT) [EC:2.1.1.103] emerged as a unique enzyme present in *P. knowlesi* but not in humans. PMT is an enzyme involved in the phosphatidylcholine biosynthesis, catalyzing the stepwise methylation of phosphoethanolamine to produce phosphocholine. The absence of this enzyme in humans underscores its potential as a selective and promising drug target for *P. knowlesi* malaria. Although this study primarily targets a drug candidate specific to *P. knowlesi*, the identified drug target should exhibit cross-species applicability across other *Plasmodium* species that cause human malaria. The broad-spectrum potential would enhance the therapeutic value of the drug candidate. To evaluate this, the PMT sequence alignment and percentage of identity among different *Plasmodium* spp and the human host were performed as shown in Table 3. As a result, the PMT sequence of *P. knowlesi* shows a similarity to the PMT derived from other human-infecting *Plasmodium* species, including *P. falciparum* (61.65%), *P. vivax* (87.50%), *P. malariae* (73.00%), and *P. ovale* (72.62%). This finding suggests a conserved functional role of PMT across these species, implying that the inhibitors designed against *P. knowlesi* PMT may have potential cross-reactivity and efficacy against other malaria-causing parasites. This drug target has the potential to affect all intraerythrocytic stages of *P. knowlesi*. Notably, the PMT did not exist in human (*H. sapiens*) host, indicating the low potential risk of host toxicity. Therefore, PMT was considered a promising drug target for *P. knowlesi* infection

**Table 3:** Percentage similarity in protein sequence alignment of PMT among five *Plasmodium* spp. and the human host. All protein sequences were obtained from the NCBI database. The sequence alignment was performed using the multiple sequence alignment by Clustal Omega software. Regions of the highest similarity (>70%; green), high similarity (50-70%; yellow), and low similarity (<50%; red) are indicated.

	*P. knowlesi*	*P. falciparum*	*P. vivax*	*P. malariae*	*P. ovale*
***P. knowlesi***	100.00	61.65	87.50	73.00	72.62
***P. falciparum***	61.65	100.00	64.26	65.78	60.84
***P. vivax***	87.50	64.26	100.00	75.29	74.52
***P. malariae***	73.00	65.78	75.29	100.00	75.29
***P. ovale***	72.62	60.84	74.52	75.29	100.00
***H. sapiens***	N/A	N/A	N/A	N/A	N/A

N/A: Not Available.

### Identification of erythrocytic stage-biased metabolites in *P. knowlesi*

**Fig 2:**
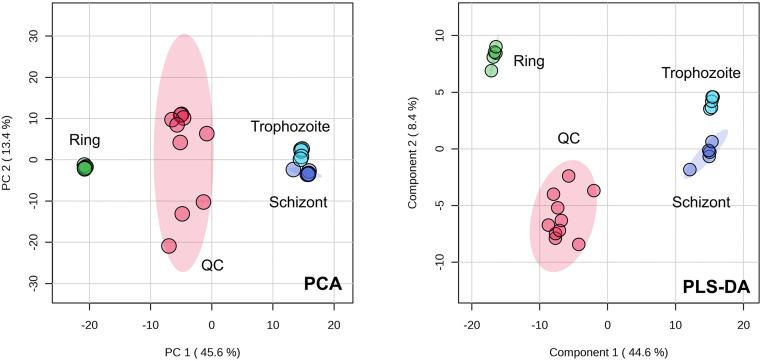
2D-score plot from PCA and PLS-DA comparing the metabolomes derived from three blood stages of *P. knowlesi* (ring, trophozoite, and schizont). The QC sample was a mixture of three blood stage samples with equal aliquoting. Both PCA and PLS-DA were performed with 95% confidence regions using the MetaboAnalyst software. Each circle represents a biological replication of the metabolome (N = 5).

Metabolomics reflects the physiological state of a biological system. This study focuses on stage-specific metabolic changes during the erythrocytic cycle of *P. knowlesi* to uncover molecular mechanisms and identify potential therapeutic targets. Both principal component analysis (PCA) and partial least squares discriminant analysis (PLS-DA) provided an overview of the metabolomic profiles derived from the three blood stages (Fig 2). The results demonstrated a clear separation of the ring-stage cluster from the trophozoite and schizont stages in both PCA and PLS-DA, indicating a unique metabolomic profile. Even though the trophozoite and schizont-stage clusters are in close proximity in PCA, they are more separated in PLS-DA. The reliability of the instrument performance and analytical conditions was confirmed by reasonably tight grouping of the QC samples. The top 20 most abundant metabolites of each *P. knowlesi* stage based on their peak intensities were shown in Table 4. Overall, small peptides and various glycerophospholipids such as phosphatidylethanolamine (PE), phosphatidic acid (PA), phosphatidylglycerol (PG), phosphatidylcholines (PC), and phosphatidylserine (PS) were predominantly found in the list of top 20 most abundant metabolites in all three stages of *P. knowlesi*. However, there were some differences in the most abundant metabolites of each stage. 25-hydroxy-cholesterol(d3) was highly identified in the ring stage. While several acids were found in either the trophozoite or schizont stage, such as palmitic acid, stearic acid, 4,8,12-trimethyl-tridecanoic acid, and isostearic acid.

**Table 4:** Top 20 most abundant metabolites found in *P. knowlesi* ring, trophozoite, and schizont stages.

	Potential Metabolites	m/z	RT (min)	Mass error (ppm)	Adduct	Mode	METLIN	Average peak intensity
**Ring**								
1	PE(18:4(6Z,9Z,12Z,15Z)/P-18:1(11Z))	721.5039	16.40	1	M+	Positive	60621	4413387
2	Lys Lys Pro Val	488.3565	9.40	2	M + NH_4_	Positive	170936	2670054
3	Phe Ile Arg Arg	589.3560	8.50	3	M-H	Negative	138573	1881257
4	25-hydroxy-cholesterol(d3)	444.3304	9.42	3	M + K	Positive	41636	1433350
5	Asp His Lys Arg	553.2850	8.48	0	M-H	Negative	122054	1223302
6	PA(19:1(9Z)/0:0)	473.2640	7.65	0	M + Na	Positive	82344	1154239
7	Lys Lys Pro Val	493.3116	9.40	1	M + Na	Positive	170936	953125
8	Lys Asn Arg Arg	537.3375	9.37	1	M + H-2H_2_O	Positive	172173	892910
9	Leu Leu Asp Leu Leu	603.4081	9.28	1	M + NH_4_	Positive	264741	858962
10	His Ile Arg Arg	581.3637	9.33	1	M + H	Positive	154573	845075
11	Ala Val Val Pro Leu	515.3558	9.35	1	M + NH_4_	Positive	264929	779993
12	PG(12:0/18:4(6Z,9Z,12Z,15Z))	669.4161	9.24	4	M + H-H_2_O	Positive	78881	747564
13	Arg Gly Cys	333.1352	8.44	0	M-H	Negative	22759	720820
14	PA(18:2(9Z,12Z)/18:2(9Z,12Z))[U]	661.4609	9.28	1	M + H-2H_2_O	Positive	40912	717898
15	PA(P-16:0/18:4(6Z,9Z,12Z,15Z))	617.4347	9.33	1	M + H-2H_2_O	Positive	82255	680871
16	PA(16:1(9Z)/22:4(7Z,10Z,13Z,16Z))	705.4877	9.24	2	M + H-H_2_O	Positive	81389	659909
17	Arg Arg Thr Thr	531.3024	8.50	3	M-H	Negative	221415	630247
18	Ile Lys Lys Thr	471.3298	9.40	1	M + H-H_2_O	Positive	162855	628511
19	PG(17:0/20:4(5Z,8Z,11Z,14Z))	749.5137	9.22	1	M + H-2H_2_O	Positive	3878	611913
20	Ile Ile Ile Lys	485.3565	9.42	2	M+	Positive	162427	586614
**Trophozoite**								
1	PE(18:4(6Z,9Z,12Z,15Z)/P-18:1(11Z))	721.5049	16.30	0	M+	Positive	60621	4523840
2	Oxaloacetate	112.9871	0.30	3	M-H_2_O-H	Negative	123	475872
3	Ile Ile Met Val	473.2801	16.33	0	M-H	Negative	162496	411975
4	PE(14:0/22:5(4Z,7Z,10Z,13Z,16Z))	737.4996	19.06	0	M+	Positive	60289	392640
5	MG(0:0/16:0/0:0)	331.2845	12.08	1	M + H	Positive	62317	386274
6	PC(16:0/9:0(CHO))	650.4372	11.05	3	M + H	Positive	82381	376034
7	MG(18:0/0:0/0:0)	359.3155	13.91	0	M + H	Positive	61993	357294
8	MG(16:0/0:0/0:0)	313.274	12.08	1	M + H-H_2_O	Positive	3855	336845
9	Palmitic acid	255.2328	13.17	1	M-H	Negative	187	238499
10	2-oxo-heneicosanoic acid	341.3051	13.91	0	M + H	Positive	74773	237263
11	PE(22:2(13Z,16Z)/22:6(4Z,7Z,10Z,13Z,16Z,19Z))	808.5673	10.95	3	M + H-2H_2_O	Positive	60962	220874
12	Stearic acid	283.2641	14.93	1	M-H	Negative	189	192429
13	Arginyl-Glycine	273.1672	6.44	1	M + ACN + H	Positive	85624	180580
14	PE(18:1(9Z)/0:0)	478.2917	8.82	4	M-H	Negative	40778	177648
15	Gly Lys Met Arg	473.2655	8.17	1	M + H-H_2_O	Positive	146893	176912
16	DG(P-14:0/18:1(9Z))	589.4592	18.33	0	M + K	Positive	4697	176468
17	PC(14:1(9Z)/18:4(6Z,9Z,12Z,15Z))	723.4842	18.30	0	M+	Positive	59360	167655
18	C16 Sphinganine	274.274	1.12	0	M + H	Positive	41556	164463
19	Ile Lys Arg Tyr	543.3416	10.83	0	M + H-2H_2_O	Positive	162978	161596
20	PS(12:0/15:0)	666.4321	9.94	3	M + H	Positive	77711	156425
**Schizont**								
1	PE(18:4(6Z,9Z,12Z,15Z)/P-18:1(11Z))	721.5041	15.81	1	M+	Positive	60621	4600742
2	PA(19:1(9Z)/0:0)	473.2642	7.68	1	M + Na	Positive	82344	989543
3	Oxaloacetate	112.9873	0.30	2	M-H_2_O-H	Negative	123	477582
4	PE(14:0/22:5(4Z,7Z,10Z,13Z,16Z))	737.4993	18.99	0	M+	Positive	60289	397351
5	Phe Ile Pro Val	473.2775	16.26	1	M-H	Negative	138536	389265
6	4,8,12-trimethyl-tridecanoic acid	255.2319	13.15	4	M-H	Negative	34669	348136
7	MG(0:0/16:0/0:0)	331.2845	12.08	1	M + H	Positive	62317	271593
8	Isostearic acid	283.263	14.91	4	M-H	Negative	4293	253930
9	MG(18:0/0:0/0:0)	359.3155	13.86	0	M + H	Positive	61993	248820
10	DG(P-14:0/18:1(9Z))	589.4591	18.27	0	M + K	Positive	4697	193170
11	PC(14:1(9Z)/18:4(6Z,9Z,12Z,15Z))	723.484	18.26	0	M+	Positive	59360	181734
12	Ile Lys Lys Tyr	533.3451	16.43	0	M + H-H_2_O	Positive	162858	179864
13	His Ile Ile Ile	517.3107	15.39	0	M + Na	Positive	154426	178411
14	Ala Phe Pro Val	432.2375	7.68	0	M+	Positive	105337	173101
15	2-oxo-heneicosanoic acid	341.3048	13.86	1	M + H	Positive	74773	172308
16	Phe Ile Lys Gln	517.3137	13.76	0	M + H-H_2_O	Positive	138452	156354
17	Ala Glu Ile Lys	459.2691	15.18	0	M+	Positive	104828	146595
18	His His Lys Val	500.2733	8.03	0	M-H_2_O-H	Negative	154056	139733
19	Ala Ile Lys Ser	417.2586	19.29	0	M+	Positive	106455	135877
20	Ala Ile Ser Lys	417.2592	18.34	1	M+	Positive	106588	134447

*RT: Retention time.

### Relative quantification of metabolites among *P. knowlesi* erythrocytic stages

**Fig 3:**
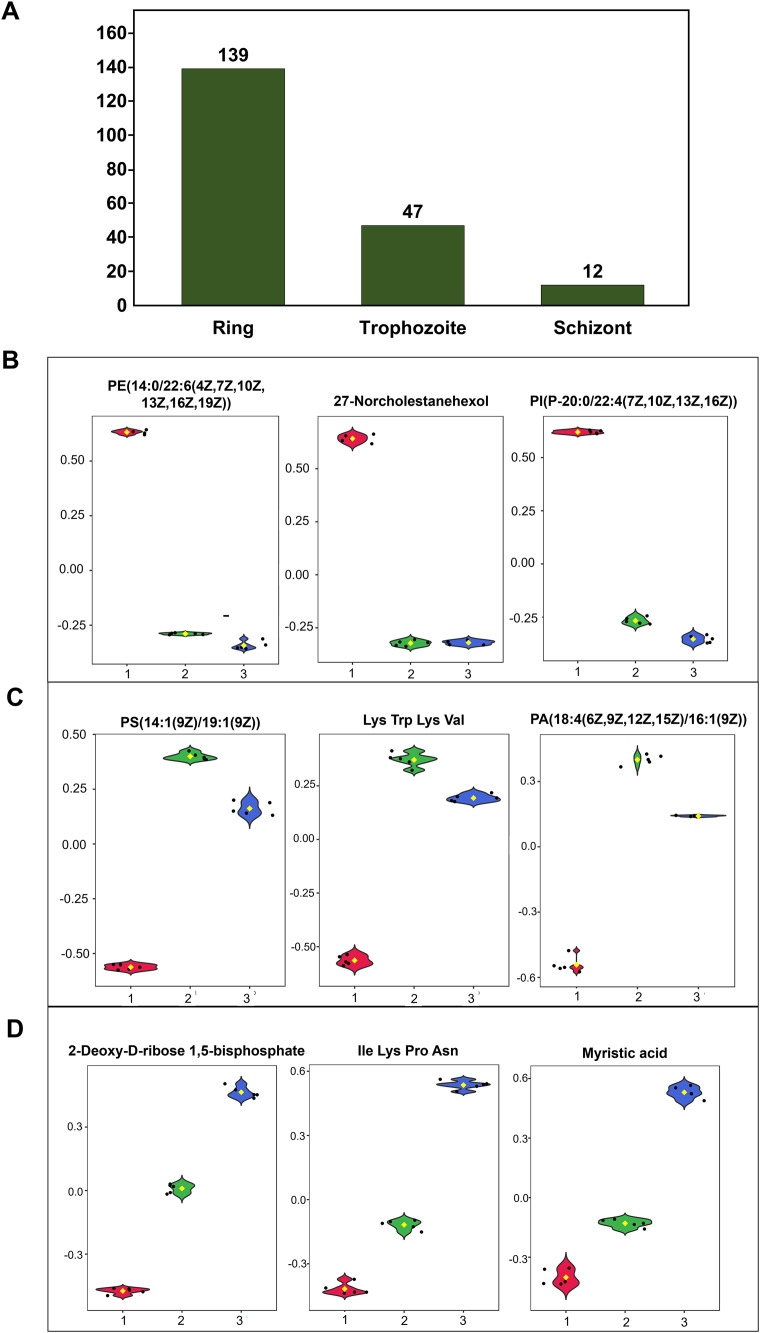
Identification of biased metabolites among three erythrocytic stages of *P. knowlesi.* (A) Summary of biased metabolites detected at each stage. Box plot of top three (B) ring-biased metabolites, (C) trophozoite-biased metabolites, (D) schizont-biased metabolites as analyzed by ANOVA using the MetaboAnalyst software (p-value < 0.01). Red, green, and blue colors represent the ring, trophozoite, and schizont, respectively.

**Fig 4:**
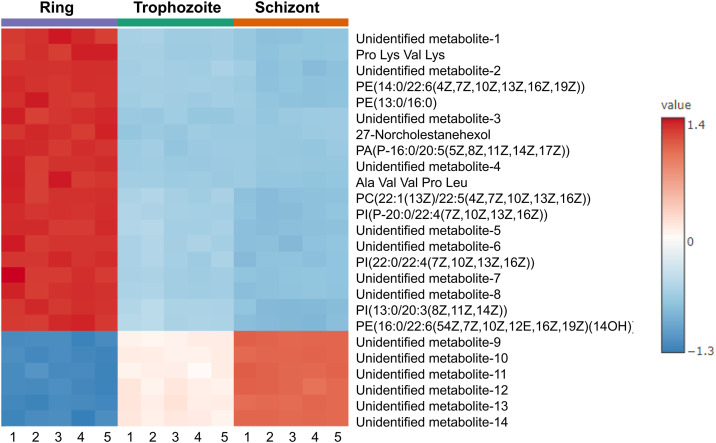
Hierarchical clustering heatmaps of the top 25 differential features from metabolomics profiles of *P. knowlesi* at ring (purple), trophozoite (green), and schizont (red) stages. Numbers 1-5 represent five biological replications of each stage. The value represents the Euclidean distance. The heatmap was generated using MetaboAnalyst software.

The relative quantification of metabolomic profiles of *P. knowlesi* ring, trophozoite, and schizont stages was compared using ANOVA analysis. After annotation, a total of 198 features were identified as stage-biased metabolites (Fig 3A and S2 Table). Five biological replications of each sample were applied in this study. Among them, the metabolomic profiles of the ring stage showed significant differences compared to those of the trophozoite and schizont stages. As a result, 139 ring-biased metabolites and 47 trophozoite-biased metabolites were detected in this study (Table 5). In the case of schizont, 12 schizont-biased metabolites were found. Most of the biased metabolites were small peptides and lipid species, which are the major components of the *Plasmodium* membrane. Box plots of the top 3 ring-biased, trophozoite-biased, and schizont-biased metabolites analyzed by ANOVA (p-value < 0.01) are shown in Figs 3B-4D. In addition, a heat map analysis was also performed to explore the correlations between the three stages of *P. knowlesi* and their metabolomic profiles (Fig 4). The heat map pattern of the ring stage was predominantly different from the trophozoite and schizont stages, corresponding to the comparison by ANOVA. According to the heatmap, 27-norcholestanehexol and some glycerophospholipids were identified as the top 25 ring-biased metabolites.

**Table 5:** Top 20 biased metabolites of three erythrocytic stages *P. knowlesi.*

Potential Metabolites		m/z	RT (min)	Mass error (ppm)	Adduct	Mode	P-value
**Ring-biased metabolites**							
1	PE(14:0/22:6(4Z,7Z,10Z,13Z,16Z,19Z))	735.4862	9.20	3	M+	Positive	3.27E-20
2	27-Norcholestanehexol	472.3612	9.43	5	M + NH_4_	Positive	1.99E-19
3	PI(P-20:0/22:4(7Z,10Z,13Z,16Z))	926.6212	9.09	4	M+	Positive	3.2E-19
4	PA(P-16:0/20:5(5Z,8Z,11Z,14Z,17Z))	661.4607	9.58	1	M + H-H_2_O	Positive	3.52E-19
5	PC(22:1(13Z)/22:5(4Z,7Z,10Z,13Z,16Z))	928.6184	9.07	1	M + K	Positive	1.42E-18
6	PI(22:0/22:4(7Z,10Z,13Z,16Z))	969.6446	9.05	1	M-H+	Positive	1.98E-18
7	Pro Lys Val Lys	488.3566	9.41	2	M + NH_4_	Positive	3.39E-18
8	PE(13:0/16:0)	649.4679	9.31	1	M+	Positive	4.5E-18
9	PE(16:0/22:6(54Z,7Z,10Z,12E,16Z,19Z)(14OH))	818.4726	9.15	1	M + K	Positive	5.88E-18
10	PI(13:0/20:3(8Z,11Z,14Z))	801.4943	9.16	3	M + H-H_2_O	Positive	7.09E-18
11	Ala Val Val Pro Leu	515.3559	9.37	1	M + NH_4_	Positive	7.22E-18
12	PA(O-16:0/22:6(4Z,7Z,10Z,13Z,16Z,19Z))	705.4865	9.55	0	M-H+	Positive	1.4E-17
13	PC(10:0/18:1(9Z))	714.4457	9.23	2	M + K	Positive	1.44E-17
14	Lys Lys Lys	444.3304	9.42	3	M + ACN + H	Positive	1.56E-17
15	PE(14:1(9Z)/P-16:0)	709.4908	9.23	3	M + ACN + H	Positive	1.59E-17
16	PE(14:0/22:6(4Z,7Z,10Z,13Z,16Z,19Z))	753.5168	9.20	1	M + NH_4_	Positive	1.89E-17
17	PG(22:1(11Z)/22:2(13Z,16Z))	929.6217	9.06	0	M + 2Na-H	Positive	1.91E-17
18	PG(O-20:0/22:4(7Z,10Z,13Z,16Z))	885.5953	9.09	0	M + 2Na-H	Positive	2.28E-17
19	PI-Cer(t18:0/20:0(2OH))	868.595	9.12	3	M-H+	Positive	4.49E-17
20	PC(20:2(11E,14E)/20:2(11E,14E))	882.5952	9.13	1	M + 2Na-H	Positive	4.66E-17
**Trophozoite-biased metabolites**							
1	PS(14:1(9Z)/19:1(9Z))	744.4818	13.08	0	M-H	Negative	8.31E-17
2	Lys Trp Lys Val	540.3285	8.58	2	M-H_2_O-H	Negative	5.82E-16
3	PA(18:4(6Z,9Z,12Z,15Z)/16:1(9Z))	665.4202	9.96	2	M-H	Negative	1.29E-15
4	PS(13:0/14:0)	664.4172	9.96	3	M-H	Negative	3.89E-15
5	PS(12:0/12:0)	622.3712	9.85	2	M-H	Negative	1.26E-14
6	PS(12:0/13:0)	618.375	10.70	3	M-H_2_O-H	Negative	1.97E-14
7	Lys Lys Arg Tyr	592.3599	10.40	4	M-H	Negative	4.7E-14
8	PE(18:0/0:0)	480.3078	10.09	4	M-H	Negative	5.07E-14
9	PG(12:0/20:4(5Z,8Z,11Z,14Z))	695.4312	11.07	3	M-H_2_O-H	Negative	1.1E-13
10	Asp Lys Arg His	553.2856	1.12	1	M-H	Negative	1.2E-13
11	PS(12:0/17:0)	692.4491	11.57	2	M-H	Negative	1.64E-13
12	His Lys Leu Val Val	636.4206	12.52	2	M + ACN + H	Positive	1.92E-13
13	PE(22:5(4Z,7Z,10Z,13Z,16Z)/22:6(4Z,7Z,10Z,13Z,16Z,19Z))	802.5206	11.93	3	M + H-2H_2_O	Positive	4.58E-13
14	PG(15:0/22:4(7Z,10Z,13Z,16Z))	765.5099	10.94	4	M-H_2_O-H	Negative	1.6E-12
15	PS(12:0/16:1(9Z))	658.4072	11.73	2	M-H_2_O-H	Negative	1.77E-12
16	Phe Phe Phe Tyr	621.2737	1.09	3	M-H	Negative	2.41E-12
17	PS(14:0/14:1(9Z))	658.407	12.17	2	M-H_2_O-H	Negative	2.47E-12
18	PS(22:4(7Z,10Z,13Z,16Z)/0:0)	554.2894	1.08	2	M-H_2_O-H	Negative	2.48E-12
19	Val Arg Trp Trp	644.3318	9.05	1	M-H	Negative	2.83E-12
20	PS(13:0/20:1(11Z))	746.4944	12.80	5	M-H	Negative	2.83E-12
**Schizont-biased metabolites**							
1	2-Deoxy-D-ribose 1,5-bisphosphate	292.9843	1.00	3	M-H	Negative	1.85E-16
2	Ile Lys Pro Asn	469.2792	14.21	2	M-H	Negative	4.43E-16
3	Myristic acid	227.2014	11.42	1	M-H	Negative	1.58E-14
4	Asp Pro Lys Lys	485.2729	11.36	0	M-H	Negative	1.78E-13
5	2-glyceryl-PGD2	425.2544	13.95	0	M-H	Negative	2.63E-13
6	Isostearic acid	283.2636	14.93	2	M-H	Negative	2.51E-12
7	Glu Leu Ile	354.2027	10.4	0	M-H_2_O-H	Negative	2.74E-12
8	PIP2(16:0/16:0)	951.4403	1.13	0	M-H_2_O-H	Negative	3.13E-12
9	TG(17:1(9Z)/18:3(9Z,12Z,15Z)/19:0)	900.8053	0.96	4	M + NH_4_	Positive	3.18E-12
10	PA(15:1(9Z)/20:3(8Z,11Z,14Z))	647.4449	8.89	0	M + H-2H_2_O	Positive	3.97E-12
11	2-Hexyldecanoic acid	255.2328	13.17	1	M-H	Negative	4.41E-12
12	Pro Arg Arg	426.2575	13.96	2	M-H	Negative	1.3E-11

*RT: Retention time.

### Pathway analysis of *P. knowlesi* stage-differential metabolites

To gain a deeper understanding of each erythrocytic stage of *P. knowlesi*, pathway analysis was conducted using statistical significance (p-value < 0.01). Based on the results, significant pathways were identified only for metabolites that increased in the ring stage and the schizont stage. No significant pathways were predicted for metabolites related to the trophozoite-biased stage, likely due to the low number of identified differential metabolites in these categories. A total of 16 potential pathways were identified from ring-biased metabolites (8 pathways) and schizont-biased metabolites (8 pathways), as shown in Table 6. The synthesis of phosphatidylserine (PS) was predominant from the ring-biased metabolite profile with a p-value of 0.00279, while G alpha (q) signaling pathways and the phosphatidylinositol 3-kinase (PI3K)- protein kinase B(Akt) signaling pathways were associated with schizont-biased metabolites with the p-value of 1.49^E-04^ and 0.00186, respectively. Overall, this finding illustrates the distinct mechanisms occurring during each erythrocytic stage of *P. knowlesi*.

**Table 6:** Pathways associated with *P. knowlesi* stage-biased metabolites.

	Potential pathways	p-value
Ring-biased metabolite	Synthesis of PS	0.00279
	Acyl chain remodeling of CL	0.00557
	Macroautophagy	0.00557
	Acyl chain remodelling of PE	0.00603
	Autophagy	0.00603
	Ion transport by P-type ATPases	0.00742
	Synthesis of glycosylphosphatidylinositol (GPI)	0.00881
	Synthesis of PE	0.00927
Schizont-biased metabolite	G alpha (q) signaling events	1.49E-04
	Phosphoinositide 3-kinase (PI3K)/Akt signaling pathway	0.00186
	PtdIns(4,5)P2 in cytokinesis pathway	0.00186
	Regulation of actin cytoskeleton	0.00232
	G-protein beta:gamma signaling	0.00325
	Intracellular Signalling Through Adenosine Receptor A2a and Adenosine	0.00418
	Transport of fatty acids	0.00464
	Angiopoietin-like protein 8 regulatory pathway	0.00464

### Identification of potential drug targets based on ring stage–biased metabolites in *P. knowlesi*

Due to essential functions, key enzymes within the metabolic pathways might effectively disrupt parasite development and viability. In this study, metabolic pathways were selected for drug target identification based on their stage-specific significance and the availability of corresponding information in publicly accessible databases. The PS metabolism and phosphatidylinositol signaling system were prioritized and used to identify the potential drug targets associated with the ring and schizont stages, respectively. The information on those pathways in *P. knowlesi* and humans was derived from the KEGG database. All *P. knowlesi* proteins belonging to each pathway were analyzed in this work. For PS metabolism, a total of six key proteins were identified in the metabolic processes associated with PS in *P. knowlesi*, including 2,3-bisphosphoglycerate-dependent phosphoglycerate mutase, serine hydroxymethyltransferase, 5-aminolevulinate synthase, aminomethyltransferase, glycine cleavage system H protein, and dihydrolipoyl dehydrogenase. In contrast, seven proteins were identified in the phosphatidylinositol signaling system of *P. knowlesi* (phosphatidylinositol 4-kinase A, phosphatidylinositol 3-kinase, CDP-diacylglycerol--inositol 3-phosphatidyltransferase, diacylglycerol kinase, calmodulin, cytidine diphosphate-diacylglycerol synthase, and inositol-hexakisphosphate 5-kinase). To evaluate the protein sequence similarity, the amino acid sequences of these proteins were compared to those of sequence databases from different *Plasmodium* species (*P. knowlesi*, *P. falciparum*, *P. vivax*, *P. malariae*, and *P. ovale*) and human (S3 Table and S1 Table in S1 Appendix). Among six proteins belonging to the PS metabolism, the 2,3-bisphosphoglycerate-dependent phosphoglycerate mutase exhibited the highest percentage of identity across the five *Plasmodium* species (>90%). In contrast, the glycine cleavage system H protein of *P. knowlesi* demonstrated relatively low sequence similarity to the human homolog (28.97%). Since the low similarity to human homologs may reduce the risk of off-target effects or host toxicity, the glycine cleavage system H protein was considered a PS metabolism-based drug target candidate. In the case of the phosphatidylinositol signaling system, calmodulin possessed the highest percentage of identity in *Plasmodium* species and human (>90%). Notably, the amino acid sequence of CDP-diacylglycerol-inositol 3-phosphatidyltransferase was not detected in humans (*Homo sapiens*; taxid:9606) as analyzed by the Blastp from the NCBI using both ClusteredNR (lcl|Query_5899511) and UniProtKB/Swiss-Prot databases (lcl|Query_6061898). Additionally, it also demonstrated the high percentage of identity across other *Plasmodium* species (>70%). Therefore, the CDP-diacylglycerol-inositol 3-phosphatidyltransferase revealed the potential as a drug target for *P. knowlesi* infection. Overall, the glycine cleavage system H protein and CDP-diacylglycerol-inositol 3-phosphatidyltransferase were promising therapeutic targets for the development of novel antimalarial agents *P. knowlesi* at the ring and schizont stages, respectively.

## Discussion

In this study, metabolomic profiles of *P. knowlesi* at three different erythrocytic stages (ring, trophozoite, and schizont) were analyzed using MS-based metabolomics. Three erythrocytic stages were cultivated *in vitro*, adapted from the method in which *P. knowlesi* was successfully cultivated in human erythrocytes [33]. Regarding the metabolomics profile, diverse lipid species, in particular glycerophospholipids, were consistently identified as the common metabolites and the most abundant metabolites across the three blood-stage samples based on peak intensities. Glycerophospholipids are the major components of the *Plasmodium* membrane, with a high abundance of PC and PE. Additionally, various phospholipids have been reported in purified *Plasmodium* parasite, including PC (40–50%), PE (35–45%), PI (4–11%), SM and PS (< 5%) [34,35]. The majority of these lipids originate from the enzymatic processes within the parasite, which are essential for the regulation of parasite development [36–38]. Since *Plasmodium* is auxotrophic for amino acids due to the lack of most amino acid biosynthetic pathways, small peptides detected in this study may be obtained through the digestion of host erythrocyte hemoglobin during their blood stages [39,40]. Nevertheless, some absent and rare amino acids in human hemoglobin (isoleucine and methionine) might be acquired from other extracellular sources [41]. Regarding some differences on the list of top 20 most abundant metabolites, a variety of acids were detected in the trophozoite and schizont stages. These acids might be obtained from various metabolic pathways of *Plasmodium* parasites such as fatty acid biosynthesis and tricarboxylic acid (TCA) cycle, which the letter attributes many functions during the erythrocytic stages [42]. In a previous report, the ring stage possessed glycolysis at a significantly reduced rate in comparison to the trophozoite stage [43]. Since glycolysis provides pyruvate, a key substrate for the TCA cycle [44], a higher level of glycolysis in the trophozoite and schizont stages might increase acid levels through the TCA cycle.

Both glycerophospholipid metabolism and sphingolipid *de novo* biosynthesis are the most significantly associated pathways to the most common metabolites in the three blood stages of *P. knowlesi*, since it is the major components of the *Plasmodium* membrane as mentioned above [34,35]. After comparing the glycerophospholipid metabolic pathway in humans and *P. knowlesi*, phosphoethanolamine N-methyltransferase (PMT) was considered a drug target candidate due to its absence in humans. In biochemical processes, PMT is an enzyme that plays essential roles in phosphatidylcholine biosynthesis by catalyzing the conversion of phosphoethanolamine to phosphocholine through a series of methylation steps. In general, it has been identified in a diverse range of organisms, such as nematodes, plants, and the malaria parasite [45,46]. Since PMTs are conserved across various human-infecting *Plasmodium* spp, but are not found in mammals, they indicate promising candidates for the development of antimalarial drugs due to the potential cross-reactivity against other *Plasmodium* species as well as their low risk of host toxicity. In a previous study, PMT was also used in the drug development research to identify drugs with potent antimalarial activity [47], and it was also reported to be a potential biomarker for the diagnosis of two *Plasmodium* species (*P. falciparum* and *P. knowlesi*) [48]. However, *in silico* knock-out of PMT did not completely eliminate PC production from serine in *P. knowlesi*, indicating that while PMT may be a promising drug target in *P. falciparum*, it might not be effective in *P. knowlesi* [35].

Investigating stage-specific metabolites across the erythrocytic cycle can offer biochemical information into the biology and underlying mechanisms of *P. knowlesi*. In this study, the overview of metabolomic profiles obtained from *P. knowlesi* stages was significantly different as analyzed by PCA and PLS-DA. The result agreed with a previous study [49] that found the proteomic profile of the ring stage differs from that of the trophozoite and schizont stages. Regarding the stage-biased metabolites, the altered metabolites were predominantly observed in the ring stage, with approximately 70% of the total identified differential metabolites. This result indicates that the ring stage-metabolomic profile exhibited high differences in comparison to the trophozoite and schizont stages. This finding might support the observation that the morphology of the ring stage differs from that of the trophozoite and schizont stages, as detected by blood smear microscopy [50,51]. In previous studies, omics technologies ranging from genomics to metabolomics can be used to study the developmental stages of *Plasmodium* [52]. As an immature form, ring-specific metabolisms for development were expected to differ from those of the mature stages (trophozoite and schizont). For example, only trophozoites and schizonts actively produce ɣ-aminobutyric acid (GABA) during asexual development [53]. Additionally, the noticeable transitional phase from the ring to the trophozoite stage was previously observed, whereas the parasites grew steadily in size without significant morphological alterations throughout the development of the trophozoite towards the schizont stage [54]. Pathway analysis was further conducted to enhance understanding of the biased metabolic pathways across three blood stages of *P. knowlesi*. As a pathway associated with ring-biased metabolites, the synthesis of phosphatidylserine (PS) was expected to be involved with the development of the immature parasite (ring form) to the mature stages. In asexual blood stages and gametocytes, *Plasmodium* parasites synthesize approximately 300 diverse lipid species, which play crucial roles in facilitating growth, reproduction, transmission, and proliferation [55]. Among them, PS is one of the major components that characterize *Plasmodium* membranes [56]. Additionally, PS has been involved in the establishment of erythrocyte cytoadhesion by *P. falciparum*, which infected cells displayed PS on their surface as intracellular parasites to trophozoite and schizont stages [57]. For the schizont stage, the increased metabolites were related to the G alpha (q) signaling and PI3K-Akt signaling pathway, the latter has been reported to be potentially involved with the Artemisinin resistance in *P. falciparum* [58]. Overall, the metabolomic profile of *P. knowlesi* during the ring stage predominantly differed from that of the trophozoite and schizont stages, presumably attributable to variations in their morphology and various metabolisms. This result corresponded with previous work showing that metabolic profiling of *P. falciparum*-infected red blood cells at the ring stage revealed mostly no differences compared to uninfected red blood cells, but the significant changes were detected at the trophozoite and schizont stages of infected red blood cells [59]. However, the previous study primarily focused on analyzing extracellular metabolites present in the culture supernatant, which differs significantly from the analysis of intracellular metabolites conducted in this study. This distinction in sample type may contribute to differences in the observed metabolic profiles and interpretations.

To explore potential alternative drug targets, this study focused on the synthesis of PS as a ring–associated pathway since the ring form is present at the initial phase of the blood stage of *P. knowlesi*, in which the inhibition could effectively disrupt the parasite progression. In general, L-serine serves as a biosynthetic precursor for several compounds, including PS. Therefore, the glycine, serine, and threonine metabolism of *P. knowlesi* from the KEGG database was selected for the drug target identification. Among six proteins belonging to the glycine, serine, and threonine metabolism of *P. knowlesi*, 2,3-bisphosphoglycerate-dependent phosphoglycerate mutase is an important enzyme in the glycolysis and gluconeogenesis pathways by catalyzing the interconversion of 3-phosphoglycerate and 2-phosphoglycerate [60,61]. While glycine hydroxymethyltransferase is the enzyme that converts glycine into serine [62], 5-aminolevulinate synthase catalyzes the conversion of glycine and succinyl-CoA to 5-aminolevulinate [63]. Besides those four candidates, there were two proteins belonging to the glycine cleavage system as drug targets, H protein and aminomethyltransferase (T-protein), the latter catalyzes the transfer of a methylene group from H-protein to tetrahydrofolate, forming N5, and N10-methylene THF (CH2-THF) [63]. The H-protein is part of the glycine cleavage system, which is essential for cellular function through its role in energy and amino acid metabolism. H-protein interacts with the L-protein (E3 subunit) to reoxidize dihydrolipoamide using NAD⁺ as the final electron acceptor [64]. Since the glycine cleavage system H protein revealed the lowest amino acid sequence similarity to the human database, it was considered a promising drug target for *Plasmodium* infection, particularly during the ring stage. While multiple studies support the essential role of H-protein in *P. falciparum*, there is currently no direct evidence demonstrating its essentiality in *P. knowlesi*. Consequently, H-protein might be a promising drug target in *P. falciparum*, but it may not be effective in *P. knowlesi*.

Besides the ring stage, the potential drug target based on the schizont-biased metabolites was also analyzed. According to the KEGG database, the PI3K-Akt was associated with the phosphatidylinositol signaling system in *P. knowlesi*. Thus, the phosphatidylinositol signaling system was used for the identification of potential drug targets. As a result, the CDP-diacylglycerol inositol-3-phosphatidyltransferase was selected as a drug target due to its high percentage of identity across other *Plasmodium* species. Importantly, it was not detected in the human database during the protein sequence alignment using Blastp (NCBI). The CDP-diacylglycerol inositol-3-phosphatidyltransferase is an enzyme involved in the biosynthesis of phosphatidylinositol, and it is essential for the biosynthesis of cell membranes and cell growth [65]. This enzyme catalyzes the formation of phosphatidylinositol (PI), a precursor for glycosylphosphatidylinositol (GPI) anchors and phosphoinositides, which are vital for membrane structure and signaling in eukaryotic cells. In *Trypanosoma brucei*, the causative agent of African sleeping sickness, PI synthesis is crucial for survival. A study demonstrated that conditional knockout of the PI synthase gene in bloodstream forms led to a significant decrease in PI levels, impaired GPI biosynthesis, and ultimately parasite death, confirming the enzyme’s essentiality [66]. CDP-diacylglycerol inositol-3-phosphatidyltransferase plays a critical role in parasite survival by facilitating essential lipid biosynthesis pathways, making it a promising target for the development of antiparasitic therapies. Although several key findings were made, further confirmation still remains important. The essentiality of a potential drug target in the parasite can be validated through gene knockdown approaches, the use of specific inhibitors, or target-specific antibodies to suppress its expression or inhibit its activity, followed by assessment of parasite viability.

One potential limitation of this study is that preparation of ring-stage parasites, which co-purified with uninfected red blood cells, could lead to a higher degree of red cell ghost contamination relative to trophozoite and schizont samples. Such contamination could influence the quantification and interpretation of metabolites typically abundant in red cells. Nonetheless, we consider it unlikely that this substantially affects our conclusions, as sphingomyelin – one of the most abundant red cell membrane lipids – was detected at very low levels among the ring-abundant metabolites (S2 Table). Another potential source of artefact is the use of horse serum in parasite culture, which may introduce non-physiological conditions. Horse serum was selected because it consistently supports robust parasite growth with a replication cycle of 24 hours, comparable to that observed in vivo. Thus, while some artefacts cannot be excluded, we believe the overall parasite biology is still well preserved under these conditions.

In conclusion, this work provided informative metabolomic profiles of three distinct blood stages of *P. knowlesi* using MS-biased metabolomic technology. Additionally, CDP-diacylglycerol-inositol 3-phosphatidyltransferase was identified as a potential therapeutic target. The foundational molecular knowledge and mechanisms provided in this study may contribute to improvement in malaria research, particularly in the areas of drug discovery and diagnostic development.

## Supporting information

S1 Appendix The protein sequences obtained from the NCBI database. (ZIP)
