# Supplementary material for: Mass spectrometry-based metabolomics uncovers distinct metabolic signatures and potential therapeutic targets in Plasmodium knowlesi
Source: PLoS One. 2025 Nov 13;20(11):e0337058. doi: 10.1371/journal.pone.0337058 (PMC12614518; doi:10.1371/journal.pone.0337058)
Supplement: S1 Appendix — (ZIP) [file pone.0337058.s001.zip › Supplementary information/S1 Appendix.docx]

**Supporting information**

**S1 Appendix: The protein sequences obtained from the NCBI database**

**1. Phosphoethanolamine N-methyltransferase**

>4IV8_A Chain A, Phosphoethanolamine N-methyltransferase,putative [Plasmodium knowlesi strain H]

MGSSHHHHHHSSGLVPRGSHMVSESVDIEYLENNQYSDEGIKAYEFIFGEDYISSGGIVATTKILSDIYLEPNSKVLDIGSGLGGGCKYINEKYDAHVYGVDICEKMIAIAKLRNKDKSKVEFEAMDILKKDFPECTFDMIYSRDAILHLPYADKKKLFEKCYKWLKPNGILLITDYCADKIENWDEEFKAYINKRKYTLIPIQDYGDLIKSCNFQNVQAKDISDYWLELLQMELNKLEEKKDEFLKLYPTDEYNSLKDGWTRKIKDTKRHLQKWGYFKAQKMV

>XP_001350151.1 phosphoethanolamine N-methyltransferase [Plasmodium falciparum 3D7]

MTLIENLNSDKTFLENNQYTDEGVKVYEFIFGENYISSGGLEATKKILSDIELNENSKVLDIGSGLGGGCMYINEKYGAHTHGIDICSNIVNMANERVSGNNKIIFEANDILTKEFPENNFDLIYSRDAILHLSLENKNKLFQKCYKWLKPTGTLLITDYCATEKENWDDEFKEYVKQRKYTLITVEEYADILTACNFKNVVSKDLSDYWNQLLEVEHKYLHENKEEFLKLFSEKKFISLDDGWSRKIKDSKRKMQRWGYFKATKN

>XP_001614208.1 phosphoethanolamine N-methyltransferase, putative [Plasmodium vivax]

MISEPVDIKYLENNQYSDEGIKAYEFIFGEDYISSGGIIATTKILSDIQLDANSKVLDIGSGLGGGCKYINEKYGAHVHGVDICEKMVTIAKLRNQDKAKIEFEAKDILKKDFPESTFDMIYSRDSILHLSYADKKMLFEKCYKWLKPNGILLITDYCADKIENWDEEFKAYIKKRKYTLMPIQEYGDLIKSCKFQNVEAKDISDYWLELLQLELSKLEEKKEEFLKVYSIKEYNSLKDGWTRKIKDTKRDLQKWGYFKAQKMI

>XP_028862913.1 phosphoethanolamine N-methyltransferase, putative [Plasmodium malariae]

MNNQPVDKKYLENNQYSDEGIKSYEFIFGEDYISSGGISATTKILSDIHLDENSKVLDIGSGLGGGCKYINNKYGSYVYGVDICENLVSIARTRNKEKPKIEFEAIDILKKEFPLNHFDMIYSRDSILHLSVEDKKKLFEKCYLWLKPNGVLLITDYCADKEENWDEEFKEYVKKRKYALITTEEYGQLLSFCNFKNVEAKNISDYWLQLLEMEVNRLEQKKEEFTSKYSTKEYESLINGWNRKIRDTKRNLQVWGYFKAYKI

>SBS85886.1 phosphoethanolamine N-methyltransferase [Plasmodium ovale curtisi]

MSNESVDKKYLESYQYSDESIKFYEFIFGEDYISSGGLTATIKILSDIQLDENSRVLDIGSGLGGGCKYINEKYGSYVYGVDICEKTVSIAKMRNKGKTKIEFDSCDILKKEFKENSFDMIYSRDSILHLTVPDKKRLFEKCYVWLKPNGILLITDYCADKKDNWDTEFKEYITLRKYDLTTIDEYGNLISSCNFKDVQATDISDYWLELLEMELHRLNEKKEEFLSEYSLKDYNTLKDGWVRKIRDTKRNLQKWGLFKAHKK

**2. 2,3-bisphosphoglycerate-dependent phosphoglycerate mutase [EC:5.4.2.11]; Phosphoglycerate mutase, putative**

>XP_002259202.1 phosphoglycerate mutase, putative [Plasmodium knowlesi strain H]

MTTYTLVLLRHGESTWNKENKFTGWTDVPLSEQGEKEAISAGNYLKEKNFRFDVVYTSVLKRAITTTWNVLKTGELLHVPVIKTWRLNERHYGSLQGLNKSETAKKYGEEQVKIWRRSYDIPPPKLDKEDSRWPGHNVVYKNIPKDVLPFTECLKDTVERVLPLWFDHIAPDILANKKVLVSAHGNSLRALVKHLDKLTEADVLELNIPTGVPLVYELDENLKPIKHYYLMDSEELKKKMDEVANQGKAK

>XP_001347879.1 phosphoglycerate mutase, putative [Plasmodium falciparum 3D7]

MTTYTLVLLRHGESTWNKENKFTGWTDVPLSEKGEEEAIAAGKYLKEKNFKFDVVYTSVLKRAICTAWNVLKTADLLHVPVVKTWRLNERHYGSLQGLNKSETAKKYGEEQVKIWRRSYDIPPPKLDKEDNRWPGHNVVYKNVPKDALPFTECLKDTVERVLPFWFDHIAPDILANKKVMVAAHGNSLRGLVKHLDNLSEADVLELNIPTGVPLVYELDENLKPIKHYYLLDSEELKKKMDEVANQGKAK

>XP_001615319.1 2,3-bisphosphoglycerate-dependent phosphoglycerate mutase, putative [Plasmodium vivax]

MTTYTLVLLRHGESTWNKENKFTGWTDVPLSEQGEQEAIAAGNYLKEKNFRFDVVYTSVLKRAITTTWNVLKTGDMLHVPVIKTWRLNERHYGSLQGLNKSETAKKYGEEQVKIWRRSYDIPPPKLDKEDSRWPGHNVVYKNVPKDTLPFTECLKDTVERVLPFWFDHIAPDILANKKVLVSAHGNSLRGLVKHLDSLTEADVLELNIPTGVPLVYELDENLKPIKHYYLLDSEELKKKMDEVANQGKAK

>XP_028861617.1 phosphoglycerate mutase, putative [Plasmodium malariae]

MTTYTLVLLRHGESTWNKENKFTGWTDVPLSEEGEQEAVAAGKYLKEKNFQFDVVYTSVLKRAITTAWKVLKTSDLLHVPVIKTWRLNERHYGSLQGLNKSETAKKYGEEQVKIWRRSYDIPPPKLDKEDSRWPGHNAVYKNVPKDALPFTECLKDTVERVLPFWFDHIAPDILSNKKVLVAAHGNSLRGLVKHLDNMSEAEVLELNIPTGVPLVYELDENLKPIKHYYLLDSEALKKKMDEVANQGKAK

>SBT35219.1 phosphoglycerate mutase, putative [Plasmodium ovale wallikeri]

MTTYTLVLLRHGESTWNKENKFTGWTDVPLSEKGEQEAIAAGNYLKEKNFRFDVVYTSVLKRAICTTWNVLKTADLLHVPVIKTWRLNERHYGSLQGLNKSETAKKYGEEQVKIWRRSYDIPPPKLDKEDSRWPGHNVVYKNIPKDALPFTECLKDTVERVLPFWFDHIAPDILANKKVLVSAHGNSLRGLVKHLDSLTEADVLELNIPTGVPLVYELDENLKPIKHYYLLDSEELKKKMDEVANQGKAK

>NP_002620.1 phosphoglycerate mutase 1 isoform 1 [Homo sapiens]

MAAYKLVLIRHGESAWNLENRFSGWYDADLSPAGHEEAKRGGQALRDAGYEFDICFTSVQKRAIRTLWTVLDAIDQMWLPVVRTWRLNERHYGGLTGLNKAETAAKHGEAQVKIWRRSYDVPPPPMEPDHPFYSNISKDRRYADLTEDQLPSCESLKDTIARALPFWNEEIVPQIKEGKRVLIAAHGNSLRGIVKHLEGLSEEAIMELNLPTGIPIVYELDKNLKPIKPMQFLGDEETVRKAMEAVAAQGKAKK

**3. Glycine hydroxymethyltransferase [EC:2.1.2.1]; Serine hydroxymethyltransferase, putative**

>XP_002262442.1 serine hydroxymethyltransferase, putative [Plasmodium knowlesi strain H]

MFNNQPLEQVDKELYDILADEGKRQKETINLIASENLTNLAVRECLGNRVSNKYSEGYPKKRYYGGNDYIDKIEELCQKRALEAFNVSEEEWGVNVQPLSGSAANVQALYALVGVKGKIMGMHLCSGGHLTHGFFDEKKKVSITSDMFESKLYKCNDQGYVDLDAVREMALSFKPKVIICGYTSYPRDIEYQRFRQICDEVGAYLFADISHISSFVACGILNNPFLYADVVTTTTHKILRGPRSALIFYNKKKNPGIDQKINSAVFPSFQGGPHNNKIAAVACQLKEVKSSEFKAYTEQVLLNSKALAKSLISKNIDLVTNGTDNHLIVVDLRKHGITGSKLQETCNAINIALNKNTIPSDVDCVSPSGVRIGTPAMTTRGAKEKDMEFIADILDKAIKITVNLQEQYGKKLVDFKKGLPTSVELQKLKQEVVTWAGALPFP

>4O6Z_A Chain A, Serine hydroxymethyltransferase [Plasmodium falciparum 3D7]

MRGSHHHHHHGMASMTGGQQMGRDLYDDDDKDHPFTPGMFNNDPLQKYDKELFDLLEKEKNRQIETINLIASENLTNTAVRECLGDRISNKYSEGYPHKRYYGGNDYVDKIEELCYKRALEAFNVSEEEWGVNVQPLSGSAANVQALYALVGVKGKIMGMHLCSGGHLTHGFFDEKKKVSITSDLFESKLYKCNSEGYVDMESVRNLALSFQPKVIICGYTSYPRDIDYKGFREICDEVNAYLFADISHISSFVACNLLNNPFTYADVVTTTTHKILRGPRSALIFFNKKRNPGIDQKINSSVFPSFQGGPHNNKIAAVACQLKEVNTPEFKEYTKQVLLNSKALAECLLKRNLDLVTNGTDNHLIVVDLRKYNITGSKLQETCNAINIALNKNTIPSDVDCVSPSGIRIGTPALTTRGCKEKDMEFIADMLLKAILLTDELQQKYGKKLVDFKKGLVNNPKIDELKKEVVQWAKNLPFA

>CAI7723696.1 serine hydroxymethyltransferase [Plasmodium vivax]

MFNNEPLEQIDKELHDILADEEKRQRETINLIASENLTNGAVRECLGNRVSNKYSEGYPKKRYYGGNDFIDKIEELCQKRALEAFNVSDEEWGVNVQPLSGSAANVQALYALVGVKGKIMGMHLCSGGHLTHGFFDEKKKVSITSDMFESKLYKCNSQGYVDLDAVREMALSFKPKVIICGYTSYPRDIDYQQFRQICDEVNAYLFADISHISSFVACNILNNPFLYADVVTTTTHKILRGPRSALIFFNKKRNPGIEQKINSAVFPSFQGGPHNNKIAAVACQLKEVHSPAFKEYTQQVLLNSKALAKALISKQIDLVTNGTDNHLIVVDLRKFSITGSKLQETCNAINVSLNKNTIPSDVDCVSPSGVRIGTPAMTTRGAKEKDMEFIADVLARAIKITVDLQEQYGKKLVDFKKGLPGNAQLQQLKQEVVTWAGALPFP

>XP_028864591.1 serine hydroxymethyltransferase [Plasmodium malariae]

MFNNDPLESLDKELYDILVDEEKRQRETINLIASENLMNNGVRECLGNRVSNKYSEGYPRKRYYGGNDYIDKIEELCCKR

ALEAFNVSDEEWGVNVQPLSGSAANVQALYALVGVKGKIMGMHLCSGGHLTHGFFDEKKKVSITSDMFESKLYKCNSEGYVDFDNVHEMALSFKPKVIICGYTSYPRDIDYKKFRAICDEVGAYLLADISHISSFVACNLLNNPFIYADVVTTTTHKILRGPRSALIFFNKKKNPGLDQKINSAVFPSFQGGPHNNKIAAVACQLKEVHSDFFKEYTKQVLLNSKALAQYLINNNIDLVTNGTDNHLIVVDLRKYGITGSKLQETCNSINVALNKNTIPSDVDCVSPSGIRIGTPAMTTRGAKEKDMKFIADILARAIKITVDLQQQFGKKLVDFKKGLTNNADIDQLKKEVVQWAGNLPFP

>SCQ17167.1 serine hydroxymethyltransferase, putative [Plasmodium ovale]

MFNNEPLESIDKELFDILADEEKRQRETINLIASENLTNVAVRECLGNRVSNKYSEGYPRKRYYGGNDYIDKIEELCCKRALEAFNVSEEEWGVNVQPLSGSAANVQALYALVGVKGKIMGMHLCSGGHLTHGFFDEKKKVSITSDMFESKLYKCNSEGYVDLEAVRDMAISFQPKVIICGYTSYPRDINYKRFREICDEVNAYLMADISHISSFIACNLLNNPFLYADVVTTTTHKILRGPRSALIFFNKKRNPQIEQKINSSVFPSFQGGPHNNKIAAVACQLKEVHSDFFKEYTKQVLLNSKALAQCLIDNNIDLVTNGTDNHLIVLDLRKYGITGSKLQETCNAINIAINKNTIPSDVDCVSPSGARLGTPAMTTRGAKEKDMQFIADILLKAIKITVDIQQQFGKKLIDFKKGLHNNPHLDELKAEVIQWAGKLPFP

>1BJ4_A Chain A, Serine hydroxymethyltransferase, cytosolic [Homo sapiens]

DADLWSSHDKMLAQPLKDSDVEVYNIIKKESNRQRVGLELIASENFASRAVLEALGSCLNNKYSEGYPGQRYYGGTEFIDELETLCQKRALQAYKLDPQCWGVNVQPYSGSPANFAVYTALVEPHGRIMGLDLPDGGHLTHGFMTDKKKISATSIFFESMPYKVNPDTGYINYDQLEENARLFHPKLIIAGTSCYSRNLEYARLRKIADENGAYLMADMAHISGLVAAGVVPSPFEHCHVVTTTTHKTLRGCRAGMIFYRKGVKSVDPKTGKEILYNLESLINSAVFPGLQGGPHNHAIAGVAVALKQAMTLEFKVYQHQVVANCRALSEALTELGYKIVTGGSDNHLILVDLRSKGTDGGRAEKVLEACSIACNKNTCPGDRSALRPSGLRLGTPALTSRGLLEKDFQKVAHFIHRGIELTLQIQSDTGVRATLKEFKERLAGDKYQAAVQALREEVESFASLFPLPGL

**4. 5-aminolevulinate synthase [EC:2.3.1.37]; delta-aminolevulinic acid synthetase, putative**

>XP_002262533.1 delta-aminolevulinic acid synthetase, putative [Plasmodium knowlesi strain H]

MRQKKTLKVSIHEIKKYCPFVKNIQLFYNANESKSKDKHNKNNVVLSVMSNLCPVGKAINEKRLIIIDNKSKINIFKILRKSNIISRHLVDSLAARSRTLEVKGQQEHNQRGEAPTVGRTPPDEYSLTGDVCRTQNTHSGECLCKSWGKKTSLHSNTLSVVENKSNVSLTWSNEAYNLFQKECTNDLNILLNKLHLDKRYRVFTILNKCRESYPNVYVENGKLLLPFFFAFFQNFGYKHCVGNSACRIGGNYDGVADGLRDSTRRKENTNSWDGCNVRTSLMKRTNEKTVVWCSNDYLCLSNNEQVIDVGIETLKKIGNSSGGTRNISGSLLNHSHLEYILAKWFNKESALLFTSGYIANVGALETLGKLLNLVFVSDEMNHASIINGIRESRCEKIIFKHNDMVDLERVLKKIRTDKESKNRKIMIVFESIYSMSGNISNIPCIVQLAKKYNALTYVDEVHAVGLYGKTGSGYLEELNLCDHIDIINGTLSKAIGSLGGFICANKYFIDVIRSYSPHFIFTTSLTPVNINTSAEAIHIIQTDRNLRNKFRKVVQKTKERLEQQGIHILKNNSHIVVALINSAEKCKQICDDLLTEYNIYLQPINYPTVPRGSERIRITPSPYHTDEHIEKLAYSLYMLFKKYQVNMFDGENSQAQMEL

>ETW55862.1 5-aminolevulinic acid synthase [Plasmodium falciparum Palo Alto/Uganda]

MRKKRTLKVSINEIKKYCPFVKNIQFLYNTNEKKNNLVLSVMSDLCPVGKAINEKHFIIIDNKSKINIIKILKQANMQSKVLVQCIKNKNIEKENMSNDDLLKSGKRNNNVLFYDILEKNKNDHSFQINDNTIQKNNIIYKYINSLDEYKLFKNNCNNNLKDLLNKLYTDKRYRIFTILNKYRINYPNVYIENNKLMLPSFYEFYQKYGYKPCIGNIRYQLSASFEDNNKNICSFSHKNKENYLFNFWNLHIDNVSNEKTVVWCSNDYLCLSNNEKIIEVGIETLKKIGNSSGGTRNISGSLLNHTHLEYIIAKWYNKESSLLFTSGYIANVGALETLGKLLNLIYISDEMNHASIINGIRESRCEKFIFKHNDMNDLERILYNLRINKQYENRKIMIVFESIYSMSGHISNIEYIVQLAKKYNALTYVDEVHAVGLYGNKGSGYLEELHLCNHIDIINGTLSKAIGSLGGFICANKYYIDVIRSYSSHFIFTTSLTPVNINTSAEAIHIIQNDMSLRKKLTQVVNKTKQELQERGIQVLHNNSHIVVLMINSAEKCKQICDDLLKEYNIYIQPINYPTVPMGMERIRITPSPFHTDEQIFKLVYSLYTLFKKYQVNMFDKKNKHTLMKL

>KMZ77543.1 5-aminolevulinic acid synthase [Plasmodium vivax India VII]

MRQKKTLKVSIHEIKKYCPFVKNIQLFYSANESKSKDKQNKNNVVLSVMANLCPVGKAINEKRLIIIDNKSKINILKILRKSNIISRQLVDSLGGRTDTGESIPTGEYNPTGASIPTGEYNTRGASIPRGDAHPCGECLCKSWTPTQTHTNTLSVVENKPSGSLTWSSEAYRLFQTECQNDLKELLNKLFLDRRYRVFTILNKCRKYYPNVSIENDKLFLPIFFKFFQNFGYKQCVGSSACRIGVDYDAVANGFRSATPRKETTHGWKSSNVLSSVIRTTNDKTVVWCSNDYLCLSNNQQVIDVGIETLKKIGNSSGGTRNISGSLLNHSHLEYILAKWFNKESALLFTSGYIANVGALETLGKLLNLVFVSDQMNHASIINGIRESRCEKIIFKHNDMVDLEKVLKSLRTDKEYQNRKIMIVFESIYSMSGNISNIPRIVQLAKKYNALTYVDEVHAVGLYGKTGSGYSEELNLCDHIDIINGTLSKAIGSLGGFICANKYYIDVIRSYSPHFIFTTSLTPVNINTSAEAIHIIQSDLTLRNKFRQVVQKTKESLERRGIHIMKNNSHIVVALINCAEKCKQICDDLLAEHNIYLQPINYPTVSRGSERIRITPSPYHTDEHIEKLARSLYLLFKKYQVNMFDGKNSQVQMKL

>SBT81128.1 delta-aminolevulinic acid synthetase, putative [Plasmodium malariae]

MRKEKTLKVSIDEIKKYCPFVKQIHLFYKSNDNKNNVVLSVMSNLCPIGKAINEKHIIIIDNKSKINLFKILRGSNIGLRHLVETCIKKNDTIDNVDNSIIGNYKKSGRREEKGCSEFAHNNEGGKDSNIPAYELIMKNNFSKTLTRSDKSYETFQKECSNELKGLINNLFLDKRYRVFNIINKCRKYYPSVYIENNRLLLPIFYEFYQRYGYMPCIGNAAYKVVGSPECSSIRGRDIGSSDIGSSDIGSSDIGSSDIGSSGIGSSDIGSSDIGSSDIGSSGIGSSDIGSSGIGSSSIGSSCKPSDERRGHQDLSERNERYLCNFLNVHKGRVTNEKTVVWCSNDYLCLSNNEKIIDVGIETLKKIGNSSGGTRNISGSLVNHTHLEYILARWFNKESALLFTSGYVANVGALGTLGKLLNLIYVSDEMNHASIINGIKESKCEKYIFKHNDMVHLEKILKNLRMENEYANRKIMIVFESIYSMSGHISPIPQIVHLAKKYNALTYVDEVHAVGLYGQKGSGYLEELNMCDHIDIINGTLSKAIGSLGGFICANKFYIDVIRSYCSHFIFTTSLTPVNINTSAEAIHIIQNDRNLRNKFRQVVKKTKEKLIERGIHIIDNKSHIVVSLINSAEKCKQICDDLLSEYNIYIQPINYPTVPKGSERIRITPSPYHTDEHISKLSDSLYTLFKRHEVNMFDMKIKLNEVKL

>SBT50269.1 delta-aminolevulinic acid synthetase (ALAS) [Plasmodium ovale wallikeri]

MRKKKTLKVSIQEIKKYCPFVKNIQLFCNNNDSKNNVVLSVMSNLCPIGKAINEKHLIVIDNKSKINIFKILRGSNVNSRNLMESFFKKCDTCDDACDACDNTTKPVQKSIEMNRKSFTCTQCEENAIGVINKHMDLNENRHDICVNNFEKPKDAYILFQDECNNDLKRHINNLFIDKRYRIFTVLNKLRKNYPNVYVENNTFFLSSFYEKYQQYGYKPCIGKGAYIIKNNFDDAFRNTNCKIRDITSFRSLTSCNGLNKSNIRILTKGEKEESNLYNWFDGNITNEKTVVWCSNDYLCLSNNEEVIDVGIETLKKIGNSSGGTRNISGSLLNHTHLEYILAKWFNKESALLFTSGYIANVGALETLGKLLNLIFVSDEMNHASIINGIKESRCEKHIFKHNDMVDLENLLKKLRGMKEYKNRKIMIVFESVYSMSGNISNIAHIVKLAKKYNALTYVDEVHAVGLYGKTGSGYAEELNLCDEIDIINGTLSKAIGSLGGFICANKYYIDVIRSYSSHFIFTTSLTPVNINTSAQAIHIIQNNLHLRNKFRDVVKKTKEELTKRGILIMENHSHIVVTLINSAEKCKQICDDLLTQYNIYIQPINYPTVPKGSERIRITPSPFHTDEHISKLADSLYVLFKKYQVNMFDKEKNKIAMAL

>5QQQ_A Chain A, 5-aminolevulinate synthase, erythroid-specific, mitochondrial [Homo sapiens]

MGHHHHHHSSGVDLGTENLYFQSMFSYDQFFRDKIMEKKQDHTYRVFKTVNRWADAYPFAQHFSEASVASKDVSVWCSNDYLGMSRHPQVLQATQETLQRHGVGAGGTRNISGTSKFHVELEQELAELHQKDSALLFSSCFVANDSTLFTLAKILPGCEIYSDAGNHASMIQGIRNSGAAKFVFRHNDPDHLKKLLEKSNPKIPKIVAFETVHSMDGAICPLEELCDVSHQYGALTFVDEVHAVGLYGSRGAGIGERDGIMHKIDIISGTLGKAFGCVGGYIASTRDLVDMVRSYAAGFIFTTSLPPMVLSGALESVRLLKGEEGQALRRAHQRNVKHMRQLLMDRGLPVIPCPSHIIPIRVGNAALNSKLCDLLLSKHGIYVQAINYPTVPRGEELLRLAPSPHHSPQMMEDFVEKLLLAWTAVGLPLQDVSVAACNFCRRPVHFELMSEWERSYFGNMGPQYVTTYA

**5. Glycine cleavage system T protein (aminomethyltransferase) [EC:2.1.2.10]**

>XP_002261234.1 aminomethyltransferase, mitochondrial, putative [Plasmodium knowlesi strain H]

MRILFKRQKRIPGIRYFSSGNKQKEEVRKTILYDVHKKNNAIFKIHNGYYIPNEYKDYTLITSHLHTRSSCSLFDYTYRPILKISGTDKINFLEKYVGSDIKGLWENECRISLLLNEKGGIIDDIVIILRENHLLLYFNIQCKKKVFKYLNEKLLENTKLDVKIEEYNSHSSICIQGSKSANVLNEIIEDDTYLENCSFMSSNITKLNNIEGCVLNRYTCTGEDGFDILVPNNHVEKLYECILSNPLVKPGGLEVLNTLRLESGFCVYGKDINENLTPIESNYKWVLGQRRLKELDFNGAHIIMNQIKNGTTIKRVGLIMNSTIVPKENSKIYTNENAHEEIGYITSSVFSPLLQKPIAMGYIKTEHAATNNLIKVECLNKLEVAQISKMPFVPLSIYKM

>XP_001350365.1 aminomethyltransferase, mitochondrial, putative [Plasmodium falciparum 3D7]

MKNIFKYKNRLTFFRKGFSTTNKPKVEIKKTILYDSHKKNNAIFKIQHGFYLPDEYKDITLITSNLHTRTNCSLFDYTYRPILKISGEDKINFIEKYVGSDIKGLWENECRISLLLNDKGGIIDDIMIILREKYLLLYLNIQCKEKVYKYLKDKLLENGKLQVQIEEFTSHSSICIQGSKSSDVLKELIDYNNESVETNLDNCSFMSSTLTKINKIDNCILNRYTCTGEDGFDILIPNKYVNDLYNLILKNELVKPGGLAVQNTLRLESGFCEYGKDINEDITPIESNYKWSLGQRRLKELNFNGAHIIMDQIKNGTKIKRVGILINTNIVPKENTKIYSHENAEQIIGYITSSVFSPVLQKPICMGYVKSEYAHINNLIKVDCLNKLEIAQITKLPFVPLSIYKL

>XP_001616437.1 aminomethyltransferase, mitochondrial precursor, putative [Plasmodium vivax]

MKIIFKRHRRLPGVRYFSSGNKQKEEVRKTILYDVHKKNNAIFKIHSGYYLPNEYKDFTLITSHLHTRSSCSLFDYTYRPILKISGADKTHFLEKYVGSDIKGLWENECRISLLLNEKGGIVDDIVIILRENHLLLYLNIQCKDKVFSYLNEKLLENTKMDVKIEEYTSHRSICIQGSKSANVLNEIIGDDTYLENCSFMSSNVTKLNKIEGCVLNRYTCTGEDGFDILVPNKHVGELYQCILNNSLVKPGGLEVLNTLRLESGFCVYGKDINEKLTPIESNYKWVLGQRRLKELDFNGAHVIINQIKNGTTIKRVGLIMDSTIVPKENSKIYTNENAHEEIGYITSSVFSPLLQKPIAMGYIKTEHAAANNLIKVECLNKLEVAQISKMPFVPLSIYKM

>XP_028862396.1 aminomethyltransferase, mitochondrial, putative [Plasmodium malariae]

MKNILKYNKRVYLLRHFSSVNKRKVLKTILYDVHKKNNAIFKIYNGYYVPDEYKDDTLITSHLHTRSNCSLFDYTYRPILKISGVDKINFIEKYVGSDIKGLWENECRMSLLLNEKGGIIDDIIIILIENHLLLYLNIQCKDKVYTYLNEKLLENTQLDVKIEEYTSHSSICIQGSKSVNILKEMVNDDINLEDCSFMSSNIYKLNKAENCLLNRYTCTGEDGFDILIPNKHITELYEYMLTNPLVKPGGLAVQNTLRLESGFCVYGKDINENFTPIESNYKWVLGQRRLKELNFNGAHIIMNQIKNGTKIKRVGLVINTSTIVPKENSKIYSNEKVDEEIGFITSSVFSPLLQKPIAMGYVKTDQSNVNNNIKVECLNKLEIAQITKMPFVPLSIYKI

>SBT77502.1 glycine cleavage system T protein, putative [Plasmodium ovale]

MKNIFTYKKRLPMLRYISSTSKPKVLKTILYDIHKKKNAIFKIHNGYYIPDEYKDDTLITSHLHTRSNCSLFDYTYRPILKISGSDKVHFLEKYVGSDIKGLWENECRISLLLNEKGGIIDDIIIILRENHLLMYLNIQCKDKVYTYLNKKLLDNTKLDVKIEEYTSHCSICIQGCKSTHVLNELINDELNLEDCSFMSSNVAKLNNIDNCLLNRYTCTGEDGFDILIPNKHVKDLYECILSNPLVKPGGLAVQNTLRLESGFCVYGKDINENFTPIESNYKWVLGKRRLKELNFNGAHIIMNQIQNGTNIKRVGLIMNSTIVPKEKSKIYPNEKIDEEIGFITSSCFSPLLQKPIAMGYVNTDQSTVNNVLKVECLNKLEVAQITKMPFVPLSIYKM

>BAA03512.1 glycine cleavage system T-protein [Homo sapiens]

MQRAVSVVARLGFRLQAFPPALCRPLSCAQEVLRRTPLYDFHLAHGGKMVAFAGWSLPVQYRDSHTDSHLHTRQHCSLFDVSHMLQTKILGSDRCKLMESLVVGDIAELRPNQGTLSLFTNEAGGILDDLIVTNTSEGHLYVVSNAGCWEKDLALMQDKVRELQNQGRDVGLEVLDNALLALQGPTAAQVLQAGVADDLRKLPFMTSAVMEVFGVSGCRVTRCGYTGEDGVEISVPVAGAVHLATAILKNPEVKLAGLAARDSLRLEAGLCLYGNDIDEHTTPVEGSLSWTLGKRRRAAMDFPGAKVIVPQLKGRVQRRRVGLMCEGAPMRAHSPILNMEGTKIGTVTSGCPSPSLKKNVAMGYVPCEYSRPGTMLLVEVRRKQQMAVVSKMPFVPTNYYTLK

**6. Glycine cleavage system H protein**

>XP_002259335.1 glycine cleavage system H protein, putative [Plasmodium knowlesi strain H]

MLLLKRPFLPARVAPLCAQRKVSRLITYYTKSHEYIKINDEDLTELKKKNNVKCKIGISNYGTEKLGEIVYIDISQNINEYVKKGECIATVESVKSVGDVYTPISGQIVDINNEIVDNVNLMNGNSESDGWILELLTNDVNEKEIMSFSEYKKMCEEEEQREATKIQQSERDCLEEKNKNKIFDLNDIKNIEEKTKK

>XP_001348010.1 glycine cleavage system H protein [Plasmodium falciparum 3D7]

MINIRKVLLPSCVITKSFLNNYYKRLITYYTKTHEYIKIEDGNLNNRKDMTNVKCKIGISNYGTHKLGEIVYVDVAHNINDHVKKGDCIATIESVKSVGDVYTPVSGKIININNKIIDNVNLMNEQSEIDGWIMELETNQINEKEIMNISEYEKMCEEEEQNEEKKIQQNEINCMEEKNKNKIFDINDMKNIENKGQGGK

>XP_001615443.1 hypothetical protein, conserved [Plasmodium vivax]

MLAQKMLLLKRPFLPARAAPFCAQRKVGRCITYYTKSHEYIKINDEHLTELKKKNNVKCKIGISNYGTEKLGEIVYIDISHNVNEYVKKGDCIATVESVKSVGDVYTPISGQIVDINNKIVDNVNLMNENSETDGWILELLTNEVNEKEIMSFSEYKKMCEEEEQREATKIQQSERDCLEEKNKNKIFDLNDIKSIEEKGKK

>SBT71426.1 glycine cleavage system H protein, putative [Plasmodium malariae]

MITLRRSFSYKKFSSLYGPCTFKRFVTYYTTTHEYIKINEQNLNDLKNKNNVQCKIGISSYGTEKLGEIVYIDITHNINDYIKKGDCIATIESVKSVGDVYTPISGKIVDINSKVIDNVNLMNGHSESEGWIMELLTNDINEKEIMDSTEYKKACEEEEQKEEKKMEQSEINCLEEKNKNKIFDLNDIKSIENKGKND

>SBS83859.1 glycine cleavage system H protein [Plasmodium ovale curtisi]

MLSPAVVRFSRSLLHMQGNSPYMQIVRRGFTTYYTKTHEYIRIKEGSLNELKNKSGVKCKIGISNYGTEKLGEIVYIDVTHNINDNVKKGDCIATIESVKSVGDVYTPISGKIININSELIDNINLMNENPESSGWIMELLTNDINDKEIMDISEYKKICQEEEQKEETTLKQSEINCLEEKNKNKMFDFNDVKNIENREGK

>CAG33353.1 GCSH [Homo sapiens]

MALRVVRSVRALLCTLRAVPLPAAPCPPRPWQLGVGAVRTLRTGPALLSVRKFTEKHEWVTTENGIGTVGISNFAQEALGDVVYCSLPEVWTKLNKQDEFGALESVKAASELYSPLSGEVTEINEALAENPGLVNKSCYEDGWLIKMTLSNPSELDELMSEEAYEKYIKSIED

**7. Dihydrolipoyl dehydrogenase [EC:1.8.1.4]**

>XP_002258179.1 dihydrolipoyl dehydrogenase, apicoplast, putative [Plasmodium knowlesi strain H]

MGARAGTYMWIGWLLLLVTQNGIAKHTRGGHTLHSLHEKGSSVRGGKIRSNMFIRCNGMRNGMRNGMRKGMRNGMRSGMSSSTRQTANQVEGCKSLRANGSGKEQPMKPSIEQYDVGILGCGVGGHAAAINAMERGLKVIIFTGDKDSIGGTCVNVGCIPSKSLLYATGKYRELKNLAKLYTYGIYTNAFRKSGKEDPVERNQLLADTVQIDIGKLKEYTQSVINKLKGGIENGLKKKKFCKNSEHVQVIYERGHIVEKNIIKGEKSGKEYQVKNIIIATGSTPNIPDNIEVDEKTVFTSDQAVKLEGLQKYMGIVGMGIIGIEFTDIYTALGSEVVSFDYSPQLLPLLDADVATYFERVFIKSKPMRVHLNTCIEYVRAGKGNQPVIIGHSERNDAEEDKPIQRNNKIKETRVDSCLVATGRKPNTNNMGLDDLQIQMNRGFVSVDEHLRVERKDQGVYDNIFCIGDANGKQMLAHTASHQALKVVDWIISNGKETHNNTVHSIASHNDWASKPIIYRNIPSVCYTTPELAFVGLTEKEAKKLYPPENIGTEISFYKANSKVLCENNDITFPERSKNNSYNKGKYNTVDNTSGMVKIVYLKESKEILGLFIVGSYASILIHEGVLALNLKLSVIDLAHMVHSHPTISEVLDTAFKAIAQVRTH

>AAS49639.1 dihydrolipoamide dehydrogenase [Plasmodium falciparum]

MVIRQNIKHIVKLNVVTLIWLSYLFLLKPHGTLKNMMVCNAVLLPFNEKNKGINNFVYINPKNIILNKIKKDVIKLEKDNIILCQHNRKRDNYIKQQKRKEKNANNFTFMLKGSTQNIMNINEKEYDLAIIGCGVGGHAAAINAMERNLKVIIFAGDENCIGGTCVNVGCIPSKALLYATNKYRELKNLDKLYYYGIHSNIFQNNKNTEIENNQLVSNSFQINITKLKEYTQSVIDKLRNGISHGFKTLKFNKNSEHVQVIYEHGQLLDKNTIKSKKSGNTYKVKNIIIATGSVPNIPNNVEIDDKSVFTSDMAVKLVGLKNYMSIIGMGIIGLEFADIYTALGSEITFLEYSSELLPIIDNDVAKYFERVFLKNKPVNYHLNTEVKYIKASKNNNPVIIGYSHRTGNDDNEKKNMTDVKELYVDSCLVATGRKPNTQNLGLEKLKIQMNRGYVSVNDNLQVKMENNEIYDNIFCIGDANGKQMLAHTASYQALKVIDFIEKKEKKNVNINVENNLSKPILYKNIPSVCYTNPELAFIGLTEKEAKVLYPDNVGVEISYYKSNSKILCENNISLNNNKKNNSYNKGQYNINDNTNGMVKIIYKEDTKEILGMFIVGNYASVLIHEAVLAINLKLSAFDLAYMVHSHPTVSEVLDTAFKFISKIRTH

>KMZ88158.1 dihydrolipoamide dehydrogenase [Plasmodium vivax Brazil I]

MGAGTVIWLGCLLLLVTQNGSAKRTGGGHALHLLHAKGDPAPSGKRTSSAFIRCSSSIGGGIGGGIGGDIGSNIVNSMDSSALTWGGRTSRGQSLRVRGAAKGQAMSVSAEEYDVGILGCGVGGHAAAINAMERGLKVIIFTGDQDSIGGTCVNVGCIPSKSLLYATGKYRELKNLAKLYTYGIYTDAFGKNGKSDPVERNQMLADTVHLDIAKLKEYTQRVINKLKGGIENGLKNKKFCKNSEHVQVIYERGHIIDKNIIKGEKSGKEFKVKNIVIATGSTPNIPDNIEVDGRTVFTSDEAVKLEGLQNYMGIIGMGIIGIEFSDIYTALGSELISFDYSPQLLPLLDADVANYFERVFIKSKPMRVHLNTRIEYVRAGGGGQPVTIGHRERSEGEGDTPGYAANQIRETHVDSCLVATGRKPNTNNMGLEKLKIRTKRGYVQVDEHLRVQREDQGVYNHIFCIGDANGRQMLAHTASHQALKVVDWIEAKGGEALKSDPSNGSHSDWASKPIIYRNIPSVCYTTPELAFVGLTEKEAKQLHPPENVGVETSFYKANSKVLCEHSDVSFPSLSKNNSYNRGKYNTVDHTTGMVKIVFLKDSKEILGMFIVGSYASILIHEGVLALNLKLSAVDLAHMVHSHPTISEVLDAAFKAIARVRTH

>SBS82942.1 dihydrolipoyl dehydrogenase, apicoplast, putative (aLipDH) [Plasmodium malariae]

MAIDEKEYDIAILGCGVGGHAAAINAMERNFKVLIFVGEENSIGGTCVNVGCIPSKSLLYATNKYRELKNMAKMYNYGIYSNLFLNEAHRKDGSDDNSSSSRSIRSSLSSLSSLSEHDKMRSNQLVADSVEMDVEKLKEYTDSVISKLRGGITHGLQKSKFSKNSEHVQVIYEHGYIIDKNTIKGKKSGNTYKVKNIILATGSTPNIPENVEVDEKSVFTSDQAVKLEGLRSYMSIIGMGIIGLEFSDIYTALGSEITFFEYSPELLPMIDSDVANYFEKVFLQNKPVNYYLNSEIKYVKASKNNKPVIVGYVERGLTGGSSSSSSSSSSSSSSSDSSGTSIPSQIKELHVDSCLVATGRKPNTQNLGLENIETQINNRGYILVDDYLRVKKKNDEIYDNVFCIGDANGKQMLAHTASYQALKVIDLIEMKEKNILKESAKNNINKPILYKNIPSVCYTNPELSFIGLNEKEANKMYADNVGTAISYYKSNSKILCENNITLHGQDKNNAYNKGQYNITDNTNGMVKIVYKKDTKEVLGMFIVGNYASILVHEAVLAINLGLTAHDLAYMVHSHPTVSEILDTTFKAISKIRTH

>SBT75910.1 dihydrolipoyl dehydrogenase, apicoplast, putative [Plasmodium ovale]

MKVQINTEVMVLIWLVFLFLLSPSSGLQKEFNNGLGVSLKGVEKANHFPHLTIACTHRSMFPRKGKNVLFVKSKMKMGNIRSKRNSNFSYLSGENSSQGMTIREDEYDVAILGCGVGGHAAAINAMERNLKVVLFAGEESSIGGTCVNVGCIPSKSLLYATNKYRELKNFGKLYNYGIYSDFFMKKESNNVVTSPMESNQIIANSVRMDIDKLKEYTQTVVGKMRNGILNGLKNPKFSKNSEHVKVIYEHGHIIDVNTIKSKKSGKKYKVKNIILATGSTPNIPSNVQVDKKYVFTSDQAVNLEGLRDYMGIIGMGIIGLEFADVYTALGSEVTFFEYSPDMLPMIDRDVAKYFEKVFIKTKPVSCIFNAQVKYVHAVKSGGPVVIGYVNRSAVQGAHRRGSDTIGEDATVGEENAVKELRVDSCLVAVGRKPNTENLGLENVQVKTNRGFVPVDELLRVKMEDNLKSRVHENIFCIGDANGKQMLAHTASHQALRVVDYIENKEKNHINEFAKNKLSKSIFYTNIPSVCYTNPELAFVGLTEHESEKMHPDNVGVEVSYYKANSKILCENNISEHGHKNNLYNKGQYNTNDNTNGMVKVVYKKNTKEILGTFIVGNYASVLIHEAVLAINLKLTVFDLAYMVHSHPTVSEVLDTAFKSASKIRTH

>6I4P_A Chain A, Dihydrolipoyl dehydrogenase, mitochondrial [Homo sapiens]

MASWSHPQFEKGALEVLFQGPGADQPIDADVTVIGSGPGGYVAAIKAAQLGFKTVCIEKNETLGGTCLNVGCIPSKALLNNSHYYHMAHGKDFASRGIEMSEVRLNLDKMMEQKSTAVKALTGGIAHLFKQNKVVHVNGYGKITGKNQVTATKADGGTQVIDTKNILIATGSEVTPFPGITIDEDTIVSSTGALSLKKVPEKMVVIGAGVIGVELCSVWQRLGADVTAVEFLGHVGGVGIDMEISKNFQRILQKQGFKFKLNTKVTGATKKSDGKIDVSIEAASGGKAEVITCDVLLVCIGRRPFTKNLGLEELGIELDPRGRIPVNTRFQTKIPNIYAIGDVVAGPMLAHKAEDEGIICVEGMAGGAVHIDYNCVPSVIYTHPEVAWVGKSEEQLKEEGIEYKVGKFPFAANSRAKTNADTDGMVKILGQKSTDRVLGAHILGPGAGEMVNEAALALEYGASCEDIARVCHAHPTLSEAFREANLAASFGKSINF

**8. Phosphatidylinositol 4-kinase A [EC:2.7.1.67]**

>XP_002258257.1 phosphatidylinositol 4-kinase, putative [Plasmodium knowlesi strain H]

MGRNKMEERHRALKDVNIENIFKHVVPFGTGGRKKTALIFYLCNSLEERHLERNDILLLYLSLLPSFQGHLEKVFQERRFRWRRQEKGYVHVEESELELALRLSKLNLLFSYIITHISYFVSLSRKKKEDTSSLQLFIEHMHVRTFLTLQKFVYEKKKRNQLEKLITLNHLKILLTFLINIYKYKIYQVLNEEKLYIISKFIFLIIYFFCHIKDNSKTEKLFICFYDYVYHVSEFFISKYLYYLGRNGKDANVLLLYYQNMISFVCDYMGKLKILKHVERYILNNGKSPFCSVTLLNVVKNTVERHLLCAQGEEDHLMKLRRREVSPPSSLHNEGSLIADQKNGKKDSILLMHSKTVSQFVFLKLLRLVEILLVKKDRIYLTVYLHETIRRVTKSTLEEAFLILKGGEDRKCKNNMMMIYDRMFHLYEYYCNTLFYIINRSDKRKIRERRGEFNTSGSTNTTYMYLLNSCIFNLYEIYLVLILLLHVTPNEVRTILGCNKFSLVKKMLRIHIGEMEVTRIVNLSIFFFDVVLKNVMKSATMGHYAFTILKCLLWNDKKLSKFFQTMVGIYGGKIKGCAIIGWKENSIPRVNCLEEADRTEQNEEPQRGYSHTDVLTSHGDRVLREASLSSANLIVNEKNSFVIQVNRYMKLNWAHLSGLSSGGMGDVPDGAVCVGSTSNRTDGEEVKDELDLLKRLSRSSTNRSNPRCSRGDVRGREEIYTKCYFPLLMKHLLSQLIKRKKKKRVQDDVESNCKNYLAIYINKLHYYCNNIFTYKPMFKYNFSSFVRSHMTKKKKHLIENVTFFNRMFSLYSNKKKGSDKKKKKNFFFTNKSFVISNIERKYSYSNYTLLRKIFNYKISYFDNEHYYMNFLFNLKFLHNLNDYINEYFLLKKKQLNKKAIFNISLNLKDKIKKMKPAPNDLYGHPVGNGSVHSLEGFGKTDLKGHWKSENIVKDFWDSNVCIYSRQKRREKEEGNMLLHFLFINYNYCVCRKDPGEHMSRFFLLILKSFDSVIPYVHIYKYICALLLFLISFVERKPLSRRDVLRVGGGASTTGKEAEMAAKMATTVAATEMATTVAATEMATEMATEMTAAGTADCLNNAVEVGEKGDPTKYGKNTWQERRDGGKACPVGGSAVSRILLQGVAREYGRVEEDVMGEMKRVGVGGGKVPLGKYPLLQRTSNRKAALKILMGTVDRLKTHLFSNTFHYLYQDVKKYNKYVAMLSLYFYKLYFTLLYYEIYEKDYLHGDEIDMLKRKIAEITPILSVKYLNRNNIHSVCNLEIIKSIKEDRTENNDQLQVNSNDVEKLYVIIMHYIESMRRKEYILYSLYLLIDKAIFRNKYIHKNVKKLIDFKVYIFLHDMREFHDYSVIFSTFFLQMNIMSYNKIIRNYIYFIFDQMFKLKFNVLLDGNFLTCFLKNLEKSSLDYAFSKMIYEEKKKFKNFIDKKKSTMIKIIYKVNLYITINYLLFLKMLNCCKFFLNLYSFFYYEFYIFCVNKYVKRYRGREDDNNDDSDDGDDIDGGCYYGGTSELRGAKRLSSSCADRRDKGTQGISALSNGSVGAEATELLKGEKHKDGSAPRVRSTPLSIDKRSFTKRSRSRKTAFKGSRSNHGIIGRRRRKINSNFFQTNKEFSEFFENTSYESHKSLRMDRAHRPEQSRRARRVMPSETQNNESNYFKVLKKIRGGNTFICNLYDVKISKKMVYNKKHIKEYMNTINFIITIFLTTNINNLNMDTLNNNYMYIISKIIKKNFKFYFFYFYYNIFRKKSKRFKSIYSVNVDTIVNNSFGGSATIGMNDTLGRNATDTRNCNISKLSIKNKYIYVLYTLSTILSVYNYKYAFFNYYFFVFYKIVKMLAMMTARSISIGNYLVDRCGGLASVRGEHTDCAGKDAVEGIVPDHLQRRRAHCILSHLSRAERRFLQSDLLMIKRYVNDYIRESKVQINRNLFRSGVPHKYVLERYTTLINNILLLLLFTNYLSLTYHNINLIENKIVQRKINLLEKYKHYLMNRILQIYFLNIPYLCNYLVVTLEFCLLFSRTLLTLNYSSFSMIKEMKGIFRNGYMYRLVNYLNALQNMKTFYHLSFYRSANLYFHNYKKVADEKFDCFGAGGIVGVGRKDIDVERSCSSVHRGKTTLERPAEGDSDGSLSEYDDYIYKDAFKEVKEVLKKKTKQYMKDYFEISMKNIFYLLDCFKDMSTISLFLTSYLYENANIRDMYGTQHIQSMNNIYLYNFKALKMNYYFLCSVFLSVVEIFNYTSNFFHSYNYLIDLYGKYKYSYFLYDYSLRNFFSFFNIYMFEMSRCRQRKRTFLNLKNQVSTVCIGDIFDFNKLLLQKGFLFLCFFFYNFENVLPDIYCVNCANKYSIGCYDRNALPVDLDFVRHSRKGKATVRGVSKEDAVSGVTMGPSTGIVNAQSFLGKRNRRKVYNHLSGSGGEDTIAVKDSNREGKISQGGYFEMFYSRERNKEMAVSVEHAGGIDPLETETHMSRGIRKIDEEVIHFGENRTNGHFEEIDAQEGDHSSGEKLTLEEKNSHSRDRKLSRDVSTPVRKNGSKTENGKANEEHSKPTGKYIGGIISYFKNKVTRMGNKKGGGGAKEQVKKKMRNGYSYYGDSVDAEEHAKCSSCGANNEWGGKNLVRDSSTAERVVNMRGEDYGIYDREGQSVQPGQPSQHRDGGDEGELCARRLREKDVELLKAYKYLLAYQKGNKRRLYDYHLKEELRCMCINKYIHVCNLVYNCLMYMYELKYKYKDMKKRRFCLFTYTRLTRKKEMESMNRSKVKNEIVINTRHLLNLDFMIMSTLHLAESIYINLYKCKLFTDMCIFNNIKYVCLENSEAKKKNSYQNGTHIVHSGSNCNNVTAISERKHRRDVDGSAYHSDYLTYEESANGGSSLSTRDVPEEEGDDCSEKMKRGARIRRTTSLDVVSGRDSDSDSSDDSRKTRRGKKEKSKGKSSRRKKRSVRRMTFFLRRRKKHDSSKSSKNLSINNYLFAEKSVSKKSCKIKEPTINYFHNYKILSDKYKAKLARMKKKKRKKEKEKEKEKKKKKEKKKKEKRKNKGKKKGDQSEAIRSTNGNAFYYYDGIYHIDEDHEDEHRPSGKSKWNMRRFFFRRKRGEGDPSDGSAYNERGNASSVYRASDPVRINNLFVKQQCRRMKNNVMNDLLRMEQGNLEEENMDQIKQLFITFNNNYKEKKISGYISNIFSYTSLHSNFNIFYRRHFEMFGHKVKVGRGLLKALYSYYYRHNLSTFYKLFKKIEYIYVYLNLHFYNSLFNMDVKEVNEDIEKYSLFMVVKYIKRHMHNNPKLYSTFIVRVLNCVNSAVHKMTNYLCMYLYKDLMNILHQYLIQVSISNKLTLNKYNLFLLKKNKLTYVPISNILFLHCNQLSLNDIMKILYSHPQYGLFQRTLAVSNLLYWYKKSTITNCIYLQLFEYLKVDLGNRVFFFIVFFSLQSFLFLYQFFLNLHTYIDDEYLVDFASSSPYEGKRHHGIQELTDGGREEETRIVSAWDDKHIVSTLDEQHMHHQCVLFNTEMFARRTNPLKNLLMLLFPKSKRNPPTGAGRTDKHGDNTTSGSKHQKEININNFIFFKYQSYVKKSHVLSIKCFLIKKLLVQNLSEKNRKALIESCRFHNYVFYTSQRAAQLDKNIQEDFVKMEMQNFFNNMDVRKIKLLTSNNIVWGVSDEVKILMSATRTPILLTFKTFKKEQLVIDTPMSGTTLGCIPGSHSKEVMPSDHGANERKYHRMWSLPNVRMAISNGASDNAPPSKKNDRSLFVEGEKTPEEDTRSGTKKRLHSYYLSREEHKGGLGTYEHAKTDCVKRSVKLAKQHMEGSSSLTLPSLQSGLLTCPQRNLNCQDVSYIYKVNDDVRQDKLVIQIIHIFIHILSDYKSFYNLFPYNIVTNKYSNVHVKGAEGEHNGRSAENEAKTQNGRKFSFSFRLFGRRKKGRNDQRNDESNDKRNDERNDERNEQRNEGGAKGEVSKKDFSEGNTSKRDRPKNSTIPRGTTDQGDENTPLREEPHSPVKRSDRGNGEKQEIKQSRDAPQKEHFERTIIPYVPEHVSGYSKQVETLTKKKKKKKRKKFENFGAVIEVLANTKSRHEIGRKYKNIIKFYHLKFSHMNTYIYALKNFICSLAAYSLLSFVLQVKDRHNGNLLFDDYGNIIHIDFGYILNIYPGISINFELAPFKLTREMIMLLTIKSQKKQYFIFTYIQLVVKGYLLLREKSDWLISSILSLSHSDINCFKYNTVEKLRKRLKLDKSDNDASIFMINKIHQAYNNITTIMYDYIQNIQQGIQ

>PKC48731.1 phosphatidylinositol 4-kinase [Plasmodium falciparum NF54]

MERRYYLKKNDKIFKKVNIEDILKLINNNENGIRDNIMLILYICNNLEEDHLNYIYDDVLILYLKLLPILEKELLEKREDRNIENESNRINKHAYIKNDVEDEYFIRMKRRILKYNYIILHLSYFVSLKKKKNKIETLKLFLYYIHMLTFLSLRKYIQIRNIVHIYNTQNKHNTNNIDYINNVSKFKNEKPIENIQKSHNTNHKGAKTYENQKNVVSFYLSYINILLSFLIYVYKFKICNFVLNKIEFIHISKYIIYIILLFNHIKKENNTDMEKQFLCFYDYVYEVMVLFLDQYNHYYYSIYNEENNIIQKKNVKIIKKNNNKMNNNNNNKMNNNNNNKMNNNNNNKMNNNNNNKMNNNNNNKMNNNNNNKMNNNNNNKMNNNNNNKMNNNNNNKINNNNNKINNNNNNKINNNNNNKINNNNNNKINNNNNKINNNNNNVRPFCAYYKKVIFFVFSSLERFKVLKYIEKYIMNKKKNTFYNMTLLNIIRNTVHIHFLNNSLYNTKDDYINFYCLRLKSKELVKEANEEIGKIGVENSNYINNMCDKKNRNVICLNYYNINNLLFLKLLNMCELLLIKKKKYKYEDNIVYTNKNRNDLYLCIYIYENIMTITKKTIEYMMCFIKNEENINGEACNKYISYRIFFLFEFYFNIIVYILNVKYKNNMEHIVCNNNNNNKKNNKINIVNNYIHNNLYIKYFFENSLYFIYEIYILLILSFRCIQNFEHVLHENNDMFDFFKKMLIIKKTDNNYIFINNLTALIMHFFFFSSPSKNEKKQKNKTQNKDVIQKKTKEKKKNLYTYSSFRLSSRMYFFNMLHNQNKRLNRFLKNIDKLCYRKYNNVVVIIKNVNKYGNVDYSVYQRNDSHMFINPKKTNYIVHNNISYEHETQNNNKIFFNKERGFYINISSYVKYIYNIYNEKNDDLYLNMSKTKERNILMEKDNKVKMDLINYHNNYHNDNDNDMMVNINNFSVYHYYDKSRDEYYREIHFNFFINYILKKYKERKTYIPNYDHCKNFLQIYINKVHYYGNSIFNSKHIFKNNIYTFIKMNVNKKKRHETLENLVPLKKRFYMESRKSINEKMVGVAYNIYKKINNNNDDNENDEDNENDEDNEDNEDNEDNEDNENDEDNKNNDDNIHNKRCSSKYNLFNKIYNNKTDYIKNEHIYFNFLFNIHYLHNLNDHMNEYLLLKKKKTKTKSFFSISLNIKERLWGKRQNHKSAKDKKNKENIKKHKKSNDMKKLCEDNIKDNNLHGCLQKEKKKIKSCIFKKLFDFGVKDEDILYFLFINYNYCILNYKNNKKLKLFLLQILDIFASLICYMNIYKYICSFLLFLISYIERKPIEYLYLSQIFNGVYNEKYIRPYSKDDHENGKNNNKKKKKKIIIIKKKNHHHHNINHNNVANQFIQSEDIKKKIIKNVKCDNRIFSNFNQKDLSKEENNFVYVKNKCNDNIKKKRNYFYENLNVNNILFNNIKINEYDYIEKDVLDYMFRIKEARNNKLHNIQNDENNILDVSYTIFYKNKMIIKIFLEIIEKIKMILCCNIFDYLYKNKRTYGKYVSLLSSYFYKFYFVLIHEEISFNRINKNKYNYIYKDIESYINIKDVNSSSSTTSRVHKQNKKDNKYDDKCCYNYEYMCSFYQSVMLIKSKIIEITPILSIKYLTQCDLYNICKHDILIGLMDKDYKGNNKNRKQDDVIKNKRDVIKNKNGVIKNKRDAVNITNDVNIFNDVNIFNDVNIFNDVNIFNNVNIFNNVDGVHGYPNYYDIISEDNLKKLYIFIIYYIEGLRKKENILYSLYLLIDKNVYRDKYLHKHIKNLIKYKIYTYINEMNSFNDYDMIYSIYFVLINIMNFNKMIRNYLYYIFDNICKIKLNIIFDKSFLFCFLKNLEKSSYDYAYTKILYDEKNILKNEIDKKKNKMIKIIYKVNLYVTINYLLYFKILNSCKLYLYLYSYFYYEFYIYSFKKYIKKYNVYFYFERMKKYVPTSNTYNITKRKKKSTENMTMRNNKMKNMGEKNIDEKNISQDIKNNICNKKLFSSSHDDILEKSNDMKGLYKMDNVDVISSHRIRSFEKESICLSDEHMVDDEKKGNLSHERKYKKRDDNINNIININNIININNIININNSCSNNNLKKKKKKKEIHTGNIINDEKHEVDKYSMLLQRNNKKITNKNLTNRKKRNSTFFLTENNNKLNEFFDIIPNNNYNNKNNDNLENNVNYMKILRKAKASNTYIYNIYDIKINKQIIYHKKYFKEYINIITFVICIFLSTNINNLNMSILNHNYIYFISKIIKKNFKFYFFYFYYNIFKNKQTSNRYKNIYNMNFHVILNNNIPNYLSVSKINIKNKYIYVLYTLSCILSVYNFHYALFNYYFFFFYKYIKILSKFSAKSVSTCNLMIHICLSEPMDILYKGKENIRKDKINKNKCDKDTYDDYNNNIMKGWERKELYLFNNYDEEEKMIKLNFNEKKKGKKMKRRKTYYEEGFSFCDILMIKYYMRKYVRLEFVNIDNALKNKTLSNMKILDMYVKYINNTILLYIFVNYLIYIYKNIRIKKKKGNFKKEAKIRRIIKYKLNILETYKYYLMKIIIDVYLLNISYLCNYIIIVLDFFFIFGDSLFSINCYNHNILTDVKFAYRNQCQYFINNFFNKLQNVNTMYHVNFYRSSNLYFHDYKKKKKKEREKENHNLYHHHNLNNNHNLNNNHNIYHHHNRTNMMIKNHFNLNCQNSENEIYRNNIYNRYQYEDIIDQNNDISCDKNIIMLKEKKETNCLNNFFVEPTISSFLDKNNNSDDNISIVDEYIYKDCFIELKNILKKKMKEYIINYYNRNIKNIYYLFICFNDINNIILFLNLYINKYIHLKDKYNNIYIQSINNIYLYNSKLLKMNYNFICILYLCLIYIYNYIVKFFHSYSHFVDLYSKYKYNYFMYTNNRNNFFTFYNIYTYLNTSKNKLDPHIDKINNDNNNNIYYYNKYYHNNNNNNCGSNNLYDEITNICIRHIYDFNKLLLQKSFLFLSFSFYNYKNILSDIYCNNCIHKYSLGSQEDKDVLNIDISKLNEHQEGHKKNKIKFKKKKERKNGEHIYDDNKERKNISSSIEWFDQHVHQNNIEKKNNNNNNNNNNNNNNNNINLSNKHMIHTDQMPSNNYHTVDANKKEINKNSYNLLSYFKRKMNIFKNKNKNDINNTIYMRHIQHGDNKNIDTSSYKELTKEKEEKYKKKGYNMKDYEKMDSHHIDSIKKKKKEKKKKKNEKNGNCNDNSNELVQTKVDVYLDSTTHECTKIYSNGNDEEEEEEEEYTLKKKKKKTFNRKKKKTKKTKKKRTKHIKEERYIEYPSDKEEDFSLLKNKEEDTLLINAYKYMLEYKKKNKKKIYDYNLKEYLNCMCINKYIYICNLIYNCLIYILQIKYTYNEKKKKKIAFFSSLGGFQKIKKSFSLYNDDINKNYIVINTKHLLNLDFVILNILSLLENIYINLYKCKLFTDMCIFKNIKYICLENKELKKKNKDLSQNKTKKVKHIFVQHPKKKKYQNGYVDKISNGNYESDLDYYLSDALYSSGSVYNIPLYNRRETKKYVKDLKNYHDNDINIPSCDEKKNQNDDHIKVMLSNYNLKKYNKYNNYNKYNNYNKYNNYNKYNKYTEKNHLLNNNHDDNKWETLLNTSNIKDKNENTFNYCEPKKERTRRTSLMNEYYIPKKKKKKGYKLSINVKPISIKKNMNNKNEEFDQGLSNKKEIKKKNNKIIKKNKKKKKINDISLINFQNYKILRDNYKKKINKKLKKKKKELYYYKNIYYNVKKKKSIYNKKNKWNKFFSNILFFKHKKNNRQNNNNGIINNKKKINNNNNNNNNNNNNNNNNNNKNNNIHNNLHRYHYKKSTINKNIGINNLFVKYQSKTIKNKNINKLLKLDTDKDENIERIKHLFITFNYNKEKKINNYIHNIFTYTSLYSNFNICPKKNIDILNYHIKVRRNLLKLLYSIYYKHNLSVIYKIYKKIEYIYTYLNISFYNNLYEFNMKDINENIDIYSLFLIIKYLKYHIYSNEKLFNTFIVLIINFINTNIHKVTNFLCMYDYSDLLNILNHYIINIDINNKLTFNKYNLYILRKNKITYVPISNILFLHCNQFRLNDIIKILYSHPQYTLFLKTIAINNLLYLYKKNTITNTMYLQLFEYLKVDLGNRIFFFIIFFSFHSFQFLYQFFMNMQSYIRNEYIGNDIIYYNNPNNFNKEQTENKNIVHKYQQTHDKSLELALNTNHDHKENNMLCIHENNTMNDILFKKELFVKKKNFFKDFFLFFMVKKNSLLLLSNDYKDKKNTNNIININNFIFYKYQGYVKKTHVLSLKCSLIKKLFLENLHDKYKNMLFESCCFHNTIFCISQQASKLNKDIQMDYVKMEIQNLINNTNINKLKLLTSNNMVKNVSDNIKILMSATRSPLFLTFKTIPPSLNQLLFVHPFVQNNHTQHNYININVHNIDQLNIYTNNELYRQQLNKNILNNVTFVNAQEFAQTNEPNNNQYINNVKCNSYSYVVNRNENKGNIINDMNCSGKIMGKNEMNENKENKENKENNENNENTINGMNCSGKIMGKNENNENKENTTNDMNCSGKIMEENEMNEKEDNDEEKEKTFEDISYIYKVNDDVRQDKLVIQTIYIFIHILNEYKLCYNLFPYNIITNKFVNIYSSNEEDEKDEKKWTIGASQKKEKKKRKNKSATTPKKNNKVPNNDDQIDDKRKFSFSFNLFKKKRQGKNIYNKEKGEKNKEKINTGINVNINNDDKINNMNHEVIIQNDTLLNVKNCNTHKDEDKIVTYYENEDSVTEQEFIGHSRNMHISNVKKKKKKKNKFENFGAVIEVLTNTKSRHDIGKKYQNIIKFYHLKYSNIIVYIYALKNFISSLAAYSLLSFILQVKDRHNGNLLFDDNGNIIHIDFGYILNIYPGISINFELAPFKLTREMIMLLTIKSQKKQYFIFSYIQLVVKGYLLLREKSDWLISSILSLSHSDINCFKYNTVEKLRKRLKLNKNDNDASIFMINKIHQAYNNITTILYDYIQNIQQGIQ

>VUZ94185.1 phosphatidylinositol 4-kinase, putative [Plasmodium vivax]

MGKHKLEERHRALKDVNIENVFKYVVRCGMGGRKKTALIFYLCNSLEERHLGRNNLLALYLSLLPSLEGHLGEVFQRRASAGRKEQKEGGPPHAKESELQFSLQLSKLNLLFSYIITHVSYFLSLSIKHKGDTSLELFIQHIHVRTFVTLQKFLLQKKENELQKLLTLNHLKILLTFLINIYKHKIYEVLNEEQLYIISKFIFLIIFFFGNIGENFKGEKLFVCFYDFVYHVCEVFMSRYVHHLGRNGKGATALLVYYQNFLSFVCDSMRKLKVLKHVERYILNNRKSPFSSVTLLSVVRNTVERHLLHPQGEDAPLMRLKRSETSQSNPPEGSNPTNDEQNGENSCILLMHSKTVSQFVFLKLLRLVEILLVKKDRIYLTVYLHETIGRVATSTMEEAFLIVNGADERKSNPVMAKMMTYGRVFYLYEYYCNTLFYIINRTDKKQKRERRGELGSSSTVSAPHVHLLSSSSSTVNAPHMYLLNSCIFHLYEIYPVVILLLRVTPNEVRTILGGNNFPLVKKMLRIQIGEMEVTRIVNLSVFFLNVVLRNVMGTSSVGHYAFTILRCLLWNDKKLSKFFQTVVGEYTGKADGFALIGWQQDNSPREISLEGAERTKQNEDPQLGYDHSDALDVQGDRGLRRAPLSSANLVVNEKNSFVILVNNYVKLNWAHLGGEAHPGGLPSGSASDLADGAEVRDELGRPSRASRSSTNGSIANRSSTNGGSTNRSNTNGSSANRSNTNRSSTNQSSTNGCLPSRGHPRCSRGAARGREEVYTKAYFPLLMKHLLSQLIKRKKRKAQDDVESNCNNYLGIYINKLHYYCNNMFTYKPLFKHNFSSFVRSHLTKKKRHLIENVASFNRMFNLYSSKKKGSEKKKKKKNFFFTNKTFVLSSIEKKHSYSSYTSLRKILNYKTSYFDNEHYYLSFLFNLKFLHNLNDCINEYFLLKKNQLNRKVIFNISLNIKEKIKKMKPGPNDLCVHPGGDASVRSLGPLGQTDVKRLSKSESIVKDFLASKGCRHWRPKRRKKKEGNSLLHFLFINYNYCVCRNDAGDHMSRFFLLILKSFESAMPHVHVYKYISALLLLLISFVERRPLPHADVLRVGGGAGAAGRGAIKEGRGAIEAGSGAIEGGGGAPNCLANTAELGRKVGRSDPASQEGDRAGQASPVRGNSVSRTLLQGVAKEYARVEEEVMREMKRVGAGRPPTPGKYPLLQRASSRRAALKVLTGTMDRLKAHLFSNTFHYLYEDVRKYNKYVTMLSLYFYKLYFTLLYCEIYQRDYLPGSEADLIKSKIAELTPVLSIKYLNRSNIHSVCKMEIIKSVKEDRTEISGQVEVTNSDVEKLYILIMHYVESMRRKEGILYTLYLLLDKAIFRNKYIHKHVKKLIHFKVYTFLNDMREFHDYIVIFSTFFLQMNIMSYNKIIRNYIYFIFDQIFKLKFNILFDRGFLFCFLKNLEKSSLDYAFSKMVYEEKKKFKNIMDKKKSGMIKVIYKVNLYITINYLLFFKMLNCCSFFLNLYSYFYYEFYIFCVNKYAKRHRHREQDEGGQPHPQLLKGGDHPDGSPAHGSPVHASLPHGSPAHTSLPDASPPRARSAPKRSRSGRAALKGLHPISGLLSMGSSGGRANYGMLGRRRRKVNSNFFETNKEFSEFFANNSDESLQSRQRGQSPEPGEALHPGEPLPPLPPLQTKRARPAMRSETLNNDANYFKVLKKIRGGNTFICNLYDIRISRRVLYHKKHIKEYMNVINFVISVFLSTNINNLNVDTLNNNYIYIFSKIIKKNFKFYFFYFYYNIFRKKGKRFKSIYSVNFDTIVGSGTIGRSTTAGRSTTVGRSTTVGRSTTMGRSTTMRRSTTMGRSTTIGRNTTIGRNTTIGRNTNVCKLSIKNKYVYVLYTLSTILCVYNYKYAFFNYYFLVFYKFVKMLAKMTAKCISVGNYLVDRFGGGAAVPAEHSDCAGGDSTEGSASDEEEKRKARCRLCRLSRLSKTERKFLESDMQMVKRYVNEHIRESNLQMQRNLFRSGMPHKYVLERYTTLINNTLLLLLFTNYLSLTYGSINLGENKMVQRKISLLEKYKHYLMKKVLYIYLLNIPYLCNYLVVTLEFCLLFSRALLSVNYSSFSLAREMKAVFRDGYMYRLTNCLNALQNLKTFYHLSCYRSANSYFHNYKKVPDEKFACFGGSGDICIDERDQSCSSVSEHDEYIYRDAFKEVKQLLKKKTKQHMREHFETSMKSIFHLLDCFKDLSSISLFLNCYLHANAKIRDVYDSLHIQSMNNIYLYNFKALKMNYHFLCIVFLSLVRIFNYTSSFFHTYSFLIDMYAKYKCSYFLYDCSMRNFFSFFNVYIFEMSRCRQRKSNLVSLRNQVGTLCVSDIFNFNKLLLQKGFLFLSFFFHNFENVLPDIYCVNCANKYSVGCNDRNALPVDLARVRHGRKGKTAPRGASRGDPPSGVISGHSSGVVCGESPPGKRKPSKAANHANGSDGEDPPHVDVERSGENAPTVMNSNREGKKPQGGYFEMFYKSEGDKKMATSAEHAGGAGPLENDANVQRGIKTDEEEIHFGVNPTDEHFQQIDAQKGGHSSGEKLHREDSQLAREAATPAHTNETSRRGKKGTQTANDNRNNHNANETRSSAGGILSYFRNKVARMGNKKAAGGKERLKKKTANGYSFGDDFAESEEHARCSSCGGNNSDWGGRNLMSASRTPKRGANMRGEGDAVDESDEENAQEGGNCDEGGGRGTRDGGHHAQQSHRLQHSHLLQHRDTDELCSRRAREKDVELLNAYKYLLNYQKGNKRRLYDYHLKEELRCMCINKYIHICNLIYNCLMYMYELKCKYKDMKKRRFCLFAYTPFRRKRELSGAHSSKVKNEIVINTRHLLNLDFIIMSTLYLAESIYINLYKCKLFTDMCIFNNIKYVCLENSETKKKNSYPHGEHVDHSGSNRNNVSSTSERKQRRDAEGSAYQASANGGSSLSTRDVPEEDCGENCVWDSEGRGEDVGACKATRVNYGGEKMKRGAGSRGGSGKGNGSHICSDSDTESSSDSSGGSRAKRGQNKASSRNGRKKKQQQKQQQKKQKKSVSRMAFFLKRRKKHEAPQSPKNLSVNNYLFADKNVSKKSCKVKEPPINYFQNYKILSDSYKAKLARMKKKKRKKEKGKKKQKREKKREKKGDKGKTKKGPPREAIRSTSGSALYHYDGVYRIDEEHTDGEKPRGKAKWNLRSFFFGRKRRDRGPSPRGSTPHERGNISTAYHSSEPVRINNLFVKHKSRSIKKSNMNRLLQMDLGDLEEENLDRIKHLFITFNNNYKEKKISGYISNIFSYTSLHSNFNVCYRRHFEICGHKINVGRGLLKALYSYYYRHNLSTFYKLFKKIEYIYVYLNLHFYNSLFNVEVKDVREVNDDINKYSLFMVAKYIKRHLHSNPKLYAAFIVKVINFANNGIHKMANFLCLYRYRDLLNALHQYLIQVSISNKLTMNKYNLFLLRKNKLAYVPISNVLFLHCNQFSLNDIIKILYCHPQYGLFLRTVAVSNLVYWYKKSSITNCIYLQLFEYLKVDLGNRVFFFILFFSLQSFRFLYQFFLNLHTYIDNECLLDGEPRGEAHLASTLGGQHIISTLGEQHIISTLDEQHIVSTLDEQQMDHQCVLFNAEVFARRTNLVRTLLALLFPKKSKRSLPPGGKSDKHGDTTTGIGGISSTGMTSGKNQKGININNFIFFKYQSYVKKPHVLSLKCFLIKKLLFHNLGEKSRKALMESCRFHNNIFCTSQRAAQLERNIQLDFVKMEIKNLLNGMDVQRVKLLTSSNVVWGVSHEVKILMSATRTPILLTFQTSRRDVRVVGGAMSATMSGTAVGSMPGRHAGETLAEDRVRAERTHPRMWSLPSVRMAILGGGADGPTDDAPVNGPTYDSPSKPSDSSLLISGGDAPLGGSTPEAATSGSQKRPHGHYLSSEQHKGSALTYEHASVDSIKRSGELPEKNTHGSSCGGTLPSPHSSLFTPPKTNRICEDVSYIYKVNDDVRQDKLVIQIIHIFIHILSDYKSFYNLFPYNIVTNKYGNVHARGAGGDRNEGGAADGANEGKTQNGRKFSFSFRLFRKRKSKKDHPKGEGSKTERAGGNPIKRDPSQNSTIPRGNPDRADLNTPLRGETHLERSIIPYVPEHPSGNSKRVETSSKKKKFENFGAVIEVLANTKSRHEIGRKYKNIIKFYHLKFSHINTYIYALKNFICSLAAYSLLSFVLQVKDRHNGNLLFDDYGNIIHIDFGYILNIYPGISINFELAPFKLTREMIMLLTIKSQKKQYFIFTYIQLVVKGYLLLREKSDWLISSILSLSHSDINCFKYNTVEKLRKRLKLNKSDNDASIFMINKIHQAYNNITTIMYDYIQNIQQGIQ

>XP_028860521.1 phosphatidylinositol 4-kinase, putative [Plasmodium malariae]

MGKYEIKMKSNTFKDTNISSILKYIRCKKNGSKNKTVLIFYLCNALEERHIKHGKDVLVLYLSLLPLFEDNLINIFKKKVIASRRGKYYYEENKEIDIQYSIKLSKLQLYLSYIILHMSYFVSLNRNIKKNNSIKLFLYYVHILTFINLQKYICLNEQSNIYEKLFYLCYINMLISFLINIYKFKLYEILSEKKFYTVSKYIFLIINLFTHIKKNRNSEKLFICFYDYVYNVINVLLDKYGIYLKKQNGNTHDKEILVTYYKNFLIFLYNYIRKLKILKYSEKYLLNNSKNVVYNLSLLTIVKNTVDIHFLRDMCEMKCGQVIAFTLQRGTSKKKEVTPNGNVLMGEEDAARSVHNRANNRSYKRTHSRTYNSIDNKATREVRSGENLRVCVGYETVNSIVLLKILKLFEILHMRKNRVYLSIYIYNLVNKITNQTLQELYYVVRKNKQTEENHLYYNRLLYLFEYYFNILFFYITTSPNEREKNRAIYHNNSYCLYLIQSSIYFIYEIYMLLIVLLSTVRGFNVLLHSNSTSTDNPSRRSNNFDFVHKMLFLERSENIVRMNNITSLICDCVFVNSSFYTLTFLEIVYKNNEKLNFFLKHKLHTYIMKGEEVTVIINKKNTYMKKECTNMLTSHEQREQHNHDETTILVIQDHHFNMLNMNRTNLFWDAHTGLYTCVNSFVKLNFIFWKNDDSGSCVYPESNDIRGTTYRNDSVEDSGNMYRRKDENALYRGGRKKRSCEYAPKGIGSFEKKENETDIIHSNVMRSKMTNDDRWEGQNLNRPQNDKIIVDKGKYRRNERKLNGENYIDIYFPFLLNVFLSSLLRKKKKKKKSTQYNNIECNCKNYLDIYINKMHYYCNNIFNCKAIFVSNFSSFVKNSFRKRRSNNWIDNITSFNGMFSFYNNKIRNEKNKLFFKNKTFLIRSMEKKHSYSKYTFLKKMLNYKSCYFDNEKYYFNFLFNIKFLHNLNDTMNEYFLLKKNKINRKAIFSLPLNIKEKIKRMKPIPFDDYSHNHLPMTNFSMLSLDHIEERRKLSKSKLIISDILSQEWKYETRKRKKNDEKYLLYFLFVNYNYCIFNYEKNDNLEYFLLRILRCFEIIMPYVNIYKYICSFLMFLTSLLERKPLADLDVFIINRSVRAQKEVHGCTLCTSLDEAEEKVVEEVVEKVAEEMVEEAADEMAAHDMGGGKSVKWSEGMREIMSSRSMGKRRGVTQSKEKRWRGYNRSECTFNKVQFSKIMVNGIEKEYRYVERNVLKNMERMKEDNCYCIFYKSNYKIIWNIMIGIIDKIKIYLFSNTFHYLYKDKRKYHKYLSTLSLHFYRLYFTLAYYEIYKGSYWNNYDYSTSTRKEIQEIKNKIIEITPILSVRYLNIYNISNICKVDILKSIKENNLLKNDKVNINCSDINKLYLFIIYYIECNRKKEDILYSLYFLIDKYIFGNKVLHKNVKKLINYKIFVFLNDMNNFHDYNFTYATFFLLMNIMNYNKMIRNYIYYIFDQIFKLKLNTLFDSKFLTCFLKNLEKSSQDYAFSKMIHEEKKIFKNSIDKKKNNMIKIIYKVNLYITINYLLFLKMQNCCKFFLNLYSYFYYEFYIFCVKKYTKKYNGYHYNRRMWDWSKSNRMDSNVKNRTKLSSRRNSMCSNVNHVGHLRRGQSSHYHERCYKRSAFDRNRSPSCHHFMAHDPIDSEEEEYNLNSKELLDGIVDSKGYIMVNMSEVFLESNELISDEGLNKENNTEEYICNSNIRLSDTIGQTHLFDKDKYVRREKHQSVVNNAERKYKRAYENLPKGVKISKHMVTYLKSNYGNNRMKSFRKKYYTSSIVENYQNYNITLAALNTTNTLFNRRRIIRGRRKMRKKQKKNSFFFESHKDFNEFFDSQENEAYGTDGADGANGIDSINGINYTNDENGAKLANNVNSGKRVNVTNATNGSNDTIDDDVNYFKILKKVKGNNTYIYNLYDIKINKRVLYNEKHFKEYLNVINFVMCIFLATNINNLNMSMLNNYYIYIISKIIKKNFKFYFFFFYYNIFKKNNKRYKNIYNINFDIILNSSFRNNLGVSKISIKNKYICVLYTLSCILTVYNYKYAFFNYYFFYFYKFVKLLAKLSVKSIFVSNFLIDKLGNEAKGRAMKGKVGKQAEDLDEAEGVTEKETTYFVDKGRNHYDLYYIDGSSNRVSESVIDYGFRSNIIFNDHFEGARKCGEGENVNPNGRSAYFSHMRREDGKGGMNGKDRKDGQLAKDLKDGKSSADNSFTQRSAHRGELRYPLKKKYSKKVKAEMGVKSIFSKEERIFIASDIMMIRSYVNDYIRMDLYVMYNNLQHNNVSNKSILDKYITLINNTLLLFVFVNHLILMYGNINIKKKKIIKYKIKWLQNYNYYLMRKIVDIYILNIPYLCNYLVVTLEFFFVFSNSFLSLNYGNFFLKKDIKFFFKNCYIYYLMNTFNKIQNLKTFYHLNHYKNYNLYFHNYKKFRHKQFSPYFKKSTLDYDKGHREDLQKRMDYSNPLHVCTSTYSNYYEMNSLNNSVIDDESEDSISDYDDYIFKDAFKDLKEIFKRKTKNYLINNFGRNMKNIFYLFDCFNDISSITLFLNLYIHGYTKIKDAYDVLPVQSINNAYLYNFRLLKLDYHFVCILFLSLIRIFNYTSSVFHAYSYFVQLYSKYKYNYFLYSDRSYSFFTFYNVYLFFISKNKLKNENLVSINNHVSSLCMSDIYDYNKLLLQKSFIFLSFFFHNFNNILPDIYCINCSNKYSIVTHDKNVFPPDLTQVCAKEGRLKGKGEEIGKESGGEEGTERMDCMNNATIREANYSITRGKMKKYYKEITHDKNKLCVAYRSYADKDKQEGFTNGINKDREDKDYHKIFFKNYGEEFINSGNERSNAEIQDQRVEEGEKEKHILNHCAKIRMGGKEEAWFDDNHMPRSDNDKWDYINYDEVIKRNDKELLEQNKANDGDMNGKVEEKAEEQKNSIFRKYRDNFFSIFKSKISDQDSKKEEEKKQQEQHHSMTVMTMQGKGKNIIPTEYGKGARNVNKLKQSSVNQNDYSGRKLRKGHSSSKEDISNCNIDRKKSIENEYDSDYFSSQKNTKNDLIILSAYKYMLRYQKRNKKSVYDYNLKEQLRCMCINKYIYICNLIYNALVYILELKYKYKYMKKKFHLFSYNTFKKKKEFNSVSTYNDQNYIILNTKHLLNYDFIILSILHLTESIYINLYKCKLFTDMCIFNNIKYVCLENKGTKKNSNDSYKNININVTNKNLQCNSFFNYKNYLNNNIDHEDYDEDDDDGDNDDGDNDDGDNDVDDNDIDDNDDGDNDDGDNDDGDNDDGDNDDSDNDVDDNDIDDNDVDDNDVDDNDVDDNDVDDNDVDDNDVDDNDVDDNDVDDGDDYNVYNSDDFSCGGSMYNSNSHSMHTVHYAGEKKKKTKTNKRKKQDRFTKASANTVPNMSNSNNSEYKYVSRKTCEQDDLHLVSNSSISDKDNIIQSSSNYFDEKSNKDDLKENNGEELYCEKEKREEDGGLLRSNCSKKKFYEKTISRKTENGKISNNRRSFKVGTKVRRKKKVDENKKDTFAGENDKFNYNNNRGISKNEKKGQVWTVIETVDTKDDSKSQGRKNGTSGTKWRGKRRKGKKDEMSKKVRVGGKGKSENGRMFFSKNSEKSNSSKRFKSLSISGYLLSVKKLAKREEMKNEMSLGYLHSCKVTAQGYEKRLSRKMKIGKKQRKQNSSDKDGEDVYSHKNIYSISHRGNTYKEGKWNIRRFFFGKKKKNSSEGKVNNDNTRINNLFVKYKSRSIKNTSVQKLLKIEIDDDKEENIERIKNLFITFNSNNKEKKINNYIHNIFNYTSPYPNFNVCYKKKFEILDYKIKVGRSLLKVLYSFYYRHNLSIFYKIYKKIEYIYTYLNLSFYTNLFTTNTKEINSDINKYSLFLLVNYLKYHIHNSDKLFNSFIVKIINFLNHNVHNITEFLCMHHFEDLLNILNQYIITININNKLSINKYNLFNLRKNKLIYVPISNVLFLHCNKFSLNDIIKILYSYPQYSLFLKSIAVNNLVYLYKKSTITNAMYLQLFEFLKVDMGNRIFFFIIFFSFHSFRFLYQFFLNMHTYIEDEYLTHLTYCEMLNNGKEGCVVAFPNGNNNICNSNNISNNKNNYNNNSVLSVFEDNIENCLLFKKELFAKKTNPFKDFLLLWTKKKNNNVSHSTSSSSSTSSFTSSMDNNNKNRKKRITKNYLNINNFIFHKYHNYVKKTHVLSLKCSLIKKLFLQNLCHKNRSALLESCRFHNTIFCMSQEASQFDKKIQLDFVRMEIQNLLNNINIKKLKLLTSNNIVSHVSDDIKILMSATRSPIFLSFKSMKQNVKKNLYLHSLLTRYAIRSRSRVSADDTKSILSLNTHDGVYNSACLMSSNTVRKNSIEGGGNIVQGDMVINNSFTSIANFCSNDEHSSSKGASNVHEQRNDGFVLPRAEEHTIDAVQRSKTSAHYSERGKISSAIDAKIARMNKNIPSLRVYDKSLIHRNFYKTKKEDNERGEDEVGEVYKADEVKGKKKQNRTNNSNKKYEDVSYIYKVNDDVRQDKLVIQIIYIFIHILSDYKLFYNLFPYNIVTNKYFNVYANEKEDNSAHKKITFIKRERKKQSNEDISNVNKETDGKRKFSFSFHLFKKAKKKQTRKKDEKQNKSCSCRSSSKEKEKSKNKKDLFHHIAETEIRDKDGIREDSEKKYPYSSEHFQNNLNLYIQEYHSTTNTKSLSVFSKKKKKKKKKKFENFGAVIEVLANTKSRHEIGRKYQNIIKFYHLKFSNINTYIYALKNFICSLAAYSLLSFILQVKDRHNGNLLFDEYGNIIHIDFGYILNIYPGISINFELAPFKLTREMITLLTMKSQKKQYFIFTYIQLVVKGEKSDWLISSILSLSHSDINCFKYNTVEKLKKRLKLNKSDNDASIFMINKIHQAYNNITTIMYDYIQNIQQGIQ

>SCA48714.1 phosphatidylinositol 4-kinase, putative [Plasmodium ovale]

MRKYTIGRKYNVLKDENICGIFKLISSKQNGSKGKMTLLMYLCNTIEEKHIAHSNNVLKVYLSQLQIFGDILSRTFKKGFLLRGKRRSYYGKKVELEAHPELETEAEAEAEAEAELRHGVELTKLQLRFGYIILHMSYFVSLNEEYKKSSSLKSFVYYVHILTFLTLKKFISMRWVDTYDKLNVLCSVYLLLLFLIYIYKFKLCEMINERKFYVMSKYIFFIICMFSSIKRNRKSEKLLVCFYEYAYNALEVLIRKYSFLLCRKKDNPCYCQLIYHYRNVISFLVSYMSKLKVLKHVHKYMLNDEKNVFCNLSLLNVIRNTIDIHIVRNRLFSDEDRHLPYFTVRKNEGTSKIDRRKQGLKGNLEFSLGCNLMDGKELRGDDRGGDRGGTLIEINYATVNRILFLKMLALSGSLLMRRDGAYLGIYIYHHVNELTNITLQQVRESAMESTCKGEVDIWYRLLHLCEHYFSALFYAIKGSEGEESKCPQRCINRNEYVIHSSIWYIYETYMSLILLLSAGRDIVSLLLGGNRCSGDSGDSGGSGGSAGCGSGYYSLGCVSDNGLFFVKNLLCPRLSKGCIYIGDVTTLIVDAIFASSTFHTPAIINFVRENDYRLNFFLQHQLGACLSRRESSMVVISLGNVYFKDMYTSGVSNGERGCVVGKSSLENLEKEEGDTFDVVRIQDIHFVCGLKRSFPVYVSSHAKLIVCFEKGESSISVEVGSNSAKGEKSIDPVNGKKKEQEELPTSEGKETLDNQESSLPMIDRKLHKRKEELKKGIILCEGTNANKVNEEHHSEFLFSKCHCSMRRYMKFHFPRLMGHLLSELLRSKRKRNPFYKREVERNCQNYLSIYINKLQYYCNNVFNCKQMFHHNFSTFVKSCFGNRRIERERGERGERGERGEREKEREGSMIENVSSFSRMFSMYNKKVNDKSLFSFTNKAVILKSIEKKHSSSKYTFLKKVKNYKSTYFDNEHYHFSFLFGLKYLYNVSDNINDLFILKKKKVNRKALFNIGLNIKEKIKRMKHVSYNDSYGNSFGNFSLGSYEDVGIKSLSKNEVGINAILHEKDKHEAYTPKGKKKNNEEYIFYFLLVHYNYCVFNYYANEILYYFLLIILKYFEGIMPYVNIYKYICATVLLLTSVIEKKPIPYLDLCAIRRRRVSHRKWQSMQSTWIERGKKEGCSNLRETRKGKDKTIDGRHTNEGTQFCNRLNGLKRGYGGVRFGRMVAEGIAKRWYEFVERAVQKDMQRGDSSNSHGGGSRDRSNRRKRYQYGGCIFSGGRHTVALKDMGRIVDNIREYLFSNTFHYLYYDKKRYGKYVGTLSMHFYRLYFTLIYSEVYKNNNMNEISRKQIEIIKKKIVEITPILCVKYMNKYNIRNICKMRILKGVIQNGSMVKAGQAGQAGKAGQAGQAGQAGKAGKAVNMENVGKKAKMKLTKNELHKLYILIIYYIENLRSRENILYSLYLLLDTNIFKYKYLYKYIKKLINYKIYIFLKDIKYFDKYNFIFTFYFLLMNIMSYNKFIRNYIYYIFDYIFKLNIYIIFDYNLIIYFLKNLEKSSLNYAFSKLLYNEKKKENLNYIIEKKKYNIIKMIYKVNLYITINYLLFLKMFNCCKFCLSLYSYFYYEFYIFYVNKYYKKIKNFYPFYTFKNTHLDKINKTYSFSRKEEKCLFLKKHHLNVEEKNLFSIYDKKNFQNTHCEKNISKMEKIQLTQDKLEHYTDYYSFEDISFLENIKFEKCQEKNCISTIEEKFPWDNNTNFDNKMPKIDLFEKHHNNTLYSEKKKTVVGMNMENKGKNSPFQYNRAFSGKYYGGETIDGGVSYFKIVKKVGGSNTYIYNIYDIRESKRVFSSTRHFTEYINVINFVVCIFLATNMNNLNMNMLNSYYIFIISKIVKKNFKFYFFYFYYNIIKKKNKRYKNIYSINFHNILNSFRNNTDMYKISIKNKFIYVLYTLSNVLCVYNYKYAFFSYYFFFFYKLVKMLAKIAVKSISVSNFLINAYTTNSDTTVIFPVRSSASAFPKTISRTAARVVVVREGSNPFHGKTILEKEKRKKRRGTAIFTKWERRFVGSDTRKIGWYVHLYIRKEKLSIRRSIQHGSLSSRYILDRYITHINNVLLVYILVNYMLLLYNNINLGETKIVKNKIKMLSNYNYDLMKHIIDIYLLNVPYLCNYLVVALEFFFCFSNSFLSLNYGNYSLMKDIKLLFKNSYMYFMLNTINRLQHMRTFFHLNHYKSANLHFHNYKKVSEGTTTGDDVSGSIAVADQWKNRGGSDGSLDGEDDDDPIDSASEYDEYIYKDAFSEFSKMFKKKIKETLVNYHSRSVKNVFYFLDCFNDTTCISLFLSMYIHENTKLKDIYNHLPLQGFNNIYLYDCELLNIDYYFVCITLLSVIRMFNYTSHFFHSYSYLIELYSKYKYNYFLYIDNTHTYFTFYNVYVFLTNVCKLTDGECSDVSNNIEMDIIYMGDLFNFNKLLLQKSFLFLCFFFHNFNNILHNVYCVNCVNKYSVGGGDADSLALRPREGHVKNRDAGINPVAKSPVVTSPVATSPVATSPVATSPVATSPVAVSPSGGVRTYLEEGNAEHSGFYRGIPPKGGEVREGNSISRNEIAQMNDRRRDLRKENGKTMKGGDRCKMKYTNESVEVCFDRGEDSLACETTPGAVLHVDGKTKDDDTRHNTFSRYTNSFLSYLKRKVSNFGRRKNKEKKCSTISSYYSKSHEGKVKRVVNQDKLKKKKMQRKRRNKKVNPGDDISNGKSNGKNNSENENEYEYVLSHADRMKDLLLLNTYKQMLNYKKKHKKRLYDYNLMEELRCMCINKYVYICNLAYNCLTFLLEMKYKHKYIKKKFSLFSYNIFKRKKENNSFFNEDNENYIIINMRHLPNFDFLIISILHMTEGIYLNLYKCKLFTDMCIFNNIKYICLENNDMKKKESYRNGDVNDANYTLREKNFKGNVHQSYSIPSPGSVNSSSDSSIQGRYFHNGDMMPGEYSAVQGGGGKELRRMRQQGRSPQGKRKNGKNNGMNNREHEEEKGMLHSRTCVRSNLAVGKQDDVVGVAGVAGVAAEVSNAMAGRKLFAKKREENRGSTRKFMPQSINNYLFSERKMSKKEEKGRDPAVKQLRVGTSFAGGTKRASGEGGKKRGKVRKRGKQQKGKSFSKDAPLGQKKNAKRETKKGKEKEETKKKETKMKETKMKETKMKETKKGKKAKRTRYYEGMYGMSRGRRMWGEDSKWNWRNLFLGKKGRRDDNHINDNIQITNLFVKYKSRGIKNSSIDRLFKIDVDDKEENIERIKHLFITFNSNNKEKKINSYIHNIFNYTSLYSNFNIYYKKKIEILNSKVKIRKKLLKSLYSIFYRHNICIFYKICKKIEYIYTYLNMSFYINLFSIKTKDINDTIDKNSLFVLVRYIKYHIHNYAKLFNVFIIKIIHYINDNIHRIANHLCMYKYRDLLNLLIQYMINVHISSKLTLNKYNIFLLRKNKLTYVPISNILLLHCNKFGLNKIIKIMYSHPQYSLFQKSIAVNNLSYFYKKSTITNAMYLQLFEYLKVDLGNRIFFFILFFSCHSFQFLYHFYLNMHTYINDEYLGHLTFYENCKNNEQTCLIPIDPDGNNMLNICDDNFEHCLLFNNELFSKKKNIFKEFLLLWIKKKDLSNDKNCKNRIINNININNFIFHRYHNYIKKTQVLSLKCSLIKKLFLENVCYKNRNALLESCRFHNTIFCISQEASKLDKSMQLHFVKKELAILLNNINIRKIKLLTSNNVVSHLSDDIKILMSATRSPIFLCFKSVQSKFSRRKFMYFKSPSKSTNILQGIIPKEENNLCSDLGKGKYFEEVLLNSSKVDTCANMTKQIMGELMEERMRYNNLESTDHRQGSIGYYEQGEETNAGVEKKKNACMKEKDLSLDMSNKTFMKDNFINDEKRKKHHFNKTYQDISYIYKVNDDVRQDKLVIQIIYIFIHILSEYKLFYNLFPYNIVTNKFFTVHSNEEWDTSQREEKIFSTKKRKNNNGKTFPNNTPTNDETEKRKFSFSFHLFKNRKNANVSNGSEKNHKKELGPQKIDGRRLIQGDNENNVLVDKKDMSYDMQIQHTRSSTQEYATALSKKNECSSQIKKKKKKKKRKKFENFGAVIEVLANTKSRHEIGRKYQNIMKFYHLKFSNINVYIHALKNFICSLAAYSLLSFILQVKDRHNGNLLFDEYGNIIHIDFGYILNIYPGISINFELAPFKLTREMIMLLTIKSQKKQYFIFTYIQLVVKGEKSDWLISSILSLSHSDINCFKYNTVEKLKKRLKLDKSDNDASIFMINKIHQAYNNITTIMYDYIQNIQQGIQ

>XP_054181676.1 phosphatidylinositol 4-kinase alpha isoform X7 [Homo sapiens]

MREMAGAWHMTVEQKFGLFSAEIKEADPLAASEASQPKPCPPEVTPHYIWIDFLVQRFEIAKYCSSDQVEIFSSLLQRSMSLNIGGAKGSMNRHVAAIGPRFKLLTLGLSLLHADVVPNATIRNVLREKIYSTAFDYFSCPPKFPTQGEKRLREDISIMI

KFWTAMFSDKKYLTASQLVPPDNQDTRSNLDITVGSRQQATQGWINTYPLSSGMSTISKKSGMSKKTNRGSQLHKYYMKRRTLLLSLLATEIERLITWYNPLSAPELELDQAGENSVANWRSKYISLSEKQWKDNVNLAWSISPYLAVQLPARFKNTEAIGNEVTRLVRLDPGAVSDVPEAIKFLVTWHTIDADAPELSHVLCWAPTDPPTGLSYFSSMYPPHPLTAQYGVKVLRSFPPDAILFYIPQIVQALRYDKMGYVREYILWAASKSQLLAHQFIWNMKTNIYLDEEGHQKDPDIGDLLDQLVEEITGSLSGPAKDFYQREFDFFNKITNVSAIIKPYPKGDERKKACLSALSEVKVQPGCYLPSNPEAIVLDIDYKSGTPMQSAAKAPYLAKFKVKRCGVSELEKEGLRCRSDSEDECSTQEADGQKISWQAAIFKVGDDCRQDMLALQIIDLFKNIFQLVGLDLFVFPYRVVATAPGCGVIECIPDCTSRDQLGRQTDFGMYDYFTRQYGDESTLAFQQARYNFIRSMAAYSLLLFLLQIKDRHNGNIMLDKKGHIIHIDFGFMFESSPGGNLGWEPDIKLTDEMVMIMGGKMEATPFKWFMEMCVRGYLAVRPYMDAVVSLVTLMLDTGLPCFRGQTIKLLKHRFSPNMTEREAANFIMKVIQSCFLSNRSRTYDMIQYYQNDIPY

**9. Phosphatidylinositol 3-kinase [EC:2.7.1.137]**

>XP_002259662.1 phosphatidylinositol 3-kinase, putative [Plasmodium knowlesi strain H]

MNNTSIKDVRDYFSVRLSLFTVFKGGHRCDVHSTRRRRSHGVKDRAVDPGEAASSPPDEDANDIAANTANTVSSPNGESKTVFLRKMRRLEKFKYLQRKRKKKHLIMSKDGKERIRPGRGNSKRRVPPIQLKGTTVTIGAGLTSGLDSKLNRNKRREHSDVKYAGEEEIPSDEFEATCYLLMDKEFYSHPVSVRCAASSRRKSHRAKQLSNKCVNHWGENLRRCTRVKKDELQNYDNRIAEDMANILSKSERKSSTSGKRDTGEGEQLFKNSLGGNNLSSGREPHTGGPAKCDSCRDNHSDNSPKENSTTMPRNRKAKAIRRNLCTINRTLNYPIRYSQLSPKSYLFFLIQHREKKDWKFYSYCRVFSPSGAIKQGLQIKKLYHLGRKEKIQDIITSALRKKIAHMDDNSFRKSTSHQSIISFLQGNSVYRDLLCLYRRSRKGSVQRRRDQFSQVVLSREGSIENVRSGASSEMDSHHSILPLREEKKERNVLRGVYEADEMDDPHDTLVDTTSHRSAKGHEKSIFPTGSENPPHRHGTNTFYQTMKQYNRISRYVRSVRRIFFGWDTPVENCLSAGRIICQADDHREVTEENVNEEHRFDFTQGEILMKEEQRKHNGCVSFRVTNLGRKKKYIYMTSQVKRLRRKLRQYEWELFCAGGRIYHEYGKPGHSFEDTLPKRNRVVASLRRISEMYAQWKIKEKAHIAGFLFFHFSLFNERCIYYGDRKGENHQEGAHRKESYPTNCHPDNRDDGDYGSNWFFNSFDYVGEVDGTPGRGTSSVVRAFLKRVKRPVIGGKETLQGEEDLDVGTVEGETPGEGKKRNDIFEHMNRYASRISKNIHLANRSRYDDYPFDFSVQKREGGWHVTADAAPAMEEIKTINTILNTPVIKLNEVEKKCLWKFRLQLVNRQEALGTFVKSVNWENPQEKDEATELLNYWSKPSLENCLEMLNCHLQRTVIKKYVMQIIAQAKKDQLKLYLFQLVQSLRVFNHQPIDDLFIETLINKCITSKKLSIFLHWFLLSETKDKCKGNLYIHIHKLFITKLMTSNLKKKRKILSILKNQNRFRNQLLYLTKIAKSKSDRIYNKTKKLRQFLFCYRQNYGCVVIKDFIKNNIFVSDNEVYDFVSPRDARQGAAAEGVQVEGAVDEVEGVVGEVEGVVGEVEGMVGEVEGMVGEVEGMVGEVEGMVGEVEGMVGEVEGMVGEVEAQVDVEKNVVTMQQVVEEDATVDVDSDADSVHHQNDVCNSVYYLNGELSIHADPEADVYCFQYDPGGTQTAQGTLPLTHRSTSDISTEESKTINYIDDSKGAPIERNKDTSFFSNLLQLNDNFDFFLSATYSDEDNNIDILDDSISLVRKQKIKRIRAPLILPIDPDIEFLSFLPEQSYVLRSSLYPIVIACLVRKKIKLAHEHFHNLIINEQKYLKKNVKKKKNYSMYHSFNSKFLKSLYSSFDCASDFERHYRKVKWEGKNIFYNGRRVEKVGLQKLVPRDGEECPSVDFPIADPPNAQVVIHHETESTSHSKQCVKKYNEIYELSIKKYIYKAGDDLRQDHLVIQIICIIDNIWKRYGLDLKLTLYKVLALSTDDGFIEFVNYAESISSIKKNYKGEIRQYFMEKGTDPKSPLGFDATILENFISSCAGYSVITYLLGIGDRHLDNLMVSRDGCFFHIDFGYIFGEDPKPFSPPMKLCKEMIEAMGGAHSVGYEQFLKKCCLAYKYLRYHSKLIISLLDCMCESGLKDMKMSPELCVLKVQEKFRLDLNDEAAEVYFLSVINTSVKTLFPVVVDKLHEWALNWK

>KNG76755.1 phosphatidylinositol 3-kinase [Plasmodium falciparum IGH-CR14]

MKIRYDKCSSTKDLNYFFHLKLGFFVCYKNHNDKYSFKNKILQKNDTILFFKKKKKFMYLRKKKKKKKKKILIQIIQEYNKYNEYFKYNSNLEGNQGFNKKPEKNKNTKGNVYTDHTNQNAKSKIYNYDMNDDSYSNYVNNNNVFRISSFLILNNEFFGYPLQFVCETEGRSRNHEHYPDVHGDNIKYNKCDDNKYNKCDDNKYDKCDDNKYNKCDDNKYDTCDDNKYDTCDDNKYDTCDDNKYNKYDDDKYDTCDDNKYNKYDDDKYNKYDDDKYEKSRKKKKLNNLYKTILTKKKRKKMNSNLCVINKIYKYPIKYCELNSKAFVFFIIKNVGVHKITYYSYNKLFSKDGVLNQGIQICKLYHVNKNKKIKQIIFEALKNKITFSYDNNPNNIKKKIYKFLKKNCAYHDLIKLFYFKGHKQREKCNKKLNMEKTFGVHKSSRYNYKTYKKKKKIDMCKNYCDDILDTYNSKYYKGELSGQHKHIKMTGEQKEEHHIKYTHLNFNHGKDETFYKELYKCNYIEKYISSVNYFLLERRRMFNKYKQQELCVNKNEENNKNKNDDDNKNDDDNKNDDDNKNDDDNNKNDDDNKNDDDNNKNDDDNKNDDDDNKNDDDDNNKNNIQCDNHSDNIYMCGTYGNMENYNVPHSTNNTNLQSIKKRIINMNILDNIRCNKTYKYIDKNKFKCFTYYSCKNYNVCKKIIEKYKLYKFLKKKKIEGYMILNFLNFNKELIYYNEHKKDMSTLHDNLFDVISNNQNENVKYNHICNNNKYDWFFNSFDYVGNLEESITCFNNHKKKENMKNIKNIKKKKKKNLFYNEQHNIKNNKNDYHFDKYPSSLYSHLTNKKMVNNTEVNNIKDENSLQMYIINKDVTKNKDGNLLLNSYYNSKLGKSINTCSKEIYKEEHKNVYIYNKKITKMNIKMKTEQKYICVDSKRNTRTYNSKNIRTYNSKNIRTYNRKNIRTYNRKNIRTYNRKNIRTYNRKNIRTYNRKNIRTYNSKNFHLNRNKKKNGCVKKYKLYDERNTLVYKNKIGSNHFFLKEEIGKSTKKLNDIFEHISNYTNRISKNINITNKNRYDDYPFDFLSKDKIEYISMLSPTINEIKTLNTILTIPLIKMNEYEKNCIWRFRFQLLNRKETLGKFLKSINWNNKEEEEEAIILLNKWAKPGIENCIELFYSHLHHYVIKKYIIDIIKNSKKEEIKLYLFQLVQSLRTFNYQHIDNLFINTLIQKCIKSKKLSIYFYWFLLSEAKDKIKGKLYLHIHKLFINKLMTSNIRKNKIILDILKNQNRFRNQLLYLTKIAKNKTDRIQNKTRKLRNFLFYYRTNYGYINIKDFIKNNIFISDHNVYDFLDICKMKRENSLDTPMRGDNIGQPSYLGMVPGMGKSTDDSKNVYGDDNKNVYGDDNKNVYGDDNKNVYGDDNKNVYGDDNKNVYGDDNKNVYGDDNKNVYGDDSKNIYCDDNKNVYGDDNKNIYGDDSKNIYGDDNKNIFSDDNKNLYSDNNNNKHIRYNKYVKNISYEHFNEYPYDNKKSRNIYTCNKDICNSIYYLDNELTINYDIKDDLYFFQYKRSSDEKLLNTDLSNDSNDMIHYIDDSKNVKIERNRDNSFFSNFLQFNDNLDFFLNATYSDEDNNYEILDDSINFVQKQKIKKIKTPLILPIDPNIELLSFLPEQSYVLRSSLYPIVIACLVRKKIKLYNENYNNLIINNHTFYKNDQNKDNIINNLSYDKSYHSYYNSQFIKTLQNSFESTTSLNYHYNFLKCSNNNIFYKNKKIERIKPNTSIQKAFPSNENILNRNQHVYYSNNQIVHNIKKMNKHKRDDYMINEKVLPCVSNSCLGDKLMPSHDKMRSSHDKMMPSHDKMMPSHDKLMSPHYTLMSSHDKPVAPSGVSSLGEKKSKDEKKNRKKYNEIYQLSIKKYIYKAGDDLRQDHLVIQIIYVMDNIWKRYGLDLKMTLYRVLALSTDDGFIEFVDYAESISSIKKNYKGEIRQYFIDNSTCSSSPLGFDTEILQNFISSCAGYSVITYILGIGDRHLDNLMVTKDGRFFHIDFGYIFGEDPKPFSPPMKLCKEMIEAMGGAHSIGYEQFLKKCCLAYKYLRYHSQLIISLLDAMCDAGLKDMKMSPELCVLKVQEKFRLDLNDEAAEIYFLSVINASVKTLFPVVVDKLHEWALNWK

>AVK70294.1 phosphatidylinositol-3-kinase [Plasmodium vivax]

MKPLTPQQRKMSSANVKDVDDYFSVRLSLFTVFKGRHGSGVRGRRRRGHGRTERAVAPGEAASSPPDEEANEADSSNAANPADAADAANGGDKTALLRKMRRLEKFKYLQRKRKKKHLMSVSADGKERVLPGRRDSKRRVPPVKSKGATARVRAGLTSGVDYKLNRKRREAPDGKDGKYGKYADEEKIPRDEFEAICYLLMDREFYGHPVSIPCGGLDRRGSHSANQRSDDCMNRFNHLGEGLGKPARVKKDARKSYGDRIAEDLAQMVRASDRSGSAAGGKRKTAKREMADRETGEGEPFCEKPVGGNCPSSGGEPPAGAAAKGESSSGGHSDSHSDGHSDHSPTMPRNRKSEAIRNHLHTINRTLSYPIRHSQLSPDTYLFFLIQHREKKHRTFYAYCRVFSRTGVLKQGLQIKKLFHLGRKARIQDIITSALRKKIAYVDDDSCGRGTSHQGIISFLQENCVYRDLLCLYRLPRKGGFPIGGGSLENARSGASPPRSAKRHGQLICPIEGGPPPHRRRSDAFYQSMKRCSRIERYVRSVRKIYFGWGSPVGNYVHAGDHREVKEGNAKGGRHCGLTWGETPIREQQGKHNSCVSFGEANLGEKRKKKIMTSQVKRLRRKVRRYEGELFCAGGRASHECAQPGHPRRPRHPGYPLEECTPKRSRVAATLRRISEMYAQWKTKEERHIAGFLFFHFFFFNKRCVYYGDKRVGGNEVGRNEVGRDEVGRDRVGSDLGESCPSSCPPDERSSWFFNSFDYVGEAGGASSVARAFLKRVKRQRGEAAQYGEEAPRGVSPQNASPHNASPPRKEKRRNDIFEHMNRYASRISKNIHLANRSRHDDYPFDFSLQKGEGGWHDSIDTAPAMEEIKTITTILNTPVIKLSEAEKKCLWKFRLQLVNREEALGKFLKSVNWDDQQEKEEATELLNCWSKPCLENCLELLNGHLHRAVIKKYVMQIIEQAKKDQLKLYLFQLVQSLRVFNHEPIDSLFMDTLINKCITSKKLSIFLHWFLLSETKDKCKGNLYIHIHKLFITKLMTSNLRKKKKILSILKNQNRFRNQLLYLTKIAKRKSERIHNKTKKLRQFLFCYRQNYGCVVIKDFIKNNIFLSDSEVYDFASPQPRPGAPPGGGEAGEVEGEEGGEVEAEEAEAEGETPDVRNSVYYLNGELSIRADPAADAYCFQYEPRGAHFSQGAAPPAQRSTSDVSTEDSKTVNYIDDSRGAPIERNRDTSFFSNLLQLNDNFDFFLSATYSDEDNHIDILDDSISLVRKQKIKKIRAPLILPIDPDIEFLSFLPEQSYVLRSSLYPIVIACLVRKKIKLAHEHFHNLIINEQKYLKKNVKKKKNYSMYHEVNSKFLKSLYSSFDCASDFERHYRKVKWEGKNIFYNGRKVERVGLQNWGPRDGEESAKVAPTEVAPSNAASAEAAPPSAGVVIHHESETNPQSKQRVKKYNEIYELSIKRYIYKAGDDLRQDHLVIQIICIIDNIWKRYGLDLKLTLYKVLALSTDDGFIEFVNYAESISSIKKNYKGEIRQYFIEMSTDSKSPLGFDAIILDNFISSCAGYSVITYLLGIGDRHLDNLMVSRDGCFFHIDFGYIFGEDPKPFSPPMKLCKEMIEAMGGAHSVGYEQFLKKCCLAYKYLRYHSKLIISLLDCMCESGLKDMKMSPELCVLKVQEKFRLDLNDEAAEVYFLSVINASVKTLFPVVVDKLHEWALNWK

>XP_028862068.1 phosphatidylinositol 3-kinase, putative [Plasmodium malariae]

MKFVKKINDANIIDVDYYFIVKFNFFVLLENEHDGLSDDGKVLHKGSDKILLLEKIKKFKKFNNLQKRKKQKRKKKRRRARKIKVLVIEKNKCRAKNGNTRKKEKRARNDSGEYNHHTTADGEAEDSADVTATATVTAGRDDERTTASKALPRTPKKGAHCNFEIMTYLLIDREFFSYPVRLKCRTINWKKKYNQKSGTKKKKGNFVFSEQRNNTDSSNKFEGDYVNFSSQNDDSPSENEIAKNLAKILHASDKSGMNKKYEEKEQRKQHWHRHWQEEKGEGAMEKTIVERDAREPLNGKPCNGRKGETNKNNTAKYNVDRGCTSNCFSDLDSANNPSSSGELKRNVKRYDSVTTLLTQKRAKVISKNICLINEYVKFPIKYCQLSTKSYIFFIIKNIRNDDMVYYSYCSLFSHEDILKQGVQIKKLYHVKRKKKKIIEYITNALKKKIYCANENSLINITNEQMYTFLKKNLLYHDLLKLYNFEAKNMRSSTNRSSSMHRSSSMNRSSSMNRSSSMNRSSNMNRSSIMNRSSSMHRSSSMNRSSSINRSSSMNRSSSMNRSSSMNRSSRMNRSSSTRRITFMERHLQGTNETSASNKIKNADKLAQEMNKKKNVNVINNKSLKSYSKKILHIAKIFKWNRHNKENIDYGKCTHMSVHSREGTKISGDDQKEEEKGFASDRNRNKTSNANRNNDAYVVAKKKKKKKKKDVHKDMHKDIHEEEGMNEDAHADNREDNDTRGCCSANGWSSKRTRMGSFYKALLRYKQVNNFIFSMERRYRNEKGKNIFFNKTSAEDYVRGRNNVDGQEEINCYVESNIKKLYQDSKKKKNIIYNNDTYDLNFCLVGTKKKRKTKKDKYVRTKIWKRKLLKYEKDLFCVRNFNYMENCYGKDILPDESNDPLQCCKELIELYKKWKIYQKKKISGYIIFNLRSFNKDKIYYNEKKKELSSFHEHILGSTECSQDESYNHNYNSTSHYEWFFNSFEYVGKVEENTNMLSDKFFKRIGWNKNKNSQMLIKPEFGKNYAIEGMVDDYPFRRMLSVEMDEEVGRGSGVNLTTYEMEKIRKNEDSTNLKGTTHINDLTISYHVNNCTEEYTQWEDKIVIHSNSSIKNEDIIYSKKTSTGYSDNKKSAPINDTANSGNAYDFIAQQKGMEEVNTMNLVNQGNNTESVYNVVDVDDVYIVDHACCADEKRKGLIGKGELSADMEFTPCMRKVLPESKEKDVKLNQIGLDEGRVEESRIDNSELDLSNVNESGLDLSNVNESGLDLGNVNESGLDLGNVNESGLDLGNVNESGLDMGNVNERGVDLGRVHRSGLKQMALNDIFEHISKYTSRISKNIRITNRNRYDDYPYDFLWTEKFEKQSLLAPTINEIRTLNTVLNIPIMKMNENEKSCVWKYRFHLLNRKETLGKFLKCVNWNNKKEEEEAIILLSKWSKPCLENCLELFHSYMHYTVIKKYIMNIINNTKKDQLKLYLFQLVQCLRTFNHEHIDDLFINTLIHKCIKSKKLSIYLHWLLLSETKDKSKGNLYIHIHKLFINKLMTSSLKKKKEILEILKNQNRFRNQLLYLTKIAKNKADRIQNKTKKLRHFLFYYRKNYGYINIKQFIQNNIFVTDQNVYDYAHMNEYAHVDDCVHMSNNERDASTFKENENAHSCRISTAVNDHHSIQRGKKEKRKSQTEEEAKNDQDIGNSTPRHTSEDDKENALRADAPVGKCLMVDSTRESAHCDANSNANERSGKTNEGSSKTNEGSSKTNEGSSKTNERSDKTNERSGKTNERSGKTNERSSKTNERSGKTNERRGKSNERNSKTNERSGKSNERSRDVTRAEKPNAQHSIKKDSNNKEMFFENANNKHNEMRNSIYYLTNELSINYDVSKDIYNFQYQKCGNHKSTSDVCDESNKTINYIDDSKNIRIEINRDNSFLSNFLQFSDNFDFFLSTTYSDDDTNIEILDDSISIVQKQRIKKINVPLILPIDPDSELLTFLPEQSYVVRSSLYPIVIACLVRKKIKLSHESFHNLIINKQKYLKKNIKKKKNYSLYHSCNSKFIKAMYSSFDHARNFKYYYKYLRYHNDNIFYKHKKIEKIYMQNTALCHSESSNKKKNLPSGNSSISNRGSKNCGEGFRQTLNGESSEHVKKHNRKAVEEKAVEEKAVEEKAVEEKAVEEKAVEEKAVEEKAVEEKAVEEKAVEEIAADEITADEITADEKENSVSKKCVKKYNEIYELSIKKYIYKAGDDLRQDHLVIQIIYIIDNIWKRYGLNLKLTLYRVLALSTDDGFIEFVDYAESISSIKRNYKGEIRQYFIDNSEETDSPLGFDVQILENFISSCAGYSVITYILGIGDRHLDNLMVSKDGCFFHIDFGYIFGEDPKPFSPPMKLCKEMIEAMGGAHSSGYEQFIKKCCLAYKYLRYHSKLIISLLDSMCESGLKDMKMSPELCVLKVQEKFRLDLNDEAAEIYFLSVINASVKTLFPVVVDKLHEWALNWK

>SBT36335.1 phosphatidylinositol 3-kinase, putative [Plasmodium ovale wallikeri]

MHINGKISTANIKDVDYYFSVKLNLFVFLEVGDGPNMEKFTTEEDTTEKFTTEKDTTEKFTTEEDTTEQHAETTQGGPVNHQHKDRKNMLRKIKYLKKFAYRRRKKKKKSMKILLGRNKKRYNEKYGKNPSKKVSIKRLRGRNRNTMRLPVTQGISRNNPKWGYSRSDHSEVPICNPTDEENYKDFDVTIYLLINEEFFGHPVRLQCSSRRGGQKGVARKGAGKGVGKGVVRKRGKDGGRRYFSSSKGSNEDTSHDALNDNEIAEDLQTFLRSRGSSGESEDKGEGKGEHENEDKGEGKCEVKGEVKGGRSARKFFPTRGNNLANKGRHSIRSNVFTIRRTVKFPLKYGHLSAKSYLLFVVQSRINRDVIFYGYCSMFTSNGVLRQGIQVRKLYFPKKKSKGKSLIVNALKRKISHTQENNPEKTNNEKVIAFLRKNCIYWELLKLCYKEKKEVKIEKEGKKQSGKETNTPVNVSLRENLSHGIDINTTLTNHNGTNGKEPNVKKLKMYRRKMLRIASIFGYNNQKERERKTMNNEGENNTSEGKNRIDYLRNCEEKTFLESERKSKELYTSMGRINNIDAGEEGNTLEIKKVERRHHSLRGENFYDVLRRYKRVENFIFSMKRRSVQNCEREKKYVHVKKDATCECMYTDMDGKKEMGMEMEMEMKMKMELEIGRKSHHCTRKKRKKLFGFTYKRENPLQLCRHVVDLYKKWKMCKKKKIAGYMIFHFHTFNKDEIYYNEKKENVSTFREKVFGAIGCGESEEPTFDHTGRRYPNGGFHDWFFDSFDYVGEMESDALGCNKLFKKMGGMRKCGEAHRERAAATCSESEIREAAASGSEIREAAASGNEIGEVAASGNEIGEVAASRSETREEAANRSEIREEAANSGNNKTVDGDADTCAKVECRRNGEGTSTPPSMQQNGTETVYPTIQKKGKKLNDIFEHISKYTHRISKNVSIEDNNRYDDYPFDLLLKRQFENMPALSPSVSEIKTLNTVLNTPIMKMQEYEKRCLWKYRLHLINRKETLGKFLKCVNWKDKNEKEEAIELLTKWSKPCLENCLELFNAHMHIQVVKKYIINIIRNTKKEQLKLYLFQLVQCLRTFNYEPIDDMFINTLIRKCVKSKKLSIFIHWFLLSETKNKEKGKLYVHIHKLFINTLIKSSLKKKKNILTILKNQNRFRNQLLYLTLIAKNKSDRIQNKTKKLRQFLFYYRQNYGYINIKHFIQNNIFLTDKGICDFSNLTSIDREKNARASQYTPDLLDARCSSTVGNSISGGSTTGDGQCASSRDPEIELDIHESNLHNSIYYLSSELKVNCNVDEGVYNFEYRKRSSENVTSDMWKEPNNIVNYIDDLKSVKIERNRDSSFFSNFLQFNDNFDFFLSSTYNSDEDNNIEILDDSISIVQKQKIKKINTPLILPIDPNTELLTFLPEQSYVLRSSLYPIVIACLVRKKIKLSQEKFHNLIINKQKYLKRNIKKKKNYSLYHSYNSKFVKALNSSFDHANDFEYHCQNVNCKNGNIFYRHRKVERVHVSGASVFPCAVEPLEAVEPVEPVEPVEPVEPVEPVEVDTRIDHSAQGGGSRRGSNTSRRSAKKYNEIYELSIKKYIYKAGDDLRQDHLVIQVIYIIDNIWKRYGLNLKLTLYRVLALSTDDGFIEFVDYAESISSIKKNYRGEIRQYFIHNSVERNTPLGFDAYILENFISSCAGYSVITYILGIGDRHLDNLMVSKDGCFFHIDFGYIFGEDPKPFSPPMKLCKEMIEAMGGAHSLGYEQFLKKCCLAYKYLRYHSKLIIYLLDAMSESGLKDMKMNPEVCVQKVQEKFRLDLNDEAAEIYFLSVINASVKTLFPVVVDKLHEWALNWK

>9E4V_B Chain B, Phosphatidylinositol 3-kinase catalytic subunit type 3 [Homo sapiens]

MGHHHHHHHHHHAATRDQLNIIVSYPPTKQLTYEEQDLVWKFRYYLTNQEKALTKFLKCVNWDLPQEAKQALELLGKWKPMDVEDSLELLSSHYTNPTVRRYAVARLRQADDEDLLMYLLQLVQALKYENFDDIKNGLEPTKKDSQSSVSENVSNSGINSAEIDSSQIITSPLPSVSSPPPASKTKEVPDGENLEQDLCTFLISRACKNSTLANYLYWYVIVECEDQDTQQRDPKTHEMYLNVMRRFSQALLKGDKSVRVMRSLLAAQQTFVDRLVHLMKAVQRESGNRKKKNERLQALLGDNEKMNLSDVELIPLPLEPQVKIRGIIPETATLFKSALMPAQLFFKTEDGGKYPVIFKHGDDLRQDQLILQIISLMDKLLRKENLDLKLTPYKVLATSTKHGFMQFIQSVPVAEVLDTEGSIQNFFRKYAPSENGPNGISAEVMDTYVKSCAGYCVITYILGVGDRHLDNLLLTKTGKLFHIDFGYILGRDPKPLPPPMKLNKEMVEGMGGTQSEQYQEFRKQCYTAFLHLRRYSNLILNLFSLMVDANIPDIALEPDKTVKKVQDKFRLDLSDEEAVHYMQSLIDESVHALF

**10. CDP-diacylglycerol--inositol 3-phosphatidyltransferase [EC:2.7.8.11]**

>XP_038970009.1 CDP-diacylglycerol--inositol 3-phosphatidyltransferase [Plasmodium knowlesi strain H]

MKNKNVYFYIPNIIGYIRVILALWGFMICRKNLILFGFLYGTSQILDAFDGWTARKFNQTSVFGQILDQITDRLSTTLLYLLNGNVYDEYIIAIGLIMIADIGGHYFHSSSCAIAGNKTHKKIEKGNRLLKLYYERPVVMVICIIAYESFWFAAYMFKVTPKNDILHIIAHYALLFSSPLAAFKMFTNISQGIHGVKCLVDMDNKKK

>XP_002809014.1 CDP-diacylglycerol--inositol 3-phosphatidyltransferase [Plasmodium falciparum 3D7]

MKMKKKNVYLYIPNIIGYIRVILALLGFIISRKNLFLFVCFYSVSQVLDALDGWTARKFNQTSVFGQILDQITDRLSTSLLYLLNSSVYEEYITLIGLIMIADIAGHYFHSTSCAIAGNKTHKKIEKGNKLLKLYYEKPWVMVICIIAYESFLICAYLLRVAVKKSLIYKLSYYALICSFPLAAFKMFTNVSQGVHGVKCLVDMDCKKK

>XP_001617084.1 phosphatidylinositol synthase, putative [Plasmodium vivax]

MKKRSVYLYIPNIIGYIRVILALWGFVVCRKNLILFAVLYGTSQILDAFDGWTARKFNQTSVFGQILDQITDRLSTTLLYLLNGNVYDEYIIAIGLIMIADIGGHYFHSSSCAIAGNKTHKKIEKGNRLLKLYYERPVVMVICIIAYESFWFAAYVFKVTPKNNILHKMAHYALLCSSPLAAFKMFTNISQGIHGAKCLVDMDNKKK

>XP_028864224.1 phosphatidylinositol synthase, putative [Plasmodium malariae]

MKQKNVYLYIPNIIGYIRVLLLLAGFVIRQENVLLFAIFYAISQILDSLDGFTARKFNQTSVFGQILDQITDRLSTSLLYLLNASVYDEYITIIGLIMIADIGGHYIHATSCAIEGNKTHKKIEKGNMLLKLYYEKPTVMVICIIAYETFWVSAYILKVADKRQFIYKIGYYMFIFSCPLAAFKTFTNLSQGIHGVKSLVEMDNKRG

>SCQ16798.1 phosphatidylinositol synthase, putative [Plasmodium ovale]

MKRENVYLYIPNIIGYIRVILLLGGFMICQKNVILFASFYMISQVLDALDGWTARKFNQTSLFGQILDQITDRLSTSLLYLLNGNVYNEYIIPIGLIMIADIGGHYIHATSCAIAGNKTHKKIEKGNKLLKLYYERPSVMVMCIIAYESFWVSAYILKVSGKKLLIYKIGYYMFMCSLPLAAFKMFTNISQGIYGAKCLVEMDCRKK

**11. Diacylglycerol kinase (ATP) [EC:2.7.1.107]**

>XP_002258584.2 diacylglycerol kinase, putative [Plasmodium knowlesi strain H]

MEDFAYVDKNVYINLLVTIINKCKEYLLGVGIFKLVFCLCIVVISIIFARRKNKKKKKVNIYIQINNVRHGNDKKTGEIGEDHKKCWAANEDAQIVEPTFPEAREPTNVEQLNNEFYHITYSPHIFDLKSINRTELCNVCNESIYSFIFYKKDIFECVVCRNKCHIECAPNSNLMSCKTAVFFKNKHKFIRIRNCMWNNKCNICSKKFSYFSFPPFAKQYIYKCIWCNKYFHVHCIAKIAKKKKQIHDKKKQVKDAVCNYGNNKYVLLPYEVAIKENVLLDFLMNAYHRVNETQMGNDELLLSYTPHALDDTTGDDGTDELSPQERNEKEKKLDHAKGGKLPLIGNHTPTEQLCLDYVNHFSMFNKMRKFKSFPNSSKAKNNIISVHENFLLNFFPIHLPIYEIKSSRKILLIFLNVKSGGQVGKKLYQELLMYFNPLQIISIKSEKNVLNALNMYKEMIWLNRVIILLCGGDGTISIFIDTLLKFFANEVAMGALQGKNKGKYSELYYGSDKKNEQFLSTKNTFLNKTIMNITAKLKHNKDALRKKWANNITAQAKKPTTFFLKKRTEKNATGQDANNSGDHCRDDEGNISEENAETYFGNESCTAHVWGENQSDQLAPNLTSKVKGAFAGDRRIDDPAALNGELKGDLISQLSGEIIDDLRQHLSNDLNIDSNSWSSGRNTPKDMDKGGTEAKCGNGKYLYILEKLKNFKKKGPEKDDDAENEGELCEDEAYKGSTVKSFQNGNSMMDKYYNGNTVIYSNCTQNNEDPYFESRRGKENEKMYDNILYHDYKENSSSYLPMKTKLNENSPSYCHEANGAIPKKKEENKQHFGNSTSLSYSNGNSLNSGNAECDDGTQVYLCNEDNVTPHQGGYPGGYPGGYPGGYQDNYQRDVKGKTSLTDGIPNMEAGDPNRSDEDVIFQIDNGYKVIRSNEKKKNRQGDGQRSCEQKEITNKIIKREDKEKKSNKNSLESYIACAPIGILPLGTGNDLSYSLGWGCGYNNDPLVYLNKMKNAKNKYIDVWNMKAYDLNNNIILNNSFINYFDFGVISRLALHFDNIRKKFPHFFNSRIGNKILYGEVGFRDFCFNTYKYKLNKNIKLYCDGKKVKIDEDIESVCLINIPYFLGGVKIWKDDDDDNTDKHYHSDMDRQKQEHVRRRRTSKKEMKEQVDENNSTNSSSNSSFIACSMNDNLQNEKNGRIFSNSFGEEKIGHTFDDHKKMAMAEFIKSATLKNSPGEDVNPSEFFQNEKTNGITSPSQGDGVNHPQQEEKSSPPTNEQSFDYSNIYKSYRLDFYRQKKWKQKYRKQKMDDKVIEVIGFRNMFHILQVQIGMSSAIKLCQGSDIKVKIDKTFIQNKNKIYFQYDGEPGFLNIHKLHFTHKCQYLFLSPKDPI

>XP_001352172.2 diacylglycerol kinase, putative [Plasmodium falciparum 3D7]

MEDFAYIDKNVYINILLVFIKKIKDYIVGIGILKILFCVCIILLSIFIAKRKNKKKKKIHVYLQLNRKKNNSTKVSNSSKKSTISNEDEISQKNLSKISDNGKLEEISNGYYHLTYTSHIFNLKTINRIELCNVCEENIYSFFFHKKNIFECIMCRNKCHIECAPNSNLMSCKTSVFFKNKHKFIKLRNCSWNDKCDICDQKFYFFSLYTLLKRHIYKCIWCNKYFHLRCLIKNSYKKKKIIKEDENVVDTQKMKNLCTYGNNEYILYPYQLTIKENVLIDFLISAYNKVQENDIKLDDIFLSYTLNDFYNLEKKFKKDMIEPIEHFPSYKFRSVNDLLYFDYVNHFKSFRNIKKKKKKKIIQIHLNFLLNFFPVHLPIYHIKSNKTFLLIFVNVKSGGQTGKNLYQELLMYFNPIQIINIKNEKNVLNALNMFKQMFYLKKIILLICGGDGTISIFIDTLIKFFLKQNSNNENKEQVQKNEQVNKEPILDNKNKTTSSNKTTFLNKTLTNITEKIRYNKDLLKKKWGGGTPTSQANNNTNNHMNTQGGKLSSKFFLIKRKMKENKKNNDNNDNDKYSTSEEDTSDFSDTDDEDGDEEEKFSSIKEQLFKTKVNEDTLNVTENYEHIYRQIKNFQNKDIHEKDDAYEDITEENKNKIEQNHKKKEKKKDNNKNNSSIMNDHGSDINEDCYFSATGFEGESIYHNVYYDHNNKNSNDLHMTTNISDNLCGYYGDTKKKKLKYIVKGDNNNGDSTMVSVPSNNKKKKNNNNNNNKTTITTTTTTTITNNNDNNNDNNNDNNNDNNNDNNNDNNHNDNHNNNHNNNHNNNNNTNSSHNNSSIIINNEDLHNMTDKKNEGLNKINNIEEFNKGEMCMFEKQCNILENINKDYKLLNTGYDEKSHIDDETFFDSCDKSKDDIQCTHSNLILKELSEETNLEKDNEEDNKYSLESCISTTPISILPLGTGNDLSISLGWGSEYNNDIFFYLNKLKHCKNELVDVWNMKGYDSNNNLILNNSFINYFDIGIIARLALHFDNIRKKYPHFFNSRIGNKILYGEVGFRDFCFNTYKYKLNKNIKIYCDGKKVNIEENLESVCIINIPYFLGGIKIWKDDELEKNYYSDFEKENNKMNDSEYISDHEKADHHHHNNKKKKKKKKKTKCKIKNNDSIINKDEKYNTDNINDNSFESYSSGTNIYNRYDNMYKKLYTDQDKQNIMNNISLKKDLLINQTNINSPEIKNIEKFINRNENASDYANNIYKSYRLDSYKQKKRQHKYRKQKINDKVIEVIGFRNIFHLFQVQIGMSKAIKLCQGSEIVVKINKKFIKNKNKIYFQYDGEPGYLNIHKLHFTHKCQCLFLSPKDAI

>SCO66633.1 diacylglycerol kinase, putative [Plasmodium vivax]

MEDFAYVDKNVYINLLLTILNKCKEYLLGIGILKLLFCLCIVLISIIFARRKNKKKKKVNIYIQLHNVRHGNGKKAAELADGQKCYSTNEEAQIVEPTFPQTPEQTDVEQVNDDFYHITYSPHIFDLKSINSTELCNVCNESIYSFIFYKKDIFECVVCRNKCHIECAPNSNLMSCKTTVFFKNKHKFIKIRNCMWNNKCNICSKTFSYFSIPPFVKQYIYKCIWCNKYFHVHCVEKAAKRKEQTKGSKKQVKDAVCTYGNNKYLLLPYEVAIKEHVLLDFLTNAYHRVKEAEVGNDEVLLSYTAQALDDTLRGDATGEFNTHGGNANKGSANGGSANGGSANGGGKHTPEEQLCPDYVNHFHTFHKMRTFKSFPSCSKAKSGVTPVHANFLLNFFPIHLPIYEIKSSRRILLIFVNVKSGGQAGKKLYQELLMYFNPLQIISIKSEKNVLNALNMYKEMIFLNRVIILICGGDGTISIFIDTLLKFFANEVAISAQQGKNKGKYNKLYWSDKKSEQFVSTKNTFLNKTIMNITAKLKHNKDALRKKWANNITAQAKKPTNFFLKKRSEKGEKNADGEEADRCGDDDDGDDDAGHISEENAETYFGNDSSTARAWGESQSDELAPKQASKAKAALSGELNGELNGELNGELNGELNGELNGELNGDFSADLSADLRHHLNSEANGWSSGRSSPKDLARRGTEAKGANGKYLYLFEKLRNFKKKGAEKDEGGENGDDLCEDEAYKGSTVKSFQNGNSMMDKYYNSNTLIHSNYTENNEDPFFESRRGKENEKMFDNILYHDYKEKSTSYLPMKTKLNENSPSYCQEVSGANPSDEPDGTAGVNAKKAHNKHHCANDTRSNYSNGNSLNSGTADCDGGSADRGGGTPVHLRNGDDVAPHRGGAYQRNVNGETAKTEGLSKLEAANQDGSDEDIVVQLDNGYKVVHSKGEEKNRHGEHRHGEHPHGEPPHGERKDKEKRLHKNSLESYIACAPIGVLPLGTGNDLSYSLGWGCGYGNDPLIYFNRMNSTKNEHVDVWNVKAYDLDNNIILNNSFINYFDFGIISRLALHFDNIRKKFPHFFNSRIGNKILYGEVGFRDFCFNTYKYKLNKNIKLYCDGKKVKIDEDIESVCLINIPYFLGGLKIWKEDDKDKNYHSDVDRQKREHVGRRPSKQEEKKQGKEQPEGNKSTNSSSNSSFIGCPMNDHLPHQKSGGILSNRFGDRNGHPFDDHKKMAMTEFLKSAALKHSAGEEVNPSEFFQNEETNETTLHSEGENHPQGEENPSSPPQTNEQPFDYSNIYKSYRLDFYRQKKRKQKYRKQKMDDKVIEVIGFRNMFHIFQVQIGMSSAIKLCQGSDIKVKIDKAFIQNKNKIYFQYDGEPGFRNIHKLHFSHKCQYLFLSPKDPI

>XP_028860891.1 diacylglycerol kinase, putative [Plasmodium malariae]

MEDFAYVDKNVYINVLMLLLKKTKEYFIGIGILKLLFCVCIICISIFFAKRKNKKKRKINIYLQLNESGCTNGEDVVVLDYNKRSTTKEEAGLKERSSKLCEYVKVHEKKNHFYRLSYSSHVFDLRTINRIELCNVCNENIYAFIFFKKNIFECIMCRNKCHIECAPNCNLMSCKTSVFFKNKHKFIKVRNNLWNNKCSICNKKFSFFSLYIFIKRYIYKCIWCNKCFHVKCVNKHFSKRRIKDAKNEHKNIVCTYNNNKYLLYPYEVFIKENVLIDFLIHAYNKVTEEETKKDDILFSYNSLNNYGMDQIDNKKKIVEKREDMMMMPHKTLAKDTKELLYFDYVINPLKKLKRYKNVNCKKKNIIIPVHINFILNFFPIHLPIYQVRSSKKFFLIFVNVKSGGQTGKNLYQELLMHFNPIQIISIRNQKNVLNALNMYKEMFYLNKVILLLCGGDGTISIFVDTLIKFFSSQLVYDSKKEKKKNTHVGNTNENIFFIRGLFLNKTIKNIKEKLKHNKDMLRKKWNNKGNTQMNKDSNIFSIRESEEKTIKTDDSTMNERRTEDVLLFRDHPRAHKGEHLTISGECVGSRSAYYSPAAPLVSSTSSAHAAFSAVNYFHSDVHNNDIIDEVGRGTPNAGANMDINTDLNSDMNSDMNSGMNSGMNCGINRGINSGMNSEVISGLNSDLSIDLKNDLNNLDKWKKRNKYNYILKKLKKFKKKSTKEEDICRESTIKSFPNGNSMTDNCKSSNPLIYRKKEENNGDSYLSTKGVENERMYKNILYHDYKEKTASTCFSTKTGLNETSVSYYREGEEIFSAYINNVNGTNNCNRCYNSICDKCRVNKDDHLGNENNNKGNNHNGKENVKNASEQRKYIKKNKNKTKESLHIKEVPYANDSDNNSVYKIDNGYKIIVRKRDGETRVVGDQYNCSKKEVVCSTQTEEGKGQGLELELGQEIKLGEEVKLVKEKKGEKRKKKKLSLESCISNTPIGILPLGTGNDLSISLGWGSGYNNGLFFYLNKIKNSKNELVDIWNIKGYDLDKNIILNNSFINYFDIGIISRLALHFDNIRKKFPHFFNSRIGNKILYGEIGFRDFFFNTYKYKLNENIKIYCDGKKINIDEDLESVCLINIPHFLGGVKIWKEDELEKNYYSDVEREQQHRGEGEEEDGNEDVDVDIIDDIDTDGDIDMHDKVDKKKKAKLKLSSNKVSSLGCPKHIHDYDSFSYNDDVRAERHSRSCHPKLNKNIDNSYRAGKKGVTKRCAYKYYDKHEYALPELKKNYESIKKQEKKSEDNEESPKNDKPLDCSNIYKSYRLDFYKQKKGQQKFRKQRTDDKVIEVIGFRNMFHLVQVQIGMSKAIKLCQGSDIVVKIDKKFIESKNKMYFQYDGEPAFLNIHKLHFTHKCQCLFLSPKTPI

>SBT76497.1 diacylglycerol kinase, putative [Plasmodium ovale]

MEDFAYVDKNVYINIVMTILQKLKEYLLCIGIFKLLFCLCIILLSIIFAKRKNKKKKKINIYLQLNGGKYGNKEVEKGEVMCSMKKSLLTNENVERSGKKCLSNASEDTEIEEQKNVFYHISYSPHIFDLKIVSRTELCNVCNENIYAFIFYKKNIFECVVCRNKCHIECSPNSNLMSCKSCVFFKNKHKFIKLRNYLWTNKCDICEKKFSFFSSSIFVKQYIYKCIWCNKYFHVKCVTKYCAKNKNKNEKNKEKDILCTYGNNEYILYPYEVYIKENALITFLLHAYNAAEESQIKRDDIFLSYTIKQLNNSKVQNEREKVSEYEPDEASSKRRSANELLYFDYLHRFHRFQKCQKYQQQQQHQQHRQHQQQHQQQQKCQKFHNSRKLQNGKKFQGRKKSHQGELQKGTVQPENFPNAQVSPRTKNKIRYVAPVHANFLLNFFPMHLPIYEIKSSKKILLIFVNVKSGGQAGKNLYRKLLMYFNPLQVISIKSEENVLNALNIYKEMLYLQKVILLLCGGDGTISIFIDTLIKFFAKQAPFETQKGKNMKEQAGERNTTKEKKHTLSSKGTTFLNKTIMSITAKIKNNKNTLTNKWIKKGKNGKDTSSKLSHTKREIAAIDHGNSNYTENTETCLDNCAYSYIEENNNLHLHLHAHDELEGNEKGENLYRSSIHDIQSGDNNNANCDANTNIDSDNEKDVDGDIGNGACECPDSDEWCSASSDDHIVEGGKNNCKYKYFAKKMKELKKLKELKKLKELKELKELKKNDAEEYTHRESTLKSFHICNSHTDKYNDSNMLTHTNSTKNNEDPYFSSRGIENEKMYNNILYHDYKERVSNYVPMETRLHDNAVSYCKGVDQHKDYPDDHGTGEDTYNVGTRDDRPGCSGIYVSGESSGGRSGSGGYGSGAYESDGSIHKIDNGYRVIRRKEGENYTNLEKKNYQSEFMKSFEKCRKRVSDVEPPTAEQPIVEPPIAEPPIVEPPIVEPPIVEPPIAEPPIVEPPIAEPPTARANNEKGTVESLILSTPISILPLGTGNDLSISIGWGNEYNSNSLFLYIDKLKESKNELIDIWNIKGYDINNKIILNNNFINYFDIGIISRLALHFDNIRKKYPHFFNSRFGNKMLYGEVGFRDFCFNTYKYKLNKNVKLYCDGKKIKMNDDLESICLINIPYFLGGLKIWKEDKFEHYPYTDVKNDYEHFMQEYFYLNKKEHEGKNENIEQAYKCLNRADAAIPANAANHANAAIPANAAIPANYNILRSSSESSSSIYSMNDKHNICCDNVESNDWKNEQVHRREDISYKCTCNHTSNPTIPCKFHNKNYSNDKKNYLGQENRNKCNYNIYKSYRFDFYTHNNGKQMFRKQEFNDKIIEVIGFRNIFHLFQVQIGMSRAIKLCQGSDIVVKISKQFIQKKNKMYFQYDGEPAFLNIRKLHFTHKCQCLFLSPKDPI

>XP_016867281.1 diacylglycerol kinase beta isoform X5 [Homo sapiens]

MTNQEKWAHLSPSEFSQLQKYAEYSTKKLKDVLEEFHGNGVLAKYNPEGKQDILNQTIDFEGFKLFMKTFLEAELPDDFTAHLFMSFSNKFPHSSPMVKSKPALLSGGLRMNKGAITPPRTTSPANTCSPEVIHLKDIVCYLSLLERGRPEDKLEFMFRLYDTDGNGFLDSSELENIISQMMHVAEYLEWDVTELNPILHEMMEEIDYDHDGTVSLEEWIQGGMTTIPLLVLLGLENNVKDDGQHVWRLKHFNKPAYCNLCLNMLIGVGKQGLCCSFCKYTVHERCVARAPPSCIKTYVKSKRNTDVMHHYWVEGNCPTKCDKCHKTVKCYQGLTGLHCVWCQITLHNKCASHLKPECDCGPLKDHILPPTTICPVVLQTLPTSGVSVPEERQSTVKKEKSGSQQPNKVIDKNKMQRANSVTVDGQGLQVTPVPGTHPLLVFVNPKSGGKQGERIYRKFQYLLNPRQVYSLSGNGPMPGLNFFRDVPDFRVLACGGDGTVGWVLDCIEKANVGKHPPVAILPLGTGNDLARCLRWGGGYEGENLMKILKDIENSTEIMLDRWKFEVIPNDKDEKGDPVPYSIINNYFSIGVDASIAHRFHIMREKHPEKFNSRIN

**12. Calmodulin, putative**

>XP_002260670.1 calmodulin, putative [Plasmodium knowlesi strain H]

MADKLTEEQISEFKEAFSLFDKDGDGTITTKELGTVMRSLGQNPTEAELQDMINEIDTDGNGTIDFPEFLTLMARKMKDTDTEEELIEAFRVFDRDGDGYISADELRHVMTNLGEKLTNEEVDEMIREADIDGDGQINYEEFVKMMIAK

>XP_001348497.1 calmodulin [Plasmodium falciparum 3D7]

MADKLTEEQISEFKEAFSLFDKDGDGTITTKELGTVMRSLGQNPTEAELQDMINEIDTDGNGTIDFPEFLTLMARKLKDTDTEEELIEAFRVFDRDGDGYISADELRHVMTNLGEKLTNEEVDEMIREADIDGDGQINYEEFVKMMIAK

>XP_001616632.1 calmodulin, putative [Plasmodium vivax]

MADKLTEEQISEFKEAFSLFDKDGDGTITTKELGTVMRSLGQNPTEAELQDMINEIDTDGNGTIDFPEFLTLMARKMKDTDTEEELIEAFRVFDRDGDGYISADELRHVMTNLGEKLTNEEVDEMIREADIDGDGQINYEEFVKMMIAK

>XP_028863647.1 calmodulin, putative [Plasmodium malariae]

MADKLTEEQISEFKEAFSLFDKDGDGTITTKELGTVMRSLGQNPTEAELQDMINEIDTDGNGTIDFPEFLTLMARKLKDTDTEEELIEAFRVFDRDGDGYISADELRHVMTNLGEKLTNEEVDEMIREADIDGDGQINYEEFVKMMIAK

>SBT78743.1 calmodulin, putative [Plasmodium ovale]

MADKLTEEQISEFKEAFSLFDKDGDGTITTKELGTVMRSLGQNPTEAELQDMINEIDTDGNGTIDFPEFLTLMARKMKDTDTEEELIEAFRVFDRDGDGYISADELRHVMTNLGEKLTNEEVDEMIREADIDGDGQINYEEFVKMMIAK

>3UCT_A Chain A, Calmodulin [Homo sapiens]

ADQLTEEQIAEFKEAFSLFDKDGDGTITTKELGTVMRSLGQNPTEAELQDMINEVDADGNGTIDFPEFLTMMARKMKDT

**13. Phosphatidate cytidylyltransferase [EC:2.7.7.41]; Cytidine diphosphate-diacylglycerol synthase**

>XP_002260899.1 cytidine diphosphate-diacylglycerol synthase [Plasmodium knowlesi strain H]

MPKRNEQVKENSGPISDSSFGSNSEDDNSKIIGNGVEEKKKKNKLTNGDINVGQKKKKEAINRNNHVENDQKEAHNSDNYDEEDKTNITNDDNYSEEDKTVDRTRSKSRSLDTNNECADDIDKLRNGNNLTYKKKKSTDEINGKVENWEDQNMMKNNQKIEYRRRKSSSKEIKCYKDLKHLKEYANVKSTNNRFTNFIRFYGYRGIPNNGSKNKLSISSQNSTSHNINLKNHEWNLDTFKTRCISSIILIFLCSLTVAAGHFYCSILVLILVSFVYREIISLKSVENKDKKLPEIFYIRWYWFFLTILTWGIPWIIPKLKHQIGLFKYMLKYHSIIMFISAFFGLIWFILSLRKFSLKYQFSQIGIILLSSLFIVTQSLMHIANIYSGLIWFIVPVTSVAVNDSFAYIFGVLFGKTRLIELSPKKTVEGFVGSSVITVLYGIGATYLLQNYKFFICPQNHISFIPFSTLHTMDCENSSIFKPKYFTLPSQLSSLLSINRIYYTNMVLHGLVLSLFAAFLAPFGGFFASGFKRALKIKDFGHAIPGHGGATDRFDCQVFIGMFTYIYLKTFVKIKGRINYSYDVLIDSIQKLDHKEVLRLFNQLKNMIDKKRKKNGDKKKDHHNKHPSKDDKCNGKKQLLT

>XP_001348270.1 cytidine diphosphate-diacylglycerol synthase [Plasmodium falciparum 3D7]

MPRKNSKTMTNKCANSNSNDFIEDKVLDDKKRKKSTESSTVDEKGDIIKRRSKSKSLDDSNGGIENMDKVKVSNNNRKKKSETMINNDNNNDNDDIKSMNHMNSMTHTNSTNHTNNMDNMDNMDNVNNMDDVNNMNNVNNMKNNINDEVYKLGSFEYEEECKKRNIRNSECKRRRNLKDMKYFKDIKPLKDFVNLKNSNNNNNTNQHNSGIFSNFTRFYGYNNKNSFNSNRSKQSTCSNSNLQNNMNLVNSESFESKLQTFKVRSQWTFILILLYFIILAAGHFYCSILVLILVTTLYKEIISLKSIENKDKKLPEIFYIRWYWFFLTIMTLGIPWVIPKLKHQIPLYKFLLTYHSINMFILAFVGFVWFILSLRKFSLKYQFSQIGIILLSSLFIVTQSLMHIANIYSGMIWFIVPVSSVVINDIFAYVFGILFGKTRLIQLSPKKTVEGYVGSSIITIVWGILITYFLQRYKFFICPQKYITFQPFVSWNYIDCDINPIFQQKVYEVPKQISQILSIKNIYYSKMIFHGLMLSLFAAFLAPFGGFFASGFKRALKIKDFGKSIPGHGGVTDRFDCQIFIGMFTYIYMKTFVKIKGGIYSYDLIIESIQKLDHKEIIRLFNQLKNIVDKKRRKQCTDNNTNDIHTRKDKRSNIDKPCTDKNKIPNK

>KMZ90760.1 cytidine diphosphate-diacylglycerol synthase [Plasmodium vivax Mauritania I]

MPKRNEQVTESSGSISDSSFGSNNEEDNPKIVSDGVKEKKKKKKLTNGDSTVGQNKKKGTINRNNHVGDNKKETHNGDNYAEEDKTNVTNDDNYAEEDKTVGRTRSKGISLETNNECVDDVDKLKNGNNNSYKKKKSTDEINGKGEYGEDQNVTKNNQKVECRRRKSGSKDIKGYKDLKHLKEYANVKSTNNRFTNFIRFYGYKGIPNNGSKNRMSIASQNNTSHNLNMQNHEGNIDTFKVRLISSLILLFFSLLTVAAGHFYCSMLVLVLVSFVYREIISLKSVENKDKKLPEIFYIRWYWFFLTILTWGIPWVIPKLKHQIGFFKYMLKYHSIIMFISAFFGLIWFILSLRKFSLKYQFSQIGIILLSSLFIVTQLLMHIANIYSGLIWFMVPVSSVVVNDTFAYIFGVLFGKTKLIELSPKKTVEGFVGSSVITVLYSIGATYLLQNYKFFVCPQNHISFIPFYTLYTTDCEDSSIFKPKYYTLPSQLSSVLPISRIYYTNMVLHGLVLSLFAAFLAPFGGFFASGFKRALKIKDFGRSIPGHGGATDRFDCQIFIGMFTYIYLKTFVKIKGRINYSYDVLIDSIQKLDHKEVLRLFNQLKNMIDKKRRKTIDRKRDQHIKPPPKDDKCNGNNK

>XP_028863870.1 cytidine diphosphate-diacylglycerol synthase, putative [Plasmodium malariae]

MQDRNEKNRKNSTSTKGSFIENINEEKKKKKKGRKDSRDDITEEKQGPVKRTIKNKYCKKDCTEDDVMIMAPSSPITTHNYNNNDENGNDNNSDKNNNNNSNSNSTNNAGKKKRSTDELIGKNVLTEDLSVNKGDSKNEIKKKKVYKDMKYYKDIKQMKEYINMKNTNSRFTNFMRFYGYYRGAHNSSKYKTLITNQDDTNNNINSYAPKENSKSKLETFKVRFIWSCVILFFCFFILALGHFYLCILVLLSVTVVYNEIVSLKSIENKDKKLPQIFYIRWYWFILTILAWGIPWALPRLNHQFRLFKYLLKYHSINMFILAFWGFVWFILSLRKFSMRYQFSQIGIILLTSLLVVTQSLMHIANIYSGLIWFFIPVSSVVVNDTFAYIFGILFGKTQLIELSPKKTVEGFVGSSIITILWGVFATRCLQHYKYFACPQNNISFIPFYTMFTSDCEDNAIFHQKVYILPTHLSNYLPVDKIYYTKMTVHGLVLSAFAAFLAPFGGFFASGFKRALKIKDFGQTIPGHGGFTDRLDCQIFIGMFTYVYLKSFAKIKSRVHYSYDVLIDSIQKLDHKEIMRLFNQLKNMIDKKRRKTINTKINQEKFASKDPVRNDDEILS

>SBT46179.1 cytidine diphosphate-diacylglycerol synthase, putative [Plasmodium ovale wallikeri]

MQKRNVKNRKGSKSICGSSSTENNEEEKKKKKNSKDTSDIETSKSSVQTRSKSKSLDSQTECAEEVEKLKNYNDIACKKKKNSNGDIYGGGSEVMEEWNNGKNSQKYDCKKRKSLKDMKYNREIKHIREYTNLKNANNKLVNINVSYGNHRGLYNGSKHRTSNSSQGNYNSAVSQENSQTKMQIFKVRLYWSFVIIFFSSFILAMGHFYLSLLVLIAVTVVYREIISLKSIENKDRKLPQIFYIRWYWFFLTILTIGIPWIIPKLKHQISFFNYLLKYHSINMFVLAFVGFVWFILSLRKFSLKYQFSQIGIILITSLFVVTQSLMHIANIYSGLIWFILPVSSVAINDSFAYIFGILFGRTRLIKLSPKKTVEGFLGSSVITILWGIAVTYLLQSYHFFICPQNYISFIPFYTLAKVECEYNSIFQQKVYTLPVEVSTYLPINKIYYTKMVLHALVLSLFAAFLAPFGGFFASGFKRALKIKDFGQAIPGHGGFTDRLDCQVFIGMFTYLYLKTFVKIKGRVNYSYDVLIDSIQTLDNKEVLRLFNQLKNMIDKKRRRNPDKNKDQPRFPIQETTPISDNK

>NP_003809.1 phosphatidate cytidylyltransferase 2 [Homo sapiens]

MTELRQRVAHEPVAPPEDKESESEAKVDGETASDSESRAESAPLPVSADDTPEVLNRALSNLSSRWKNWWVRGILTLAMIAFFFIIIYLGPMVLMIIVMCVQIKCFHEIITIGYNVYHSYDLPWFRTLSWYFLLCVNYFFYGETVTDYFFTLVQREEPLRILSKYHRFISFTLYLIGFCMFVLSLVKKHYRLQFYMFGWTHVTLLIVVTQSHLVIHNLFEGMIWFIVPISCVICNDIMAYMFGFFFGRTPLIKLSPKKTWEGFIGGFFATVVFGLLLSYVMSGYRCFVCPVEYNNDTNSFTVDCEPSDLFRLQEYNIPGVIQSVIGWKTVRMYPFQIHSIALSTFASLIGPFGGFFASGFKRAFKIKDFANTIPGHGGIMDRFDCQYLMATFVNVYIASFIRGPNPSKLIQQFLTLRPDQQLHIFNTLRSHLIDKGMLTSTTEDE

**14. Inositol-hexakisphosphate 5-kinase [EC:2.7.4.21]; Inositol polyphosphate kinase, putative**

>XP_002262058.1 inositol polyphosphate kinase, putative [Plasmodium knowlesi strain H]

MKVEEYRHQVGGHCKLIKPKDSSKVYKPLIENEYIFYEKLANFGASSAESGPLHILKKFIPKFYGVTEIVVEYSSSSEMEDNLVRKQKRRNEPNSSKGRKYFSLEERDKKDKLDEQPSNEPTSQRTDQSAKEPSREPSREPSKEPSEELPKESPQGETEGEKDTKSDKSAKGKKRKKCIPHIVLEDLVYGFKRPCVLDIKMGKRQRKIGASLEKRKRQVEKSFKTTSHSLGFRLCGCQLYNKTSDKLFYKDKYWGRNLTKENIPWAIRNWFWNGSLLYEELIPLLLEKLHRFFNCIMELRHYRFWSSSLLWVFDGGLNDQKARSNSLDIRMIDFANTIYLQDNPSVDDEYIFGLKNLIHSMQILNNTIQGMNFLPQEISTCFYSENYKLIENSHRPIFKKSKSAILEENLRKKKKKKKNVYINFEFLKNAKTRRKSSNVYSSNMTGALATQLAAGSPKGTSDDGLSFQYLNKFIGNNKKEKIWNQAQYFSNSDSPIGNHMSLSPYSTPVGTFINKEVCISGWLDESLPRNVTHGPGVSPDLNIPLIPPTSVDDKNDGSVSAEHSHGSDIQEKANITTFNESEKGPMENELLALEAHQIDDHRRSDHHSGMPSKEDESVSPINLIQDPKGVHLDDEDELVDMPKKTITIIKHNERSEQMSTNNSIDERRNVRFEGKNFAHGHFITAMHETDGRNRHSEENAIEKEEVQTNFSFKNKNRYPVNHSEEETCGVTEKGEIPQSENYQKCVNKSGEKQIIDDKQSRDNALLFQTRENVSFMSAEGDLFGEDNSNDRNESNAYEQVIQEVIVKTLKVASRGRDNFSSVNIANKIPVDRNYKNRIEDERKNGGYRKEGEPRRHENAFTNFYESEGKDPPYKHEYILSNEGNRNEVAENGDEGSDRKVSDAEVEKKEENRRSAKQDLGNKGSYAEKYTQQYAEQYTQGGLNQGMLKKREHRIRSALGPKLRNDSLVRETKWNLLIRNVNLKVFINHMIKKEIEGKKNTQEYPYLFVKNRNETDPRALHMENRPSKIGGLNRISRDSRARGGVPPIHNPYVFEKSLSYSYSHRIAHSFPQNRNYSYSSDSGLIYNRNLQGGHLMKMLKKEGRITSKRCSSCTDIPLRIKKGKKSQKKKKYIKKKLFKKINILSGVKNNRVKSPQKIIFSNYAVPNQHSVTPPIEYTPRSDSICRITHSFDIFTPEYRRNRYVHSVISQRYNTDMDSTTNNQTGNIPFREKNLILPLTDTNSNEKYLRRSMSEPNLYKFGYFRCILNDLNDTKINYNSLVANRLDKMMKVPIYNQIYGFTSNSSDLDSSDTSWGY

>KNG75710.1 inositol hexakisphosphate kinase [Plasmodium falciparum IGH-CR14]

MNVEEYRHQVGGHCKLIKPKDSSKVYKPLIENEYIFYKKLTNFGSSSTESGPLHLLKKFIPKFYGVTEILVESCSDDEEKQNDSSNHIKGKDEKKKKNKSRYKKYIKMDKSEKENFVNTDETYFVREQKYSEQIRMRENISNGDIHENIPKNMSSGELKEHKVNHMKIVDKHNESNMYGDAYGSKNDEMNKNNNEMYKNNNEMYKNNDEMNKNNDEMNKNNNEMNKNNDETNKNNDETNKNNDEMNKNNNEMYKNNDEMNKNNDENVKKRYNENNTNATPKKFKRRKECVPHIILEDLVYGFKRPCVLDIKMGKRQRKIGASIEKKKRQVEKSFKTTSHSLGFRLCGCQHYNKVSDTLFYKDKYWGRNLSKEHIPWAIRNWFWNGSLLYEELIPLLLEKLHSFFNCIVELRHYRFWSSSLLWVFDGGLSDKKARSNSLDIRMIDFANTIYLQDNPSADEEYIFGLRNLIESIQILNNSIHNIYFLPYEITTCFYSENYNMKEIKDRKVLKKSRSVVYEDNKKKKKKTVYINMEFFKKAKGKYGSNNNKQLDNNKLVSNNKHMNNNKHLNIKKNDIKTKYIYNSDLLNTENINKFLSQNEKEEIHKNKKQNRNININKIPKKKPKKLYIKHNKYQSFSDYAIREDIYSTPLFSFTNKLHNSVSNNPHNIKKTHNMNNFGIHCLLNNNSVSTSRVEDNAYMEEIFNKYKNYEYNNVYDKNIGSSHNDRDPLVDTHLYDENNKILYNTCLKENDNIYYKGLENTMDKCMNNTMNNLFHIQNYNYDKQYNNKIKTHHTSNDRTNINNEEILYVNKNISRNNNIYINNNERKKKNILLKKSYNMSMQNKNLPYQIDECNKNKTKEEKVIYNINDNSPIFIDDKYKEDILQNKIYFDKESLENIQENQNYILNDHTDVLKYMNYNMDNKCYDKNININISNNNNNILLTYQNKRKNESISDNNLHTKSDNKYDEIIQETIIKTLKIASNFYQQIKEHINIDQQVKDQEKVPQPCSDDDNIKNNVQQGEDKIKKKEDYLLSYHNVADDHFNNNRCEDNKKDFYSFNFDESQKKKNSIETYKGNINFQTNNKDDNIKIKEKKKNYKKEISNYYFEKGEMENINVFPNEEENVIHEKKNKDIQVFKTNNIHDNNVEKNNINNIKIIKNFNMLKRNINLKVLINQMIKMEIEKRTLEKQQLLLKNKQTKKRINTHTNKQIYKEINTHTNKQTSKKNILKITNNEMTDFCDINVDIIEVQNKIKQMDIKENMLNKMIYRSEKDTLYKSSKNNIYEQLNYNSDTILIHKDFKSCHLKMNNKNNYIQDFNMNKRCMSCNDDIISSLKNDNKIKEKKEKQNKKKIRQLFLKKLSMLSNMKSNKKKYHDNVYYNGDPDNSNEEKNGEYINKYNDKYYNEYDNKYNIQHNDTYDDKYVNTINNIPLRFSKYYLKEKKKKKKYLKCNQLNMIPLHKKKSIYKNEYPENNEEFNIFNMNYEKNMYTFNNIKYTNENLNYINNIYENNEFETYKNLILPLTETNENYKYLRRSLSEPNIYKYNRFMGTQKDSYVNKINYNNLIKNRLDKLTKVPIYNQIYGFTSNSSNTYSSESSLDM

>VUZ98696.1 inositol polyphosphate kinase, putative [Plasmodium vivax]

MKVEEYRHQVGGHCKLIKPKDSSKVYKPLIENEYIFYKKLTNFGASSAESGPLHILKKFIPKFYGVTEIVVEYSSSSEVEDNLVNKQKGRNEPTGNKGRKYFSLDGRNEADQPSNQLKNELPNQLPNQSLAEAPQGEPGIGERDAQSDKCAKGKKRKKCIPHIVLEDLVYGFKRPCVLDIKMGKRQRKIGASLEKRKRQVEKSFRTTSHSLGFRLCGCQHYNKINDKLFYKDKYWGRNLTKENIPWAIRNWFWNGILLYDELIPLLLEKLHRFFNCIVELRHYRFWSSSLLWVFDGGLNDKKARSNSLDIRMIDFANTIYLQDNPSVDDEYIFGLKNLIHSMQILNNTIQGMHFLPQEISTCFYSENCKPRGNSFRPIFKKSKSAILEENFKKKKKKNLYINFEFLKNAKARRKSSNVYSRPMPGGLSTQLEIGSPKGTSDDGLAFQYLNKFMGKKKKKKNWNQAQYFSNSDCPIADHMCLSPCSSPVGRLHNGEICFSGWLDEGLPGNVPHRSRETPHLNIPLLPLTSVDHTDDGPTSGKHSDGSDLQEKANMTTFSESANGLVGNDILELEAPQMGDSLRSDHHTEVPSRGKEPVSPINSNGNPKWAHPAGEDDLVDVPKKTTFTAKQSETEEQMPTNNPIDETKIEGYDAGHIDCDHAIPTKHATDGRNRQTGESTTEKEEVQANSSLKSKNKNCYPVNTSEEDATNGVTQKGEIPQSEDFQKCMNKSGEKQTIDDKLSHSNAPLLQTCDNVSSISGEGNSFGKGNPEERNENNSYEQVIQEVIVKTLKVASRGSEREKSASAGIANKDTVNRSSANRMEDEGKDDGCPDEGDSERHENASAIFCESGGKGRPYKHEYILSNEVDGNEAAQNGDERSDRKGSYPKGSDAEESGAEKSDAEVEENRAGKNNKQGDRKDTKQNLGERGSHTEQHTQGGLNHDMLKKGEHPIGSEHRPKLTNDSLMRETKWNLLSRNINLKVFINHMIKKEIEEKKKAQEYLYILVKGRNEVDLRAVRREHFSDSNREAAGIPRKAQRGPRAGGGRRTIGTSRIGGASRTGGGAGRGKNHASDPPVHNPRLSEKSLSCSYSYSYSHGHRYIRSYPRSYSSDSGLIYNRSLQGGDPKKMLKKEAGITSKRCSSCTDIPLRIKKGKTKKKKKKNIKKKLFKKINILSGMKNNIIKSSPKIIFSNYTVHNHHCVTPPIEYSPKSDSICRITDAFNVFTPEYRSNRYVHSIISQRHSAHQVEDLPNTHIDRAINNQRDYNPFREKNLIVPLTDTNSSEKYLRRSLSEPNFYKFGYFRCILNDFYGTKINYSSLVANRLDKLSRVPIYNQIYGFTSNSSDLDSSDASWGY

>XP_028864229.1 inositol polyphosphate kinase, putative [Plasmodium malariae]

MYVEEYRHQVGGHCRLIKPKDSSKVYKPLIENEYIFYEKLTNFGSSSAESGPLHILKKFIPKFYGVTEIVVESSSSSFSSSSISDLESGSDMNGNQNIEIREKEKNKQSQTSKENERNERNERNERNERNERNERNERNERNEQNERNEQNERNEQNKKQKSKHKYKYIKLSGIEGELIRRDYFEWTQSGRMQEEANDEGVKTNKDGTKTRVEETNVGDVCIRKEDEDSKLLIIPKKKKKKKAENTDTCEKLTNRGKRKKKKKKKKCVPHIVLEDLVYGFKRPCVLDIKMGKRQRKIGASLEKRKRQVEKSFKTTSHSLGFRLCGCQHYNKLKDTLFYKDKYWGRNLSKENIPLAIRNWLWNGTLLYDELIPLLLEKLHRFFNCIVELRHYRFWSSSLLLVFDGGLNDKEARSNSLDIRMIDFANTIYLQDNPSVDDEYIFGLRNLIKSIQILNNSIHNIYFLPYEITTCFYSENYNIKEVGYHNHFRKSKSAIFERHAKKKKKKNLYVNLELLRNTERAGENYACGVTENGEGKNKNDENNANRKSSKHDAHSNDDKNSKSSKNNENNANSKSCKSNKNKKKISYSSEARSGSNSNWLTQKYANIFAEKKKKKKISNEHKYRNYSECAFRSQTRVSLCRGNNGWGIEGQSVNRTRPNRNNSRESSMCRSRSRSRSRRGNLHGSRFIHTNNDNACSSPLVKVSNDEDHYNGEEIYKNQVNAERSKSFKYSEKIRIQDRNNTNEKGTNKMMRTHLYNEETNNIYYEGLRDIITIKNLEEVNSNAEIYYYSSYKNKNRMKNVSNGGEHRKENSQIDQPYGGKINKEICNIEHRKCKNNIDIPITKRKDGHTQGNVKNKGNVLHNLLELNESRISDIRMNSSQLGYANMINSVEKDKENCVLTNNMNINNDGTKVDYGKGNRDDRSDTKKWMNNIFSEEWNNQNRQKSDKSILLPKRNIIKKEDIAVPFQQLSYDLIIQDIIFKTLKVTSNINRRVKTEVEDMLEISQPPWDNKNVITTHKSNNTYEILSKGGRTQKCKYTNYPDDANGSKNCCKHEHIYMNEGNYRSSCVNFQKKNLKICPNDNFNITHLDVYASEGPNIQEKKTIEDTNLHDQINNYKFFHKDTPKYLNNKEQHTNCTLNNILHDADIINDTTKHESGNKAERSRKKDSTNNLHLLNKNINLKLFINYVIRIEIEKKKRQEEEEERQQLLLDKNKINMKMKNMCTSHFSDSNKDTLELYKKMKKLKLQKSAISKALNSEEKLKKKKKKKIQGGLEVLEEEEEQTNHWNREKKEKKEDPTCKSIEHVLKNVHYNSDTNIIYNNRLRDDYEMANKMNRQNDNIYKRCTSCTDILLSIKKDKKEKKKKPKKNRKRLMRKINIFSRTKSNIIRANRWHSFFNNDRNNRSNRSNTNSRYSRKVSNCGNNNNYYYSNKMRTKLDLMEINKKALKSEWFYKLYDDFNVFSPHYQNSIMCLDDITNRNNILENYLKNKDVHYDCTRKNLIIPLAETNPYYKYLRRSLSEPNFYKFSRFRYVPNDMYDSKKMNYNNLIKNRLYKLSKVPIYNQIYGFTSNSGGTGSSDISLE

>SBT82325.1 inositol polyphosphate kinase, putative [Plasmodium ovale]

MNVEEYRHQVGGHCKLIKPKDSSKVYKPLIENEYIFYEKLTSFGSSSAESGPLHILKKFIPKFYGVADIVVESAPMSGSPRMGKISQSEGKEAEEEAVKEAGKEAEEEAVKEAGKEAEEEAVKEAGKEAEEEAVKEAGKEAEEEAVKEAGKEAEEEAVKEAGKEAEEEAVKEAGKEAEEEAVKEAGKEAEEEAVKEAGKEAEEEAVKEAGKEAEEEAVKEAGKEAGKEAGKEAREEAGEEAGKEADKNRPTVESAQEKDKNVDGNICKRTEKRNKCVPHIVLEDLVYGFRRPCVLDIKMGKRQRKIGASLEKRKRQIEKSFKTTSHSLGFRLCGCQHYDKLSDTLFCKDKYWGRNLSKEKIPWEIKNWFWNGSLLYAELIPLLLKKLHHFFNCIIELRHYRFWSSSLLWVFDGGLNNKKDRSNSLDIRMIDFANTIYLKDNPSADEEYIFGLRNLISSIQIVNYSIHNSYFLPHEITTCFFSENYDLIKKKKKKIGYTYFKNPKRESEPDQDQDQDQDPDSEPELELDQEPEPEPELELDQESKSESKSKSKSESKSESKSSTFDRNTEIQKKKKKNIFSNFEFMKHMNMQKVGKNKRDDYSIGHYFRDSYPLHVQNKRDLVYSEMLPFDREREEAHTSTFPVEDSISHWEERHRRVKKSTEKGHFNESIFPMAVCNSCEDGNHGDKVKYYEHYKIIGEQSSEIKREHAQGNFSEYEHVNYFQNEKNGANTLFRNLSENNGGIPLLLIQEEKQIERTSQKSIAEREEKDNQSFILNTMDSNPHTVDGNPCTADRNTRNKAEGANQSEQGEEQKNDPFAQDINDEGVNCESAGSEKRGKDHVFFKNSFKGKEDMDISSIGIVNEKDEEEKGKKKDMHNSHLSHNSLDTSKLHNKIKKAKLKRNMPNRIISCVEKEHAYMCDQVTLKKLSYGSDSILVYNKAIEQSAGISKLKRRTRAAPARCSTSALTLPSAEGRGTKKKAQRKAKNEGKPSNERRDRRRDRRRNRHRDKYSEKKARKERMAKVNTLTNEKRGILGGSPPKRFNSPFLPMIDAIRGGEFLNGIWGRRPSDEFNISSLEFKNSSACIDDVTKENKTKLILTDEMVHNNLTRKNLIIPLTESNEHYKYLRRSFSEPNFYNFSRVRCTHNSAFYDTKINYSQLINNRIDKMMRVPIYKEIYGFTANTSDTDSSALSSTH

>XP_005248900.1 inositol hexakisphosphate kinase 3 isoform X2 [Homo sapiens]

MSLLEGRITMHFIPEVDAQAELSTDPAKALLRSEPHLNTPAFSLVEDTNGNQVERKSFNPWGLQCHQAHLTRLCSEYPENKRHRFLLLENVVSQYTHPCVLDLKMGTRQHGDDASEEKKARHMRKCAQSTSACLGVRICGMQVYQTDKKYFLCKDKYYGRKLSVEGFRQALYQFLHNGSHLRRELLEPILHQLRALLSVIRSQSSYRFYSSSLLVIYDGQEPPERAPGSPHPHEAPQAAHGSSPGGLTKVDIRMIDFAHTTYKGYWNEHTTYDGPDPGYIFGLENLIRILQDIQEGE
